# Comprehensive Insights into Medicinal Research on Imidazole-Based Supramolecular Complexes

**DOI:** 10.3390/pharmaceutics15051348

**Published:** 2023-04-27

**Authors:** Shu-Rui Li, Yi-Min Tan, Ling Zhang, Cheng-He Zhou

**Affiliations:** 1 Institute of Bioorganic & Medicinal Chemistry, Key Laboratory of Applied Chemistry of Chongqing Municipality, School of Chemistry and Chemical Engineering, Southwest University, Chongqing 400715, China; 2 School of Chemical Technology, Shijiazhuang University, Shijiazhuang 050035, China

**Keywords:** imidazole, supermolecule, complex, anticancer, antibacterial, probe

## Abstract

The electron-rich five-membered aromatic aza-heterocyclic imidazole, which contains two nitrogen atoms, is an important functional fragment widely present in a large number of biomolecules and medicinal drugs; its unique structure is beneficial to easily bind with various inorganic or organic ions and molecules through noncovalent interactions to form a variety of supramolecular complexes with broad medicinal potential, which is being paid an increasing amount of attention regarding more and more contributions to imidazole-based supramolecular complexes for possible medicinal application. This work gives systematical and comprehensive insights into medicinal research on imidazole-based supramolecular complexes, including anticancer, antibacterial, antifungal, antiparasitic, antidiabetic, antihypertensive, and anti-inflammatory aspects as well as ion receptors, imaging agents, and pathologic probes. The new trend of the foreseeable research in the near future toward imidazole-based supramolecular medicinal chemistry is also prospected. It is hoped that this work provides beneficial help for the rational design of imidazole-based drug molecules and supramolecular medicinal agents and more effective diagnostic agents and pathological probes.

## 1. Introduction

Intermolecular interactions have been playing a positive role in modern science for several decades [1]. Research and potential applications toward supramolecular species have been rapidly expanding and active scientific topics, strongly promoting the development of supramolecular science at a revolutionary pace. Numerous efforts have been devoted to the study of supramolecular chemistry for its possible potential, which have been expanded into many disciplines such as materials science, biology, medicine, and pharmaceutical science. A supramolecular interaction based on weak bonds (coordination bonds, hydrogen bonds, ionic bonds, van der Waals forces, etc.) has been widely used in molecular recognition [2,3], organic catalysis [4,5], fluorescence imaging [6,7], magnetic resonance imaging (MRI) [8,9], medicinal agents [10,11], and other fields. Especially regarding the medicinal aspects, many supermolecules formed by two or more molecules, such as anticancer trimolecular supramolecular complex **I1**, bimolecular supermolecule **I2**, MRI contrast agents **I3** and **I4**, etc., have been widely used in clinical settings, showing great potential for the development of supramolecular species [1,9,10].

Importantly, the formation of supermolecules by noncovalent bonds can interfere with the function of living molecules in biological systems and make drug molecules with better solubility, higher bioavailability, better biocompatibility, better drug-targeting, fewer multidrug resistances, lower toxicity, fewer adverse effects, and better curative effects. For example, recent research [12] revealed that the artificial aminothiazolquinolone **I5** was able to form a compound **I5**−Cu^2+^−DNA ternary supramolecular complex, in which the Cu^2+^ ion acts as a bridge between the backbone of 3-aminothiazolquinolone and the phosphate group of the nucleic acid and, thus, exhibited potent antibacterial activity, low cytotoxicity to hepatocyte cells, strong inhibitory potency to DNA gyrase, and a broad antimicrobial spectrum including against multidrug-resistant strains. Anticancer drug adriamycin **I6** with *β*-cyclodextrin could form a 1:1 organic supramolecular complex, which helpfully improved the water solubility of adriamycin **I6** and, thus, resulted in its better bioavailability. Moreover, supramolecular species were relatively easily obtained with several advantages: a lower cost, a shorter time period, and a higher potential as clinical drugs with successful research and development. Therefore, many increasing efforts were recently directed toward supramolecular species to explore their possible potential [11,13]. Azole heterocycles [14,15,16] such as imidazoles [17,18,19], triazoles [20,21,22,23], tetriazoles [24,25], thiazoles [26,27,28], oxazoles [29,30], benzimidazoles [31,32], benzotriazoles [33,34], and carbazoles [35,36,37,38] have been increasingly paid attention in recent years because they are the ideal building blocks for constructing supramolecular species due to their unique electron-rich aromatic structures with nitrogen, oxygen, and/or sulfur atoms that are capable of exerting multiple noncovalent bonds to form supramolecular species. These azoles were hybridized with sulfonamides [39,40,41], naphthalimides [42,43,44], coumarins [45,46,47,48], quinolones [49,50,51], quinazolones [52,53,54], berberines [55,56,57,58], indoles [59,60,61], purines [62,63], aloe emodins [64,65,66], pyrimidines [67,68], etc., to afford derivatives with excellent manipulation in forming biosupermolecules, which could effectively regulate the behavior of biomolecules in living systems and, thus, show a variety of good biological activities.

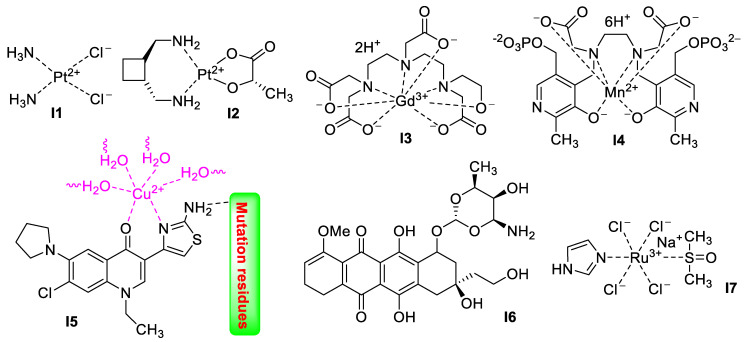



**Scheme 1:**
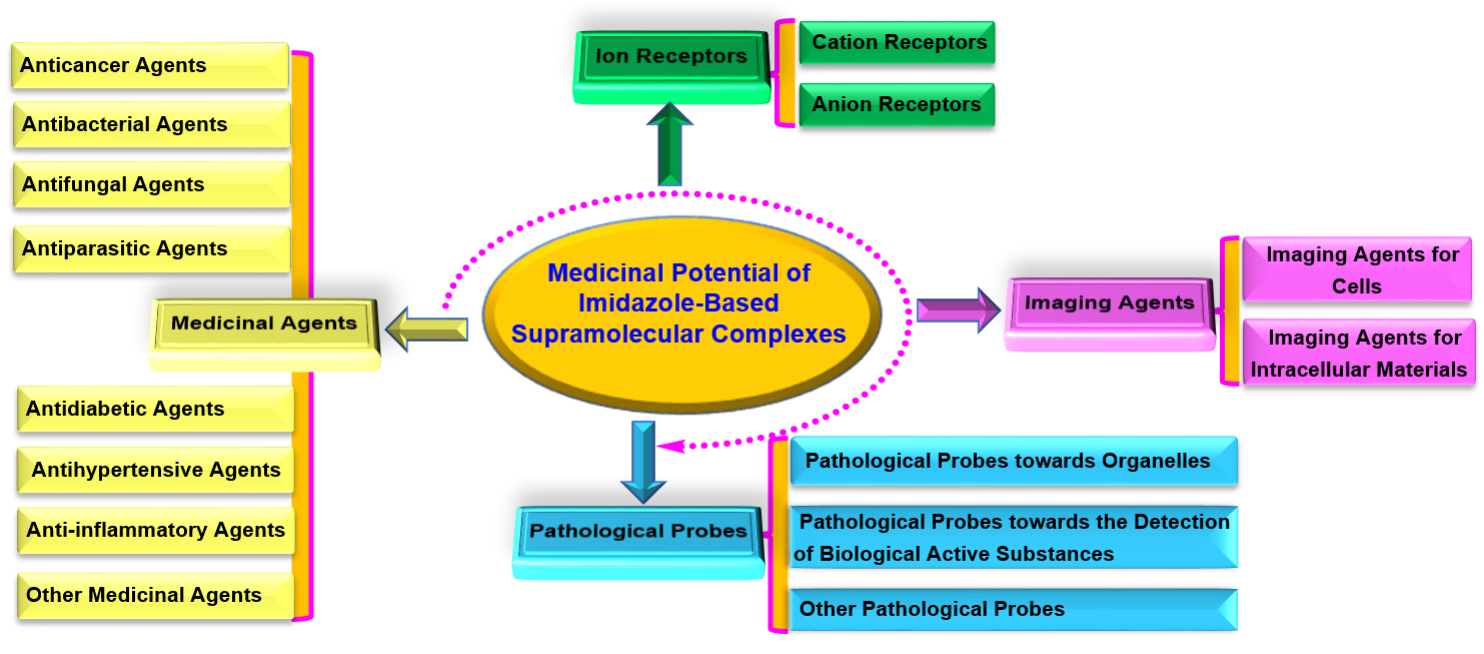
The general scheme covering this review for medicinal potential of imidazole-based supramolecular complexes.

Among the various azoles, imidazole containing two nitrogen atoms is an important and unique five-membered aromatic heterocycle present in many biomolecules such as histamine and histidine. It exists in two equivalent tautomers, in which hydrogen atoms can be located on either of the two nitrogen atoms [17,69]. Such electron-rich nitrogen heterocycles not only readily accept or donate protons but also exert a variety of weak interactions. Importantly, multiple binding sites of the imidazole ring can easily form imidazole supramolecular complexes with various inorganic or organic ions and molecules through noncovalent interactions, showing not only the biological activity of imidazole drugs themselves but also the many advantages of supramolecular drugs, which may naturally exert dual or multiple mechanisms to overcome resistance [17,18]. Many imidazole supramolecular complexes showed good safety, low toxicity, few adverse reactions, high bioavailability, strong drug targeting, good biocompatibility, and high efficacy. In addition, imidazole-based supramolecular complexes are widely available and easy to prepare, which makes the development cost low, the cycle short, and the possibility of druggability large, so they have been attracting increasing attention. Furthermore, many metal supramolecular complexes formed by imidazole derivatives and metal ions such as platinum, gold, silver, palladium, ruthenium, copper, iron, rhenium, vanadium, iridium, manganese, cobalt, stannum, erbium, ytterbium, gallium, etc., have revealed promising clinical potential. In particular, ruthenium imidazole complex **I7** entered phase I clinical trials as early as 1999 and was used to inhibit the metastases of lung cancer and breast cancer cells [70]. Therefore, the use of imidazole to design drug molecules, develop imidazole supramolecular agents, and explore their potential applications has become quite an active subject in the field of medicinal chemistry in recent years. Combined with our work with imidazole and referring to other examples from the literature in the past 15 years, this work systematically reviews the medicinal progress and potential of imidazole-based supermolecules, including anticancer, antibacterial, antifungal, antiparasitic, antidiabetic, antihypertensive, and anti-inflammatory medicinal agents, as well as ion receptors, imaging agents, and pathologic probes. The general scheme of this review is presented in Scheme 1. Moreover, the new trend of the foreseeable research in the near future toward imidazole-based supramolecular medicinal chemistry is also prospected.

## 2. Imidazole-Based Supermolecules as Medicinal Agents

A five-membered imidazole ring is an important part of the histidine and histamine molecules present in a large number of biomolecules such as hemoglobin and hemocyanin, in which the imidazole ring exerts various noncovalent interactions through coordination bonds, hydrogen bonds, ionic bonds, dipole–dipole and π–π interactions, etc., to form supramolecular species; these biosupermolecules are essential for the maintenance of life and the reproduction of organisms. The special biofunctional behavior of the imidazole ring in a living system has been widely employed to design new drug molecules, and recently an increasing interest has been directed toward imidazole-based supramolecular complexes as possible medicinal agents because such supramolecular medicinal drugs often show not only dual or even multiple mechanisms of action, which are helpful to overcome drug resistance, but also good safety, low toxicity, few adverse reactions, high bioavailability, strong drug targeting, good biocompatibility, and high efficacy [17]. Therefore, many efforts are being focused on the research and development of imidazole-derived supermolecules as medicinal agents, which have been revealed to display wide medicinal potential as anticancer, antibacterial, antifungal, antiparasitic, antidiabetic, antihypertensive, and anti-inflammatory medicinal agents [11]. 

### 2.1. Imidazole-Based Supermolecules as Anticancer Agents

Cancer has become one of the serious diseases that increasingly threaten human health. Although many effective clinical drugs [71] such as alkylating agents [72,73], metal and macrocyclic drugs [74,75,76], etc., have been successfully developed, the frequent emergence and diversity of cancer as well as the high toxicity and low selectivity of anticancer drugs mean the current clinical drugs are far from meeting the needs of clinical use. The development of new anticancer drugs has become quite urgent and is also one of the most heavily invested in pharmaceutical developments worldwide [77,78,79]. Imidazole compounds display a wide anticancer potential. In particular, imidazole-based supermolecules have attracted extensive attention and have become one of the new research hotspots for anticancer drugs due to their strong activity, good selectivity, safety, and multitargeting properties [80].

#### 2.1.1. Noble Metal-Based Imidazole Supermolecules as Anticancer Agents

Noble metal-based supramolecular drugs have achieved great success. In particular, platinum anticancer drugs such as cisplatin, carboplatin, and many other metal supramolecular complexes have been widely used in clinical settings. This has encouraged countless researchers to develop new cancer agents with precious metals [81,82,83]. With the wide application of imidazole drugs in the field of medicine, more and more imidazole-based supermolecules have been developed as anticancer agents.

##### Platinum-Based Imidazole Supermolecules as Anticancer Agents

Platinum-based metal supramolecular complexes as anticancer agents are one of the most deeply investigated metal supermolecules. Since the discovery in 1969 of supramolecular cisplatin, a three-molecule complex formed by one molecule of PtCl_2_ and two molecules of NH_3_, which shows anticancer activity, many platinum-based supramolecular complexes, such as carboplatin, sunpla, oxaliplatin, nedaplatin, and lobaplatin, have been successfully developed and widely used as anticancer drugs in clinical practice [84]. A broad anticancer spectrum and strong efficacy further stabilize their indispensable status in anticancer treatments. However, platinum-engaged anticarcinogens are confronted with severe side effects such as nephrotoxicity, gastrointestinal toxicity, and neurotoxicity. Furthermore, a relatively weak water solubility, a difficult metabolism, and poor activities against breast cancer and colon cancer hinder their clinical application. Therefore, there is an urgent need to develop new and more efficient anticancer drugs [85]. Recent studies revealed that imidazole-based supermolecules show a large potential as anticancer agents.

The imidazole ring has a remarkable coordination affinity for Pt (II) ions, and its complexes could inhibit tumor growth by interacting with DNA. Platinum complex **II1** with an imidazole functionalized at the N^1^ atom surpassed cisplatin in the treatments of DLD-1 (colorectal) and MCF-7 (breast) cell lines. The half maximal inhibitory concentration (IC_50_) values of **II1** were 57.4 μM for DLD-1 and 79.9 μM for MCF-7, while cisplatin was far less active when confronted with resistance. Based on the fact that p53 was induced in the exposure of the compound, which was correlated to the interaction with nuclear DNA, complex **II1** might partake in the mitochondrial apoptotic process free from the p53 gene [86].

A bidentate *N*,*N* chelating ligand and 1-alkyl-2-(arylazo) imidazole were used to synthesize Pt (II) complexes **II2a–b**. Long alkyl-substituted imidazole platinum complex **II2-b** showed a significant cytotoxic effect on MCF-7 cell lines and displayed a higher cytotoxic effect than complex **II2-a** against all cancer cell lines (A549, MCF-7, and CACO2) [87].

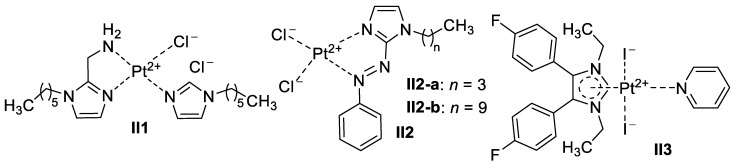



A double *p*-fluorophenyl imidazole platinum complex **II3** displayed much higher anticancer activities than cisplatin, especially in hepatocellular carcinoma (HCC) cancer cells. Complex **II3** was confirmed to be effective as an immunogenic cell death inducer in HCC treatment. Its efficacy was attributed to targeting the HCC antioxidant network. Complex **II3** rendered a new scaffold to develop chemotherapeutics capable of inducing immune responses against solid tumors [88].

A cytotoxicity evaluation identified Pt (II) imidazole compound **II4** as the most promising compound, with lower IC_50_ values, down to 0.86 μM and 0.48 μM against MCF-7 and MDA-MB-231 cell lines, respectively. Further investigation revealed that complex **II4** might exert its antitumor activity by activating p53 and proline oxidase (POX) to cause an increase in the level of intracellular reactive oxygen species (ROS), up-regulating the pro-apoptotic proteins and caspase cascade to mediate the intrinsic and extrinsic apoptosis. Moreover, supermolecule **II4** conjugated with a dendrimer could significantly reduce the effect of the drug efflux responsible for drug resistance. These results suggested that complex **II4** might serve as a promising candidate in the development of a novel therapeutic agent to treat breast cancer [89].

Metal complexes featuring imidazole-involved nitrogen heterocyclic carbenes are among the basic scaffolds in anticancer agents. A four *p*-methoxy-benzyl-substituted diimidazole Pt (II) complex **II5** demonstrated high antitumor activities against HT-29 human colon adenocarcinoma, DLD-1 colorectal adenocarcinoma, and the KB-V1/Vbl cervix cancer cell line [90]. 

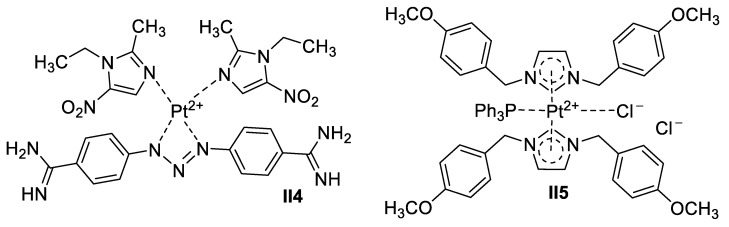



Aniline-incorporated benzimidazole Pt (II) complex **II6** was stable and controlled a wide range of anti-carcinoma spectra, which was a lethal agent against mammary cancer, rectal cancer, and hepatocellular carcinoma cells. In particular, it displayed the most potent anti-breast cancer cell line activity with an IC_50_ value of 12.4 μM, which is almost rivaled by cisplatin. On the contrary, Pd (II) supermolecules were quite unstable for their high rate of ligand exchange. With the Pd (II) ion as the central ion, the anticancer activity of the complex was enhanced. This was mainly because the formed Pd (II) complex was less stable than the Pt (II) complex and easily exchanged ligands with biological macromolecules. Therefore, it was very important to select the appropriate ligands to keep the structural integrity of the complex before reaching the target [91].

Platinum-based supramolecular complexes **II7a–b**, formed by two molecules of benzimidazoles and one molecule of PtCl_2_, showed good anticancer activity. In particular, the unsubstituted Pt (II) complex **II7-a** on the benzimidazole ring was far superior to the reference drug carboplatin in inhibiting the MCF-7 and HEP2 cell lines of larynx carcinoma. When different alkyl chains were introduced into the C-2 position of benzimidazole, the complexes showed good inhibitory activity against cervical carcinoma HeLa, breast cancer MCF-7, and breast cancer MDA-MA-231. The activity of complex **II7-b** was more remarkable than that of carboplatin when the C-2 position of benzimidazole was replaced by the *p*-chlorobenzyl group, which indicated that the introduction of chlorine atoms was beneficial to enhance the activity [92,93]. 

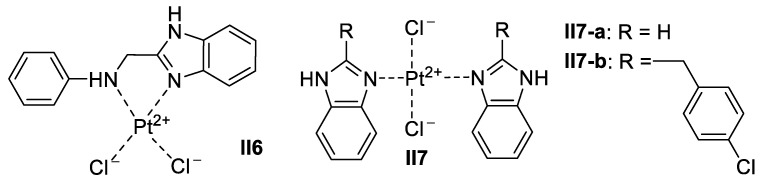



##### Gold-Based Imidazole Supermolecules as Anticancer Agents

Among all the drugs containing metals other than platinum, gold compounds have turned out to be a cutting-edge class of anticancer compounds [94]. The supramolecular anticancer mechanism of the gold complex is different from that of cisplatin. In recent years, the research on the supramolecular anticancer effect of the gold complex has mainly focused on the imidazole nitrogen heterocyclic carbene of gold [95,96]. Metal organic compounds can inhibit the activity of thioredoxin reductase and other proteins, leading to the death of cancer cells. Therefore, the construction of a nitrogen heterocyclic carbene-based metal supermolecule is of great significance for the development of anticancer drugs.

Gold (I) imidazole derivatives **II8a–b** displayed good inhibitory activity against A2780cisR ovarian cancer cells and cisplatin-resistant A2780cisR ovarian cancer cells. A biological assay showed that a nitrogen heterocyclic carbene containing chlorinated ligands had lower anticancer activity than pyrimidine ligands. The inhibitory ability of **II8-b** containing pyrimidine was similar to that of cisplatin, with an IC_50_ of about 3 μmol/L against A2780cisR ovarian cancer cells. The activity of **II8-b** was twice that of chlorine-containing ligand **II8-a**, and the inhibitory activity of **II8-b** against cisplatin-resistant A2780cisR cells was 10 times that of cisplatin. Structure-activity relationship studies showed that nitrogen heterocyclic carbene containing chlorinated ligands had lower anticancer activity than pyrimidine ligands. This was mainly due to the unstable binding of chlorine to gold, making chlorine very reactive and easily inactivated by active components in cancer cells. In the selective experiments, compound **II8-b** with high anticancer activity strongly inhibited the activity of cytoplasmic thioredoxin reductase TrxR1 (IC_50_ = 4.9 nmol/L). However, its inhibitory ability on mitochondrial matrix thioredoxin reductase TrxR2 was weakened, with an IC_50_ value greater than 100 nmol/L, indicating that the target of gold (I) nitrogen heterocyclic carbene **II8-b** may be the cytoplasmic thioredoxin reductase TrxR1 in cancer cells [97].

Dimethyl imidazole onium Au (III) compound **II9**, which features in a stable pincer-type compound, was given in an anticancer exploitation that could target multiple proteins. When the methyl groups were replaced by the ethyl and propyl groups, the antitumor activity against HCT116, NCI-H460, and HeLa cells was enhanced; for example, the IC_50_ value for HeLa dropped from 2.04 μM to 0.36 μM. Coincidentally, Pt (II) and Pd (II) analogues could also bind with the target proteins and display a comparable antitumor activity against cancer cells. This result highlighted the structural importance of pincer-type metal compounds in their anticancer activities. Notably, the persisting drug resistance can hopefully be overcome for multitargeting mechanism of action [98].

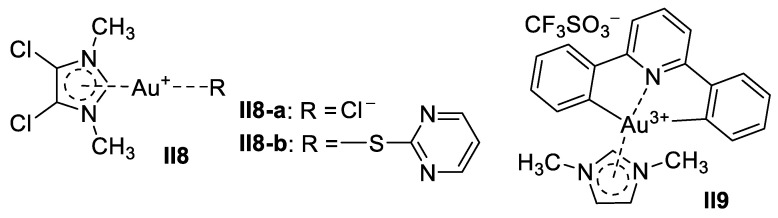



Basal-like breast cancer is a very aggressive subtype of breast cancer that gives few chances of survival. Among all the classes of gold compounds, the gold (I) phosphine complexes are potential candidates for anticancer therapy. Imidazole-based phosphine gold (I) complexes **II10a–b** exhibited superior anti-breast cancer activity in vitro to standard drug cisplatin after a 24 h treatment, as shown in the table. The results showed that cisplatin was less effective than compounds **II10a–b** in both A17 and MDA-MB-231 cells after a 24 h treatment, suggesting that the introduction of azole ligands with polar groups could be critical in determining the fate of the anticancer properties of these new gold-based drugs [99].

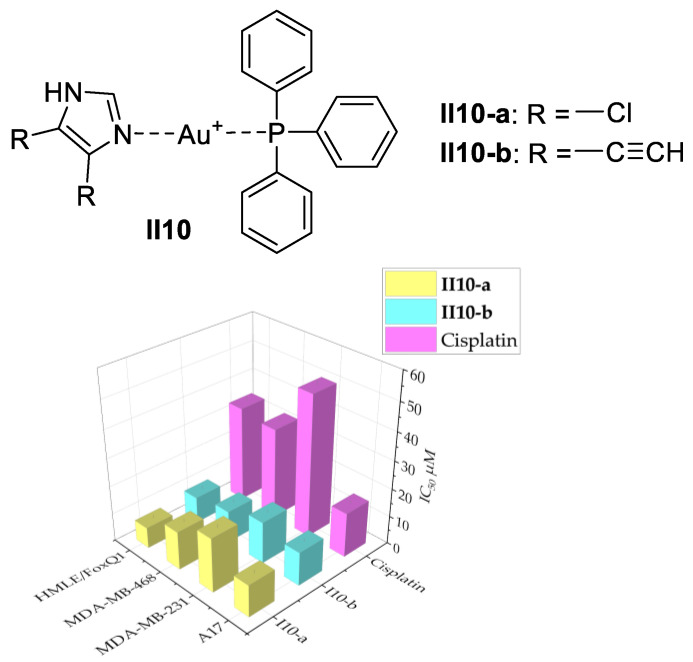



The bioconjugation of chemotherapeutics, including metal-containing anticancer agents, to glucose-like or glycomimetic substrates served as a new method of semi-targeted chemotherapy. The combination of biomolecules overexpressed in cancer cells with gold complexes made them semi-targeted metabolites. Auranofin, an anti-arthritis agent, encompassed this property and exhibited antitumor activities. The reactions of 1,3-dibenzyl-4,5-diphenyl-imidazole-2-ylidene gold (I) chloride with pre-synthesized glycosyl thiols under mildly basic conditions gave the desired gold (I) thiosugar imidazole complexes with high to excellent yields (79–91%). The thiosugar gold (I) compound **II11** retained the strong and redox-active Au-S bond contained in auranofin, showing good activity in the low micromolar to nanomolar region against the tested cell lines [100].

Gold (I) complexes in the form of boron-dipyrromethene diimidazole ligands were proven to have a high potential in inhibiting MDA-MB-231 (breast), PC3 (prostate), and SW480 (colon) carcinoma cell lines, almost comparable to that of auranofin. In particular, gold (I) imidazole bimetallic complex **II12** was found to exhibit a superior anticancer potency toward SW480 to etoposide and 5-fluorouracil (IC_50_ = 16 and 26 μM, respectively) [101].

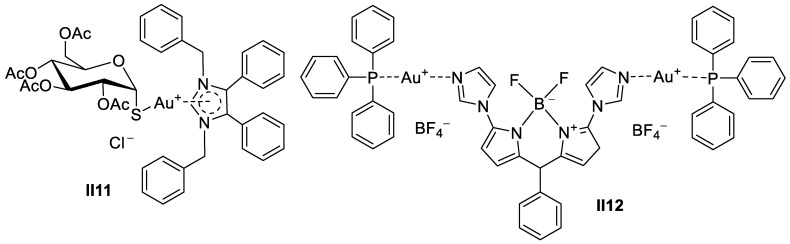



Gold (I)-bridged diimidazole complex **II13** was very stable at a physiological pH and displayed a two-fold stronger inhibitory ability in ovarian cancer A2780 cells in comparison to cisplatin. Moreover, compound **II13** controlled a much lower IC_50_ value of 0.75 μM in cisplatin-resistant ovarian cancer A2780cisR cells than that of cisplatin. These results indicated that compound **II13** was a highly potential antiproliferative agent and deserved deeper pharmacological and anticancer mechanistic studies [102].

Different types of gold (I) imidazole compounds based on 4, 5-bis(4-methoxyphenyl) imidazole were designed and synthesized to tackle endometrial cancer (EC). The biphenyl imidazole gold (I) compound **II14** was 10 times more potent than cisplatin or auranofin against Ishikawa cells. In vivo studies showed that compound **II14** possessed a stronger anticancer effect than auranofin in the xenograft model of EC. Mechanistically, compound **II14** could suppress the expression of thioredoxin reductase and nuclear factor E2-related factor 2 in vitro and in vivo, which were essentially involved in EC development. Therefore, gold (I)-based compound **II14**, which showed a strong anticancer activity, had the potential to be considered as a promising choice for the treatment of EC [103].

Similarly, iodine-substituted dimethoxyphenyl imidazole gold (I) compound **II15** was the most active compound (HepG2, IC_50_ = 0.50 μM; SMMC-7721, IC_50_ = 0.92 μM; Hep3B, IC_50_ = 0.52 μM), which was at least two-fold more cytotoxic than cisplatin against hepatocellular carcinoma (HCC) cells. Compound **II15** showed a significant anticancer effect in comparison with cisplatin in a HepG2 xenotransplantation mouse model and could improve CCl_4_-induced chronic liver cancer injury in in vivo studies [104].

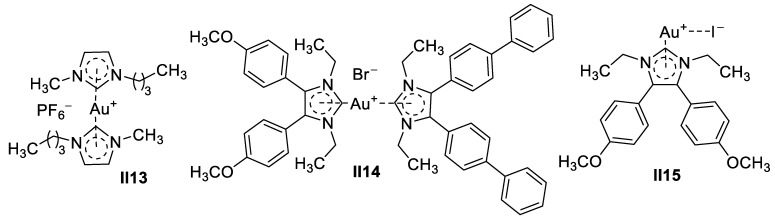



Cyclic nitrogen heterocyclic carbene **II16** formed by bimolecular diimidazole and gold ions displayed a broad antitumor spectrum, which could effectively inhibit breast cancer MCF-7 cells, colorectal cancer HCT116 cells, prostate cancer, prostate adenocarcinoma PC3 cells, and the proliferation of ovarian cancer SK-OV3 cells. The structure-activity relationship showed that the substituents on the triazole ring linked to imidazole and the valence of gold ions had significant effects on the anticancer activity of gold supermolecules. When the ligands were the same, the activity of gold (I) supermolecules was higher than gold (III) supermolecules. When the valence of the gold ions was the same, the substituents on the triazole ring had a significant effect on the anticancer activity. The substitution of long fatty ether chains was more beneficial for anticancer activity than aromatic rings. Notably, compound **II16** exhibited a strong ability to inhibit breast cancer MCF-7 cells, with an IC_50_ value of 0.09 μg/mL, which was 100 times more active than the ability of cisplatin. In addition, compound **II16** showed no cytotoxicity to normal EPC cells (IC_50_ > 100 μg/mL), indicating that compound **II16** had a good selectivity for cancer cells, which is valuable for further study [105].

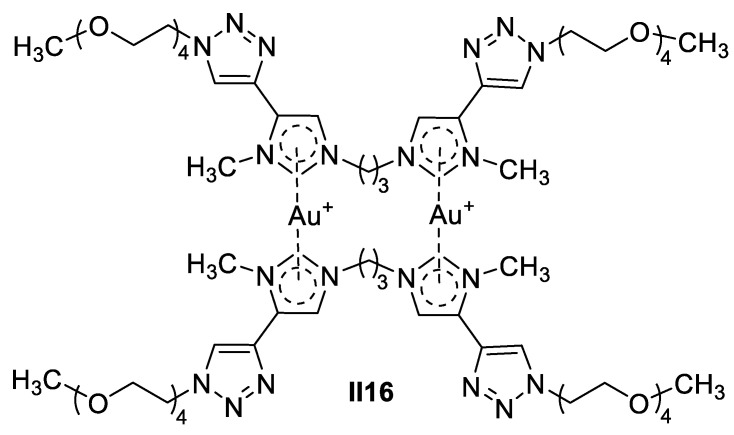



The phenyl-ring-thickened imidazole also used gold-bridged pyrazole to form gold nitrogen heterocyclic carbene derivatives **II17a–c** composed of pyrazole and benzimidazole, which showed good inhibitory activity against non-small cell lung cancer NCI-H1666 cells, especially **II17-a**, with an IC_50_ value that was 0.2 μmol/L and more than 20 times that of cisplatin. Studies showed that the *N*-site alkylation of benzimidazole containing secondary carbon substituent gave better activity than the corresponding primary carbon and asymmetric substituents. For example, the activities of **II17-b** and **II17-c** against NCI-H1666 cells were respectively one-third and one-eighth of that of **II17-a**. In addition, antitumor activity decreased significantly as benzimidazole was replaced with weak-coordinated 4-dimethyl aminopyridine. This might be assigned to the fact that the ligand was separated from the supramolecular complex before it could bind with the target thioredoxin reductase (TrxR). Therefore, the stability of supermolecules played an important role in its anticancer activity [106].

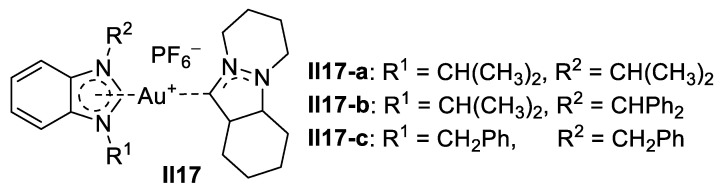



##### Silver-Based Imidazole Supermolecules as Anticancer Agents

Silver compounds have a long history as antibacterial agents. In particular, silver sulfadiazine (AgSD) is widely used as an antibacterial agent for severe burns. Sulfadiazine has no strong antibacterial activity, but it shows strong antibacterial activity together with silver. Therefore, research on silver derivatives is very active within the anticancer field, especially for the imidazole nitrogen heterocyclic carbene silver derivatives [107].

With the aim of combining the potential anticancer properties of both clotrimazole, an imidazole-based antifungal agent, and silver (I) carbenes, silver (I) complexes were developed and evaluated for their anti-proliferative activity against pancreatic adenocarcinoma cell line Panc 10.05. Most complexes were less potent toward Panc 10.05 than cisplatin, but hydroxyethyl-substituted Ag (I) imidazole complex **II18** was as potent as cisplatin and more selective toward Panc 10.05 [108].

Diimidazole silver (I) complex **II19a–d** had remarkable inhibitory activity in rectal cancer cells, in which **II19-c** had the highest activity with an IC_50_ value of 1.7 μmol/L. The anti-rectal cancer activity of **II19-b**, obtained by replacing the 3-cyanophenyl group in **II19-d** with a hydrogen atom, was about two times that of **II19-d**. Supermolecule **II19-a** had strong anticancer activity against cisplatin-resistant ovarian A2780cisR cells [109,110]. The supermolecule obtained by replacing all the substituents with the aminomethyl groups had no cytotoxicity for normal embryonic cells, showing that this supermolecule was selective to cancer cells and had the potential to become a drug for treating cisplatin-resistant cancer. Moreover, the type of pairing ions also had a certain impact on the anticancer activity. The pairing anions of **II19c–d** with similar structures were bromine ion and hexafluorophosphate, respectively. Although the chelating effect of these two ions was the same as that of metal ions, the silver ion was too stable to be released after **II19-d**, and the anticancer activity was weaker than that of **II19-c** [111,112].

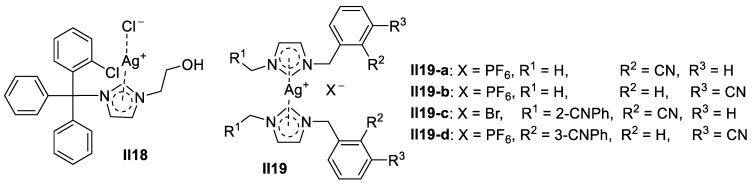



Compound **II20**, formed by phenyl-bridged diimidazole and silver (I), showed a high inhibitory capacity in rectal cancer cells. The IC_50_ value was 1.1 μmol/L, and the activity was four times that of the reference drug 5-fluorouracil. The binuclear imidazole silver (I) compound **II21**, with a similar ligand structure to **II20**, also displayed good anticancer activity, and its IC_50_ value for leukemia K562 cells was 0.9 μmol/L. By contrast, if the phenyl ligands were substituted at the ortho-position, the activity of compound **II21** against leukemia K562 would be decreased by six times [107,113].

Cyclic derivative **II22**, formed by methylene-bridged diimidazole and silver (I), showed activity against leukemia K562 cell lines comparable to that of 5-fluorouracil. In the meantime, there was a dose-dependent effect similar to that of 5-fluorouracil. The derivative obtained by changing the 2-position cyano group on the benzene ring to the 3-position gave an inhibitory effect for rectal cancer HCT116, and the activity was similar to that of 5-fluorouracil [114].

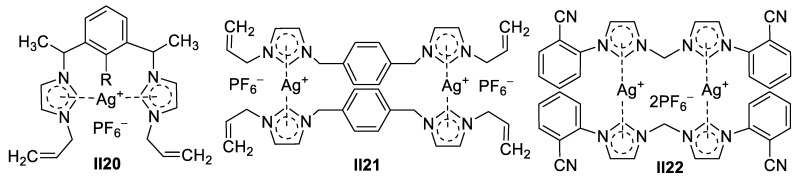



Mono imidazole silver (I) acetate and its biimidazole silver (I) hexafluorophosphate analogue (**II23** and **II24**) have been delved into for their potential anticancer activity toward MCF-7, with IC_50_ values that were proven at the nanomolar level. Compound **II23** exhibited proficient activity, with an IC_50_ value of 13.28 nM against human breast adenocarcinoma cell line MCF-7, and compound **II24** demonstrated the remarkable anticancer potential, with an IC_50_ value of 10.39 nM. The antibacterial activity of silver mono-ammoniate (I) and silver double-ammoniate (I) compounds was not significantly different, but the former had a better anticancer potential than the latter [115].

Anthracene-substituted dinuclear Ag (I) imidazole compound **II25**, bearing two anthracenyl fluorescent probes, showed potent cytotoxic effects in vitro toward human SH-SY5Y neuroblastoma cells, with an IC_50_ value of 1.059 ± 0.042 μM, and was 10-fold more potent than cisplatin (IC_50_ = 9.987 ± 0.506 μM). The interactions with model biological targets showed that compound **II25** was able to bind the *C*-terminal dodecapeptide of thioredoxin reductase TrxR and strongly inhibited the activity of this enzyme. Cellular uptake experiments demonstrated that compound **II25** much more easily entered cells, with its uptake being about 10-fold greater than that of cisplatin. The increased cell internalization of compound **II25** was probably correlated with its higher lipophilicity. In turn, the greater cell internalization might explain the higher cytotoxicity [116].

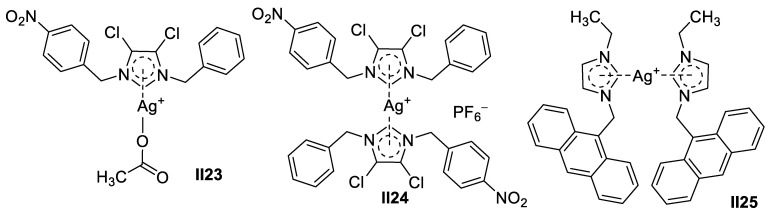



Two silver (I) imidazole derivatives **II26a–b** with chiral isomerism displayed a lower anticancer activity than cisplatin and gemcitabine. Two structure-activity correlations were found. First, when the volume of R-alkyl increased, the activity of silver (I) imidazole carboxylate salts species decreased. Second, an interesting chirality–anticancer relationship was detected when comparing the activity of an enantiomerically related compound. Compound **II26-a** showed a better cytotoxic activity than its enantiomeric derivative **II26-b** [117].

Miconazole is a highly effective, safe, and broad-spectrum antifungal drug, which has an effect on almost all pathogenic fungi. Two silver (I) complexes **II27a–b** derived from the biologically active ligand miconazole were synthesized. Human HepG2 cells were more sensitive to compounds than murine Balb/c 3T3 fibroblasts. Complexes with miconazole significantly inhibited all the endpoints of the HepG2 cells starting at 0.1 μM. Moreover, these complexes inhibited the growth of cancer cells at sub-micromolecular concentrations (0.26–0.47 μM), lower than those of cisplatin [118].

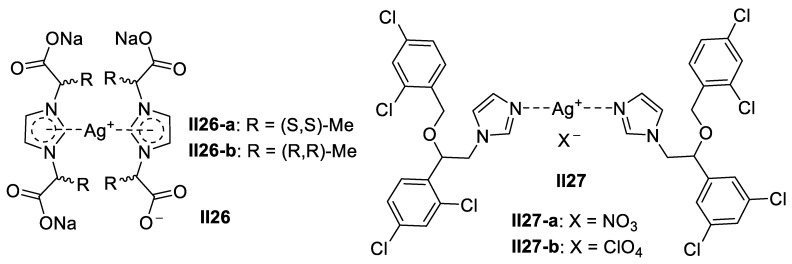



The propyl benzimidazole Ag (I) compound **II28** exhibited anti-proliferative activity on MCF-7 and MDA-MB-231 breast cancer cells. When the normal propyl group was replaced by isopropyl and hydroxypropyl fragments, the resulting compounds also showed good anticancer activity. Considering that these silver compounds were more active compared to cisplatin for 24 and 72 h incubation, these compounds might be suggested as an alternative agent to cisplatin. The biological activity depended on the nature of the group bound to benzimidazole and the cancer cell line [119].

Allyl-substituted benzimidazole Ag (I) compounds **II29a–b** showed dose- and time-dependent cytotoxic activity against human breast and prostate cancer cell lines (MCF-7, MDA-MB-23, and DU-145) and L-929 normal cells. MDA-MB-231 and MCF-7 human breast carcinoma cells were the most sensitive, displaying an IC_50_ lower than 1 μM at all time points for phenyl-substituted derivative **II29-a** and naphthalene-substituted compound **II29-b**, respectively. The IC_50_ values for Ag (I) benzimidazole compounds were higher in normal cells, especially compared to those of breast cancer cells, suggesting that these compounds possessed noteworthy selectivity for human breast cancer cells. Benzene-substituted compound **II29-a** showed high selectivity (13-fold) for MDA-MB-231 breast cancer cells at all time points, while these results also demonstrated that naphthalene-substituted compound **II29-b** had four–seven-fold selectivity against MCF-7 breast cancer cells [120].

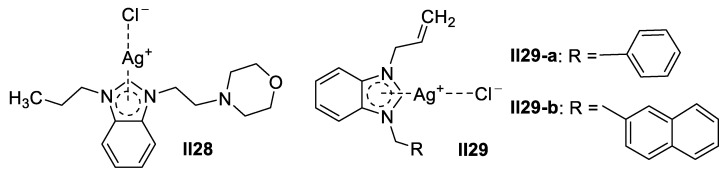



Isobutene-substituted benzimidazole silver (I) compounds **II30a–b** showed appreciable activity in MCF-7 and DU-145 cells and similar activity in MDA-MB-231 cells. As the cytotoxicity of **II30a–b** had larger IC_50_ values for non-cancerous cell lines, these compounds were more effective in breast cancer cells and prostate cancer cells. The in vitro antitumor activity of compound **II30-a** was found to be lower than that of compound **II30-b**. After 24 h, xylene-substituted compound **II30-b** was 12.42-fold more potent against MCF-7 cells, 7.70-fold more potent against DU-145 cells, and 11.94-fold more potent against MDA-MB-231 cells [121].

Dipentyl benzimidazolium silver (I) compound **II31** showed good anticancer activity, with IC_50_ values comparable to those of reference drugs, and was far lesser cytotoxic to normal cells (EA. hy926) compared to cancer cells (MDA-MB-23 and HCT 116). While the silver (I) compound exhibited strong cytotoxic activity against both cell lines, compound **II31** displayed dose-dependent cytotoxic activities against both cell lines. A mechanistic study revealed that cancer cell death was governed by apoptosis via the mitochondrial pathway [122].

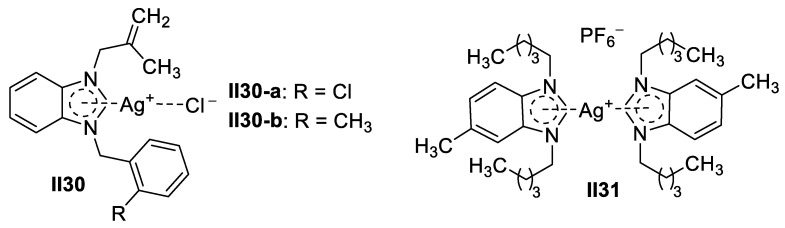



Cyclic derivative **II32**, formed by tetramethylene-bridged dibenzimidazole and silver compounds, was designed and synthesized. Interestingly, increasing the length of the alkyl chain from *n* = 2 to 9, for the ligands and their respective compounds, showed trends in increased cytotoxicity against the human colon cancer cell line. All the series of Ag (I) compound **II32** showed superior activity compared to the standard drug 5-FU against HCT 116 cell lines. Cytotoxicity data showed that tetramethylene-linked dibenzimidazole salts and their respective dinuclear Ag (I) compounds can be useful therapeutic agents against colon cancer [123].

Compound **II33**, formed by two phenyl-bridged benzimidazoles, suppressed the proliferation of both rectal cancer HCT116 cells and leukemia K562 cell lines. The structure-activity relationship revealed that the anti-rectal cancer activity first increased and then decreased with the growth of the *N*-alkylated chain’s length. The activity against rectal cancer peaked when *n* was 4, giving an IC_50_ value of 0.01 μM. Considerable activity was also observed when *n* was 8, 9, and 10, with IC_50_ values all at around 4 μM; this was much more active than 5-fluorouracil, which had an IC_50_ value of 35.9 μM [124].

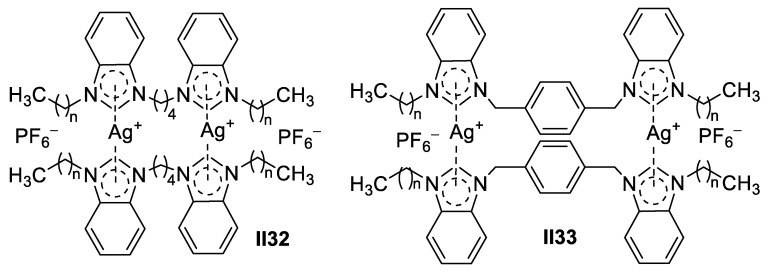



##### Palladium-Based Imidazole Supermolecules as Anticancer Agents

Due to the analogous complex formation of Pt (II) and Pd (II), much effort has been devoted to developing palladium derivatives as anticancer supramolecular drugs [125]. However, compared to platinum anticancer drugs, most palladium supermolecules are less toxic to cancer cells, which is due to the instability of the palladium supermolecules, leading to the hydrolysis of the palladium supermolecules before they reach their pharmacological action target. On this basis, utilizing imidazole as a strong complexation ligand in Pd (II) complexes has attracted widespread attention for developing potential anticancer drugs.

Two molecules of thiocarbonyl imidazoles together with one molecule of PdCl_2_ afforded trimolecular supermolecule **II34**, with antiproliferative activity against leukemia HL-60 cell lines that was equal to that of cisplatin. More importantly, it was two times more active than cisplatin against HeLa cell lines [126].

Two equivalent naphthalene methylimidazoles and one equivalent PdCl_2_ formed Pd (II) complex **II35**. Compared to cisplatin, it was found that the Pd (II) naphazoline-based complex **II35** exhibited remarkable antiproliferative activity against MCF-7, which was more than that of the HeLa cell lines. Palladium (II) naphazoline-based complex **II35** possessed a strong antitumor activity against MCF-7 cells, with an IC_50_ value of 3.73 μM, in comparison to cisplatin, which had an IC_50_ value of 26.66 μM. This finding demonstrated the great potential of the Pd (II) complex as an anticancer drug [127].

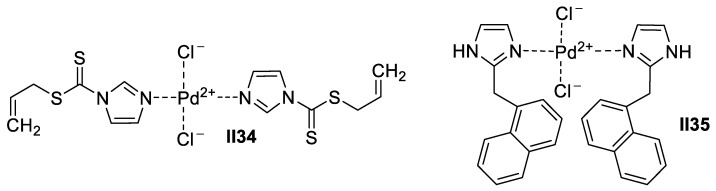



Sulfur atoms were likely to form stable supermolecules with metal ions. Complex **II36**, formed from 2-amino methyl benzimidazole and a cysteine-containing sulfhydryl group in PtCl_2_, inhibited laryngeal carcinoma similar to doxorubicin, with an IC_50_ value of 0.6 μg/mL. If methionine supplanted cysteine, the anticancer activity would be found to be only half as potent as that of doxorubicin, but its activity toward rectal cancer HCT116 lines was comparable to that of doxorubicin (IC_50_ = 0.74 μg/mL). In the anticancer mechanism study, though both target DNA to exert efficacy, compound **II36** was less nephrotoxic than cisplatin because the amino acid ligands were quite difficult to be replaced by proteins containing a sulfhydryl group [128].

Two sets of macrocyclic, bio-inspired, non-heme ligands are utilized for the synthesis of Ni (II), Pd (II), and Pt (II) complexes. These ligands consisted of a 16-atom macrocycle, formed by four methylene-bridged benzimidazole moieties. The Pd (II)-based complex **II37** showed the highest activity in A2780cisR cells and HeLa cells, offering 100 times and 7 times higher activity than cisplatin, respectively. The Pd (II)-based complexes showed remarkable antiproliferative properties in specific cancer cell lines, and complex **II37** offered luminescence properties without an additional marker, enabling future distribution studies of these substances in cells [129].

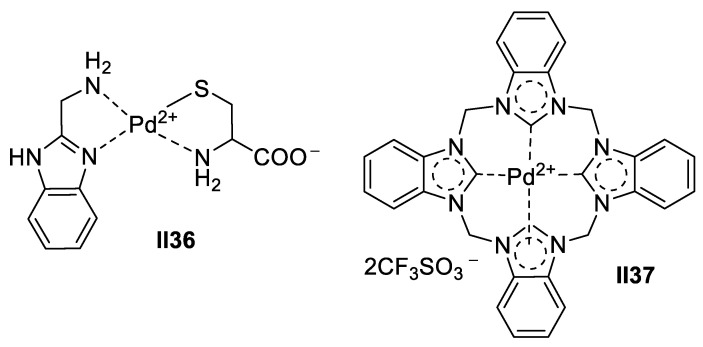



#### 2.1.2. Transitional Metal-Based Imidazole Supermolecules as Anticancer Agents

Following the great achievements of and further studies on noble metals, plenty of research has naturally extended to transition metal complexes [130,131]. Due to the variety of coordination numbers and rich forms of transition metals such as ruthenium, copper, iron, and rhenium, many efforts have endeavored to exploit transition metal supermolecules as anticancer agents in recent years. Transition metal complexes are reported to afford a wide range of coordination modes, with diverse coordination numbers and geometries, various redox states, and the tunability of characteristics [132,133].

##### Ruthenium-Based Imidazole Supermolecules as Anticancer Agents

Ruthenium and platinum are all affiliated with platinum-group metals in the periodic table and share similar chemical properties. As a consequence, ruthenium supramolecular complexes have also been widely put into consideration in anticancer pharmaceutical research. Owning low cytotoxicity with the abilities to resist metastasis and conquer cisplatin tolerance, ruthenium supermolecules are expected to be latent anticancer candidates. Particularly, imidazole-based supermolecules have made significant progress, with imidazole-Ru (II) NAMI-A and Ru-involved pyrazole supermolecule KP1019 already undergoing clinical trials [134].

Trimolecular supermolecule **II38**, formed by Ru (II) together with a bidentate ligand of imidazole and carbazole, could overcome cross resistance toward cisplatin. By reacting with key protein B1 in the process of proliferation, supermolecule **II38** evidently suppressed the growth of liver cancer HepG2 cells, cervical carcinoma HeLa cell lines, lung cancer A549 cells, cisplatin-tolerant lung cancer A549cisR cell lines, and nasopharyngeal carcinoma CNE-2. More importantly, its activity against cisplatin-resistant lung cancer A549cisR was 40-fold more potent than that of cisplatin, while it showed quite a low toxicity in stark contrast to normal lung cells HLF. Benzene did not work as well as *p*-Cymene regarding 25 terms of antitumor activity. The high antitumor activity might be in close relation with its tendency to form hydrogen bonds with target sites. In addition, the dehydration of the Ru-Cl bond in the Ru (II)-arene complex was also essential for the supramolecular combination and activation with DNA and proteins [135].

The imidazole-mesalamine Schiff base Ru (II) complexes **II39a–b** were effective in inhibiting the growth of 3D colon cancer stem cell spheroids and bulk colon cancer cells at lower doses than salicycin and oxaliplatin, which were more resistant to the efflux of different transporters than oxaliplatin and did not enhance the expression of stem-regulatory genes (SOX2, KLF4, and OCT4). Different from salinomycin, complexes **II39a–b** inhibited colon cancer stem cells (CSCs) without enhancing the expression of HES-1, which provided a basis for further understanding its mechanism in future complex research. These results broadened the research field of Ru (II) complexes and opened up a new way to study Ru (II) complexes as anti-CSC agents [136].

Butyl-substituted imidazole ruthenium (II) complex **II40** displayed an exceedingly distinct anticancer activity against A549 cancer cells (IC_50_ = 14.36 mm), with approximately 1.5-fold better activity than the clinical platinum drug cisplatin (IC_50_ = 21.30 mm) in A549 cancer cells. Mechanism studies revealed that compound **II40** mainly mediated cell death through cell stress, including cell cycle arrest, the induction of apoptosis, an increase in the intracellular ROS level, and mitochondrial membrane potential (MMP) depolarization [137].

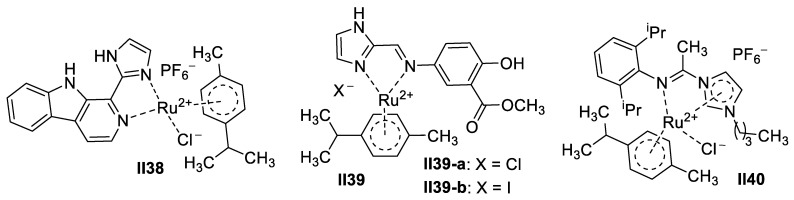



Biphenyl and imidazole phosphorus Ru (II) complex **II41** exerted distinct cytotoxicity toward ovary cancer and rectal cancer, in cooperation with the NH structure on the imidazole ring to facilitate its interaction with DNA. As was revealed in the structure-activity relationship, the introduction of a methyl group in the imidazole ring reduced the antitumor activity of the supramolecular complex, probably for its deformation of the originally planar structure of imidazole [138].

The limited water solubility of ruthenium complexes may be a major limitation for their potential clinical application against cancer. Ruthenium (II) imidazole complexes **II42a–e** containing water-soluble phosphine showed high to moderate cytotoxicity to A2870, MDAMB231, and HT29 cells, and the A2780 cells were more sensitive to the compounds. All the complexes were highly soluble in water [139]. 

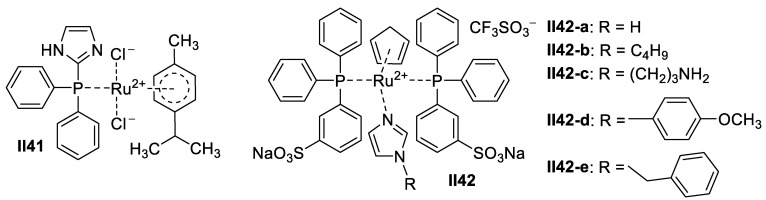



Groups of naphthalimide can be utilized as DNA intercalators in the anticancer field. Complexes **II43a–d**, formed by Ru (II) and naphthalene amide imidazole, were found to selectively have moderate inhibitory activity against ovarian cancer A2780 cells and cisplatin-tolerant A2780cisR cell lines, with IC_50_ values all below 10 μM, exhibiting little toxicity toward normal cells. The structure-activity relationship suggested that the volume of the aryl substituent was a determinant of the antitumor activity [140]. **II43-a** and **II43-b**, substituted by bulkier aryls, were more efficient toward cancerous cells than complexes **II43-c** and **II43-d**. In addition, the antitumor activities of **II43-a** and **II43-b** paralleled those of **II43-c** and **II43-d**, respectively. This result clarified that the substituents on naphthalene amide had a negligible effect on the total activity of molecules. More importantly, it was found in a further study that central ruthenium ions had a preference for combining proteins rather than interacting with DNA, whereas interaction with DNA effectively worked in the presence of a naphthalene amide group. In conclusion, the synergistic effect of two mechanisms could equip such a kind of mixed structure with benign antitumor bioactivity [141].

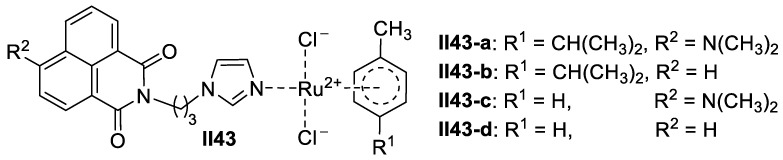



Diimidazole Ru (II) complexes exhibited outstanding properties in DNA interaction for their diverse chemical structure and low biotoxicity. The formation of G-quadruplex prevented telomerase from distinguishing telomere DNA primers to inhibit the expression of telomerase activity. Ruthenium (II) complex **II44**, coordinated with diimidazole and polypyridine, could transform telomere into G-quadruplex and, thereby, effectively inhibit the proliferation of HeLa cervical, A549 lung, and HepG2 liver cancer cell lines, providing a broad spectrum of anticancer activity [142,143].

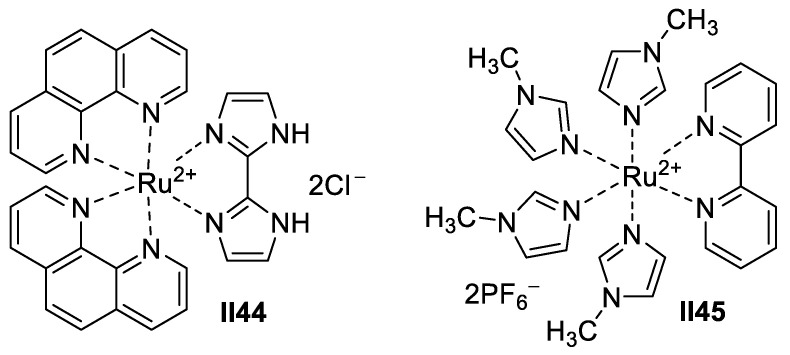



Four-methylimidazole-substituted bipyridine ruthenium complex **II45** with a higher logP_o/w_ exhibited faster cellular uptake rate but lower anticancer activity than bipyridine ruthenium complex with no-methyl-substitution. Complex **II45** predominantly accumulated in the mitochondria and cytoplasm and induced G0/G1 cell cycle arrest, whereas the more hydrophilic no-methyl-substitution complex tended to localize and accumulate in the cell nucleus and mitochondria. Further mechanism studies indicated that the no-methyl-substitution complex caused cell cycle arrest at the S phase by regulating cell cycle-related proteins and induced apoptosis in A549 cells through DNA damage, cellular ROS accumulation, activation of the caspase pathway, and mitochondrial dysfunction [144].

Many studies revealed that the combination of different metals with the same molecule to form complexes can induce additive or even synergistic effects. Due to the existence of two different metal centers, heteronuclear Pt (IV)−Ru (II) complex **II46** possessed both cytotoxicity and antimetastatic properties, exhibiting benign anticancer activity and prominent cancer cell line selectivity in vitro. Complex **II46** showed superior anticancer activity against cisplatin-resistant A2780cisR cells, with an IC_50_ value 6.9 ± 4.0 μM, compared to cisplatin (IC_50_ = 9.7 ± 1.0 μM). An in vivo toxicity assessment using zebrafish embryos indicated that complex **II46** showed lower toxicity than cisplatin [145]. In addition, Ru (II) metallic macrocycle complex **II47** containing *p*-cymene and imidazole ligands gave comparable activity (IC_50_ = 16.7 μM) to that of anticancer drug cisplatin (IC_50_ = 15.8 μM) toward the HeLa cell line [146].

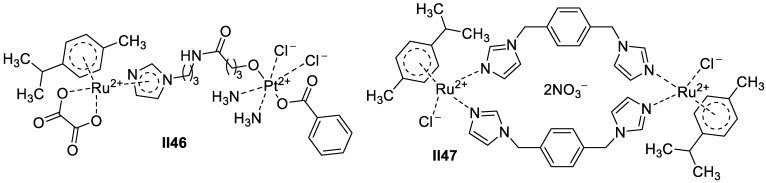



Glycyrrhizin acid is an active triterpenoid metabolite of Glycyrrhiza glabra. A Ru (II) arene complex **II48** with an imidazole ligand conjugated to 18β-glycyrrhizin acid showed only moderate activity against HeLa (cervical), MCF-7 (breast), and A2780 (ovarian) cancer cells [147].

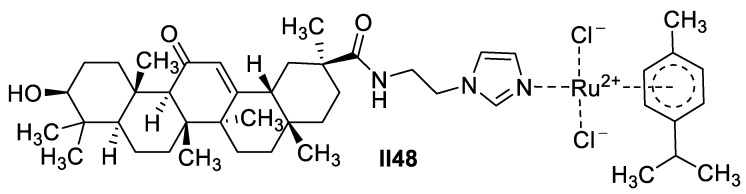



Ruthenium supermolecules **II49a–c**, with fused benzimidazole and polypyridine as chelators, were found to possess wide anticancer activity. By producing active oxygen, the complexes destroyed the mitochondrial membrane and afterward induced apoptosis in HeLa cervical, A549 lung, and HepG2 liver cell lines. A further study demonstrated that the bulkier the substituents on polypyridine are, the more active the supramolecular complexes are, exerting in the order of **II49-c** > **II49-b** ≈ cisplatin > **II49-a**. Among those cell lines treated by **II49-c** above, HeLa cervical cancer cell lines were found to be the most sensitive with the highest uptake level, and, meanwhile, no adverse effect was detected toward normal LO2 cells. In contrast, cisplatin had an indiscriminate effect on both cancer cell lines and normal LO2 cells. Ruthenium complexes **II50a–b** could produce superoxide, causing mitochondrial dysfunction that killed the melanoma of human skin A375 cells, cervical HeLa cells, MCF-7 breast, PC-3 prostate, Neuro-2a brain cancer cells, etc. Methylated benzimidazole derivative **II50-b** was over 10 times more active than Ru (II) supermolecule NAMI-A but was far safer toward normal Hs68 cells (IC_50_ > 400 μM). If the methyl group on benzimidazole was removed, albeit to a reduction in anticancer activity of two times, supramolecular complex **II50-a** hardly posed a threat to normal cells [148].

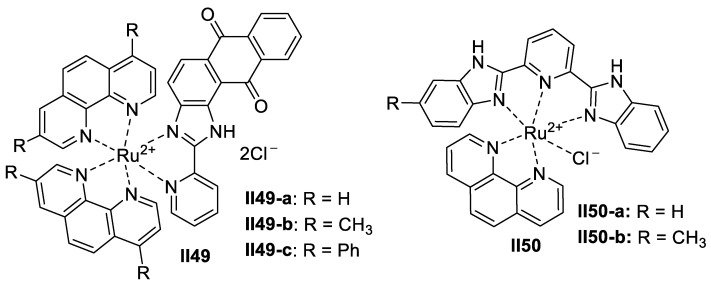



Fused-imidazole ruthenium (II) complex **II51** could be obtained with an excellent yield of 90.7%, which inhibited the growth of human lung adenocarcinoma A549 cells, with an IC_50_ value of 16.59 μM. The spectrum of DNA-binding behavior in vitro confirmed that complex **II51** could selectively bind and stabilize *bcl*-2G-quadruplex DNA to induce the apoptosis of A549 cells. Therefore, complex **II51** had remarkable *bcl*-2G-quadruplex DNA binding and stabilizing activity, which exhibited a potential application value in cancer chemotherapy [149].

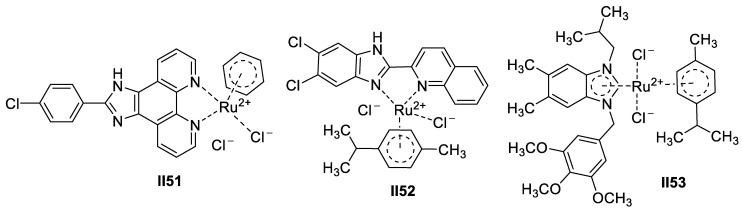



Quinoline-substituted benzimidazole could coordinate with the ruthenium (II) ion to form a sandwich supermolecule **II52** by a simple one-pot method under a continuous ultrasound, which was significantly more effective in inhibiting Caco-2 and HeLa cell lines (IC_50_ values = 6.16 ± 0.8 and 3.52 ± 1.7 μM, respectively) compared to cisplatin (IC_50_ values = 17.9 ± 1.2 and 13.26 ± 0.6 μM, respectively). It even showed significant cytotoxicity to the more aggressive HT-29 colorectal cancer cell line, which could induce oxidative stress or arrest the cell cycle [150].

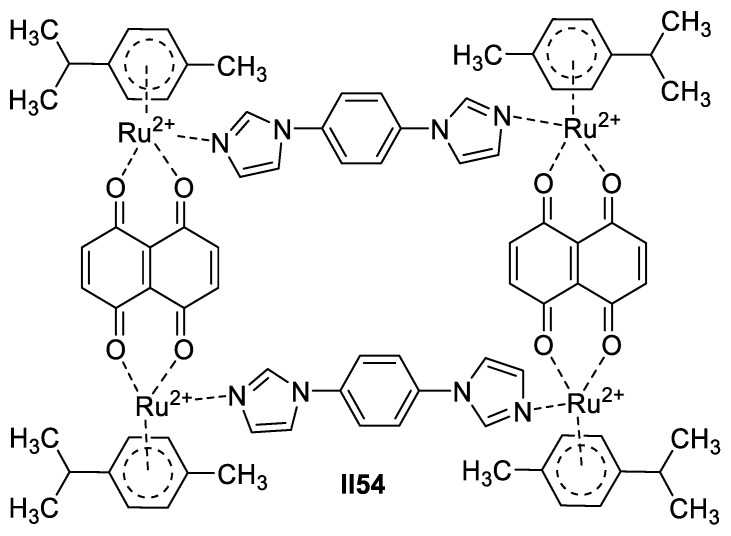



The *p*-cymene benzimidazole Ru (II) complex **II53** was formed by transmetalation via the corresponding silver complex. The cytotoxicity of compound **II53** was much stronger in MCF-7, with an IC_50_ value of 0.6 μg/mL, compared to activity against MDA-MB-231 cells, with an IC_50_ value of 3.1 μg/mL [151].

The ruthenium (II) cyclic derivative **II54**, formed by four-imidazole and quinone with multimodal chemo phototherapy properties, showed higher anticancer activity and better cancer cell selectivity than cisplatin. Ruthenium (II) complex **II54** showed significant cytotoxicity to A549 cells and cisplatin-resistant A549 cells, even if a hypoxic environment did not prevent it from killing tumor cells. Ruthenium (II) metal complex **II54** could efficiently activate caspase 3/7 and cause apoptosis. These results opened up new possibilities for the application of Ru (II) metal rings in biomedicine [152].

Benzimidazole-bridged ruthenium rectangle complex **II55** was obtained from the corresponding double-nuclear arene ruthenium compounds via the reaction of dibenzimidazole and AgCF_3_SO_3_, which showed the highest antiproliferative effect against the human breast cancer cell line (MDA-231-MB) among the prepared compounds during 24 and 48 h administration. Moreover, complex **II55** exhibited very good cancer cell selectivity and very low micromolar cytotoxicity [153].

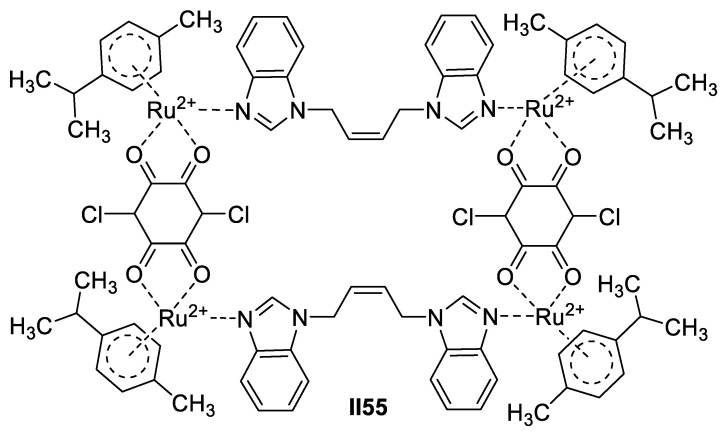



##### Copper-Based Imidazole Supermolecules as Anticancer Agents

Copper ions, which are highly engaged in life activities and constitute plenty of metal-loaded proteins [154], are able to form relatively stable supermolecules with imidazole compounds. Furthermore, copper complexes show encouraging perspectives because endogenous copper ions favor less toxicity toward normal cells compared to cisplatin [155]. Cancerous cells can selectively intake more copper ions than normal cells via cell membrane permeation [156,157]. Thus, this is of great significance for the development of supramolecular anticancer drugs with imidazole-based copper complexes.

Imidazole-substituted terpyridine and Cu (II) ion could form the stable complex **II56**, which acted on DNA and suppressed the 50% growth of lung cancer A549 cells at the value of 0.81 μM. Several consequent studies showed that the conversion of nitrite ions into perchlorate markedly improved anticancer activity, and the growth inhibition of 50% (GI_50_) value was 0.64 μM. It was revealed that the planar imidazole ring enabled the complex to embed into the DNA scaffold and form a hydrogen bond with base pairs on the DNA using nitrogen atoms on the rings to stabilize its combination with the DNA [158]. Broad-spectrum suppression against multiplication was also observed in trials of structurally similar **II57**, with GI_50_ values that were all less than 10 μM against glioma U373-MG cells, ovary cancer A2780 cells, leukemia K562 cells, rectal cancer HCT15 cells, breast cancer MCT-7 cells, prostate cancer PC-3 cells, lung cancer Hop62 cells, etc. Complex **II57** inhibited the activities of topoisomerases I to achieve the antitumor goal [159].

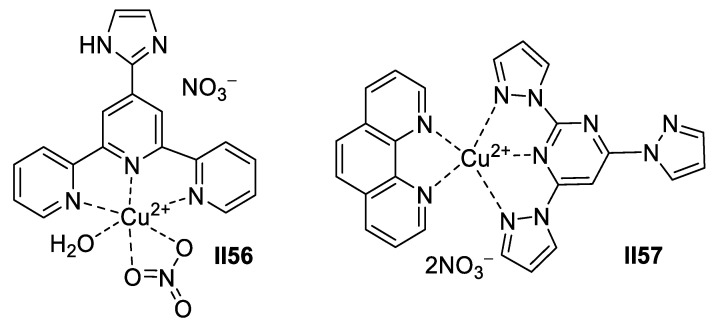



Supermolecule **II58**, composed of a Cu (II) ion, imidazole, and a Schiff base of salicylaldehyde and thiosemicarbazide, with IC_50_ values all below 0.5 μM, inhibited breast cancer MDA-MB-231 and MCF-7 cell lines more strongly than the breast clinical drug tamoxifen. In contrast, the usage of benzimidazole to replace imidazole for complex **II58** lowered the antitumor activity, probably due to the poor water solubility that decreased the bioavailability [160]. Complex **II59**, formed by an ethyl-bridged Schiff base and benzimidazole, could cleave the DNA of breast cancer HBL-100 cell lines, thus, playing a better role in attacking cancerous cells than the reference drug cisplatin. More interestingly, complex **II59** demonstrated a non-toxic activity toward normal lymphatic cells [161].

Hydrazone imidazole complex **II60**, formed by nonsteroidal anti-inflammatory drug (NSAID) ibuprofen and a Cu (II) ion, possessed substantially lower IC_50_ value (3.38 μM) against MDA-MB-231 in comparison to cisplatin. Comparatively lower activity was observed for the produced hydrazone complex against A549, whereas it displayed extensive anti-proliferative activity against the breast cancer cell line. Interestingly, complex **II60** was found to be almost 15 times more potent than cisplatin, a clinically approved anticancer drug. Such behavior might be attributed to the combined effect of both NSAID and the hydrazone motif that significantly improved the antitumor activity of the conjugates. Therefore, the combination of hydrazone with NSAIDs and its complexation with less toxic metal ions could be considered as an alternative strategy for designing powerful anticancer drugs, which had better activity at lower concentrations than cisplatin [162].

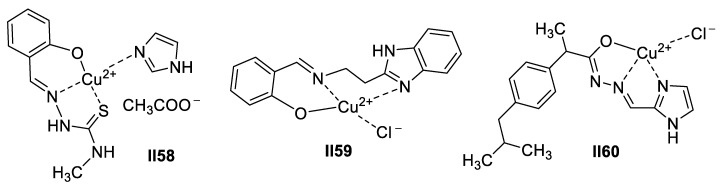



Imidazole copper (II) complex **II61** showed significant anticancer activities. The IC_50_ values of complex **II61** for the human lung cancer A549 cell line (0.59 μM) and the breast cancer MCF-7 cell line (8.88 μM) were low. More importantly, complex **II61** was found to be significantly less toxic to the normal cell line L132 of human lung embryos, which could be obtained from a cell survival assay [163].

Aryl imidazole Cu (II) coordination complex **II62** was found to show a powerful anticancer effect against breast carcinoma (MCF-7 cell line), with an IC_50_ value of 10.5 μg/mL, which was significant and comparable to that of the reference drug vinblastine, with an IC_50_ value of 5.71 μg/mL [164].

Coumarin-based copper (II) complex **II63** avidly bound to CT DNA, compared to cisplatin, via a partial intercalative mode. Compared to cisplatin (57.7 ± 0.9 mM), complex **II63** demonstrated exceptionally excellent cytotoxicity against the cisplatin-resistant A549 lung cancer cell line, with an IC_50_ value of 4.6 ± 0.3 mM [165].

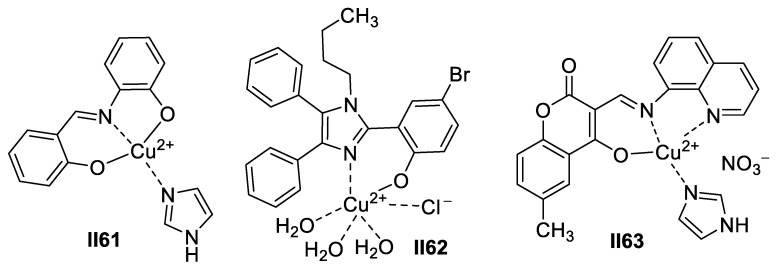



The role of imidazole in bimetallic Cu (II) complex **II64** was explored by molecular docking studies. It was revealed that two nitrogen atoms on the imidazole ring were engaged in connection with TERT protein catalytic subunit via hydrogen bonds with the amino hydrogen of LYS-406 and the hydrogen atom of LYS-406, respectively. Remarkable GI_50_ values against MCF-7 (<10 ± 0.236), K562 (<10 ± 0.078), and A2780 (<10 ± 0.308) carcinoma cell lines were also obtained. Moreover, complex **II64** also efficiently cleaved plasmid pBR322 DNA and the hydrolytic cutting of the DNA duplex [166].

Bipyridine-substituted imidazole copper (II) complex **II65** presented more significant anti-angiogenic activity than the well-known anti-angiogenic agent (±)-thalidomide. It was indicated that the copper (II) complex **II65** had potent antiangiogenic activity and was found to be a promising new metallodrug candidate for the treatment of angiogenesis-based tumor growth [167].

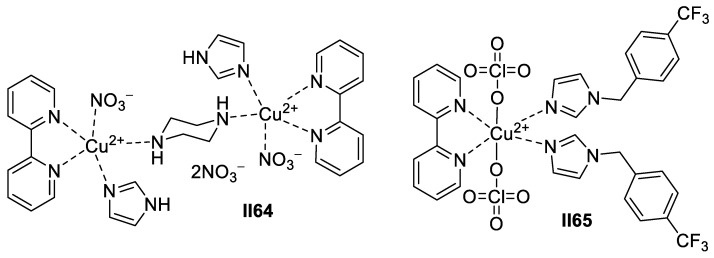



Imidazolium-derived Schiff base thiosemicarbazone with benign water solubility could coordinate with copper (II) to form the corresponding metal supramolecular complexes **II66a–b**, since the ligand acted as a tridentate, and the complex had distorted square planar geometry with a labile Cu-Cl bond. The cell cycle and apoptosis showed that phenyl-substituted thiosemicarbazone complex **II66-a** was an inhibitor of cell cycle progression at the G2 phase and induced apoptosis death in MCF-7 cells after 24 h of treatment in MCF-7 cell lines, and the IC_50_ of phenyl-substituted thiosemicarbazone complex **II66-a** was 35.61 ± 4.38 μg/mL, which was lower than that of Taxol (IC_50_ = 59.21 μg/mL). When the phenyl group was replaced by the methyl group, the activity of complex **II66-b** was reduced [168].

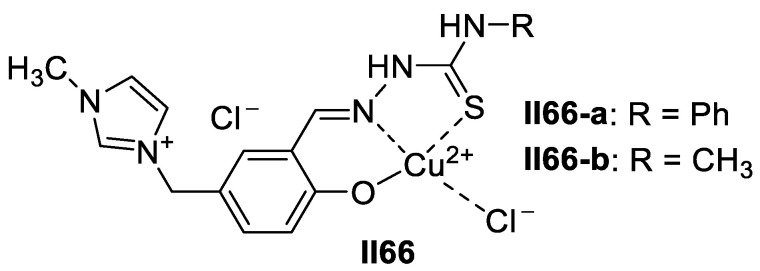



Copper (II) benzimidazole complex **II67** displayed promising cytotoxicity against A549 adenocarcinoma alveolar basal epithelial cells, with an IC_50_ value of 5.5 ± 0.4 μM. The anticancer activity of Cu (II) complex **II67** was stronger than that of either the benzimidazole-derived ligand on its own or the free Cu (II) compound. This might indicate that the ligand plays a role in enabling the copper centers to effectively generate elevated levels of toxic ROS, leading to cell death [169].

Supermolecule **II68** contained amino acid ligands and an assembly of the direct coupling of thiazole and benzimidazole, both at their 2-position. Although the complex behaved less toxically than cisplatin toward cervical HeLa cancer, it performed better than 5-fluorouracil, with an IC_50_ value of 33.17 μM [170].

Benzimidazoles complexed with a Cu (II) or Co (II) ion demonstrated protein kinase inhibitory and anti-inflammatory activities. Based on these activities, copper (II) benzimidazole complex **II69** showed antiproliferative activity against SMMC7721 cells, permeated the cell membrane, and entered the nucleus and mitochondrion. Moreover, complex **II69** was found to interact with DNA and induce apoptosis [171].

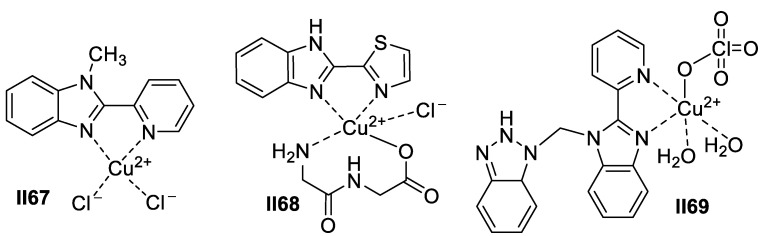



In order to improve the water solubility and reduce the toxic side effects of the metal complexes, some biological metabolites, such as dipeptides, amino acids, and glucose were introduced as ancillary coordination ligands to synthesize ternary metal complexes. A water-soluble Cu (II) complex **II70** bearing a D-gluconic acid ligand exhibited good antibacterial activity against *Staphylococcus aureus*, *Bacillus subtilis*, *Listeria monocytogenes*, and *Escherichia coli*. It also displayed high anticancer activity in vitro toward the tested cancer cells (A549, HeLa, and SGC-7901) and relatively low toxicity to normal liver cells. Further antitumor mechanism studies revealed that Cu (II) complexes could induce apoptosis through DNA binding and ROS-mediated mitochondrial dysfunction pathways. The inhibitory rate of complex **II70** on tumor growth was 50.44%, which was higher than that of cisplatin (40.94%), indicating that the activity of complex **II70** was higher than the activity of cisplatin under the same conditions, and its toxic side effects were significantly less than those of cisplatin, showing good antitumor activity in vivo [172].

Despite numerous targeted therapies, drug resistance and treatment failure are still common in cancer treatment. In this context, researchers evaluated copper (II) imidazole [1,2-a] pyridine as an alternative candidate for the treatment of two common leukemias (represented by HL-60 and K562 cells). A previous study found that imidazole [1,2-a] pyridine copper (II) complexes **II71a–b** were cytotoxic to low leukocyte cell lines, with IC_50_ values between 1.9 and 6 μM. Imidazole [1,2-a] pyridine copper (II) complexes **II71a–b** played a role by reducing the mitochondrial integrity of the two cell lines, leading to the release of apoptosis-promoting proteins into the cytoplasm, which exceeded the inhibitory effect of the apoptosis proteins. This research showed that the mitochondria were an important target of copper imidazole [1,2-a] pyridine complexes and were the center of cytotoxicity and the apoptosis induction of the complexes. These findings further supported the potential research value of imidazole [1,2-a] pyridine copper (II) as a chemotherapy drug in clinical settings [173].

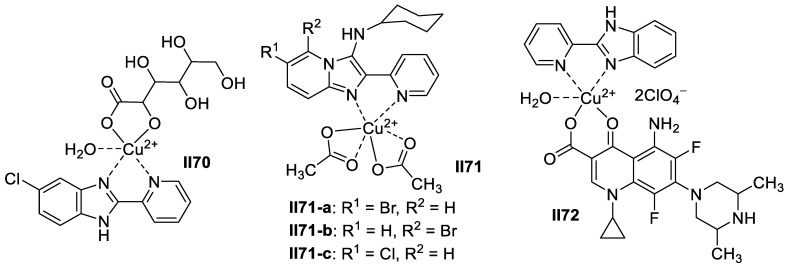



Copper (II) pyridine-substituted benzimidazole sparfloxacin complex **II72** inhibited the growth of cancer cells more efficiently than the corresponding benzothiazole complex, which was consistent with the order of their DNA binding and cleavage abilities. Moreover, complex **II72** showed selectivity for cancer cells, since its toxicity toward normal LO2 cells was significantly lower than that of the tumor cells, indicating that complex **II72** might be a potential antitumor drug. Eca-109 cells were the most sensitive to complex **II72** (IC_50_ = 14.1 ± 0.5 μM) [174].

##### Iridium-Based Imidazole Supermolecules as Anticancer Agents

Iridium is capable of variable oxidation states and kinetic stability in biological systems. Its complexes can exert good anti-proliferative activity in vitro/in vivo and multiple mechanisms of action [175]. Iridium complexes have been extensively investigated as potential anticancer agents in recent years.

Iridium (III) complexes **II73a–c** could be synthesized from biimidazole and 2-phenylpyridine. It was indicated in an anticancer study that the length of the alkyl groups on the imidazole ring could influence the antitumor ability and present in the order of **II73-c** > **II73-b** > **II73-a** > cisplatin, which was consistent with their lipophilicity. Among these cancerous cells, complexes **II73a–c** were detected to be selective to cervical HeLa cancer cell lines. Of all the compounds tested, the best performer was **II73-c**, with an IC_50_ value of 1 μM, which was 10-fold more active than cisplatin and selectively toxic toward cancer cells. Nevertheless, ligands had no activity in all cells, with their IC_50_ values all over 100 μM. Importantly, complexes **II73a–c** were 20–30 times more active than cisplatin toward cisplatin-resistant A549cisR, overcoming the drug tolerance of cisplatin [176].

When it comes to the direct connection between the two alkyl-substitution imidazole rings, cyclometalated Ir (III) complexes were capable of generating singlet oxygen, thus exhibiting photodynamic therapy (PDT) efficacy. Butyl-substituted diimidazole Ir (III) complex **II74** performed appreciably well (PI = 150) toward HeLa cells. Located in the mitochondria, complex **II74** preferentially targeted tumor cells such as HeLa, A549, A549R, and LO2, and a desirable PDT effect was also obtained toward A549R cell lines resistant to cisplatin, with a mean IC_50_ value (around 1.5 μM) far less than that of cisplatin (IC_50_ = 104.3 ± 4.5 μM). The consequence afforded valuable hints for PDT optimization by lipophilicity tuning in the wake of alkyl substitution [177].

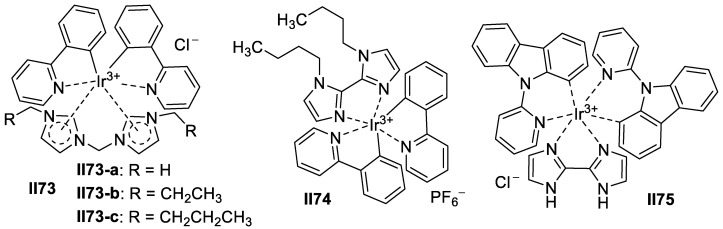



Iridium (III) complexes have attracted much attention for the high tunability in their intrinsic photophysical profiles. For instance, carbazole-based iridium complexes with green emission were in the device for new biological sensing and exerted a DNA-cleaving capability. The prepared Ir (III) compound **II75** could lead to the cleavage of pBR322 DNA, *plasmid pBluescript II KS +* DNA, or pUC19 DNA. Studies of in vitro inhibition toward HeLa demonstrated that complex **II75** (IC_50_ = 9.3 μM), which has a superior DNA/BSA binding capability, should hold the potential for novel antiproliferative application [178].

A half-sandwich Ir (III) benzimidazole compound **II76** showed more potent anticancer activity against A549 cancer cells, with a minimum IC_50_ value of 5.9 ± 0.2 μΜ, which was much lower than that of the reference drug cisplatin (IC_50_ = 21.3 ± 1.7 μΜ). Each C^N chelating ligand formed a six-membered ring with a metal center Ir (III). There were benzene rings connected at different positions in the benzimidazole ring, which could effectively enhance the anticancer potency. Compound **II76** could bind with bovine serum albumin (BSA) by static quenching to catalyze the oxidation of nicotinamide adenine dinucleotide (NADH) and increase the level of ROS. At the same time, compound **II76** inhibited the A549 cell cycle and significantly affected the mitochondrial membrane potential [179].

Two mononuclear cyclometalated iridium (III) complexes **II77a-b** were designed and developed incorporated imidazole-based cyclometalated ligands with short and long alkyl chains. The in vitro growth inhibition assay of complex **II77-a** with a shorter alkyl chain displayed higher anticancer activity (IC_50_ < 0.5 μM) with moderate cancer cell selectivity (three times) against MCF-7 cancer cells compared to that of the longer alkyl chain complexes **II77-b** (IC_50_ < 30 μM). The present set of complexes demonstrated that the imaging property or anticancer activity could be tuned by a simple variation of the alkyl chain length [180].

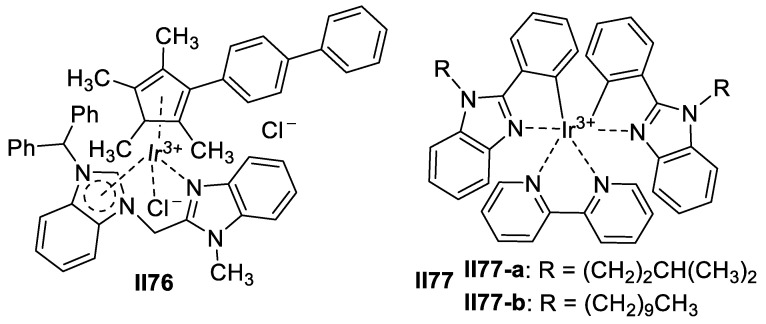



##### Iron-Based Imidazole Supermolecules as Anticancer Agents

Iron is an essential trace element necessary in the human body, which has rich physiologic functions and a strong coordination capacity [181]. Supermolecules can be produced from the coordination of iron ions and electron-rich imidazole compounds and then equipped with latent antitumor activity. More specifically, iron as a replacement for the central ions in conventional antitumor complexes has attracted extensive attention in the field of anticancer.

The ferrocene analogue of tamoxifen for breast cancer showed good toxicity in cancer cells, and their bioavailability in vivo was significantly improved after they were combined with cyclodextrin and nano capsules to form organic supermolecules [182]. Benzylimidazole complex **II78-a** rivaled cisplatin toward ovarian cancer A2780 cell lines (IC_50_ = 2.00 μM). The introduction of long alkyl chains or hydrophilic oxhydryl could give complexes **II78-b** and **II78-c**, with IC_50_ values that were 0.22 and 0.43 μM, respectively, activities toward the A2780 cancerous cells of both products, which were ameliorated. For example, the activity against breast cancer MCF-7 cell lines was 30 times more active, with low IC_50_ values down to 0.8 μM, than that of cisplatin. Moreover, supramolecular complexes **II78-a** and **II78-c** also outperformed cisplatin toward cervical HeLa cancer cell lines. This research effectively spurred further clinical investigations [183].

Two metal mononuclear complexes **II79a–b** (M = Zn, Fe) were produced through the reaction of 2-((1*H*-tetrazol-1-yl)methylene)-1*H*-imidazole-4,5-dicarboxylic acid and ZnSO_4_·7H_2_O or FeSO_4_·7H_2_O. It was found that the anticancer activity of both complexes against Eca-109 was better than that of the corresponding ligand [184].

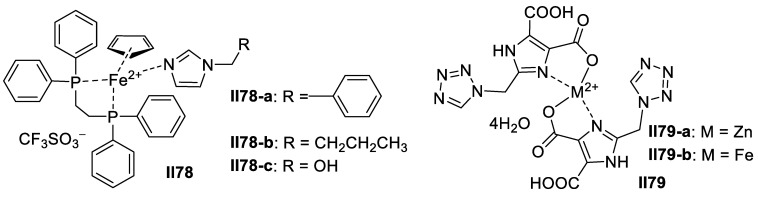



The study found that there was good efficiency of the 2-aminomethylbenzimidazole complexes against cancer cells, in particular cytotoxic activity in the MCF-7 cell line, colon carcinoma (HCT116), larynx carcinoma (HEP2), hepatocellular carcinoma (HepG2), and neuroblastoma (SHSY5Y). Three different metal complexes including Zr (II), Cd (II), and Fe (III) were prepared from Schiff bases that were synthesized by the condensation of 2-aminomethylbenzimidazole and 2-hydroxybenzaldehyde. A biological assessment revealed that Fe (III) complex **II80** demonstrated strong toxicity toward Hep G2 and HCT cells but moderate activity against MCF-7 [185].

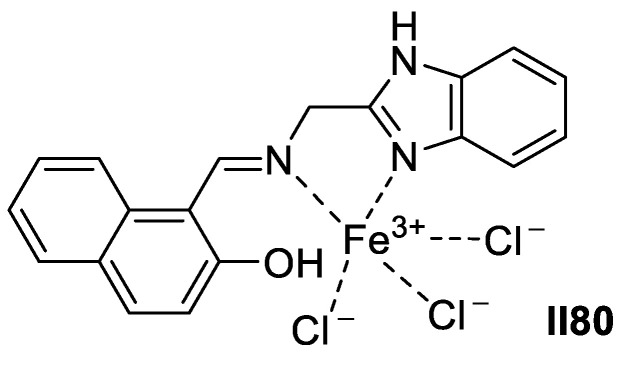



##### Rhenium-Based Imidazole Supermolecules as Anticancer Agents

Rhenium plays a significant role in medicine imaging, contributing a lot to discovering, diagnosing, and treating cancer [186]. At the same time, there are increasingly active projects of rhenium-based imidazole complexes in the realm of cancer prevention.

Rhenium’s antiproliferative activity against a panel of pancreatic carcinoma cell lines (HPAF-II, ASPC1, and CFPAC) demonstrated that tricarbonyl rhenium-derived compound **II81** outperformed the indomethacin analogues complexes on the whole. The comparable antitumor activity of compound **II81** was also observed with respect to carboplatin. A possible anticancer mechanism was put forward: through the inhibition of the phosphorylation of the Aurora-A kinase, the cell cycle arrest at the G2/M phase was induced when treated with rhenium compounds. According to structure-activity relationship research, the stability of the ancillary ligands in rhenium compounds had an effect on the antitumor activities [187].

Fluorine-involved imidazole Re (I) complex **II82-a** and trifluoromethyl-involved Re (I) complex **II82-b** were liable to give singlet oxygen, which contributed to their taking an effect on cancer cells. Furthermore, the concentration for 50% of maximal effect (EC_50_) values of complex **II82-a** against HeLa cell lines in the light and dark differ from each other, since the activity under irradiation was 30 times more active than in the dark [188].

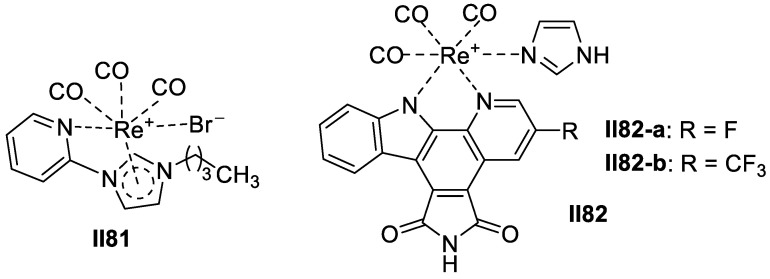



##### Vanadium-Based Imidazole Supermolecules as Anticancer Agents

The large medicinal potential of metal supramolecular complexes has attracted an increasing amount of attention toward vanadium-based supermolecules in recent years. The good anticancer activity and low toxicity of vanadium complexes implies a potential promise in treating cancer diseases [189].

Water-soluble mononuclear dipicolinic acid-imidazole-based oxidovanadium (IV) complex **II83** could help to develop anticancer compounds with improved efficacy and fewer side effects; this demonstrated dose-dependent cytotoxicity in human breast cancer cells, with an almost equal anticancer potency as that of cisplatin, and was an anticancer agent with high potential for selectively against human breast cancer cells. In contrast to the popular platinum-based drug cisplatin, complex **II83** showed little toxicity to normal cells in vitro and had no serious toxicity to vital organs such as the liver and kidney when checked in vivo as well. Complex **II83**, containing imidazole substitution, showed significant anticancer activity against the human hepatic carcinoma cell line Hep3B. These results indicated that the presence of an imidazole ring and the substitution of the imidazole core by the allyl group played an important role in regulating cytotoxic activity [190,191].

Triazole-bridged benzimidazole Schiff base and naphthalimide could form metallic supramolecular complex **II84** with curcumin and V (IV). During the exposure to irradiation, singlet molecular oxygen was obtained, with a tight combination of DNA and cleavage capability, and, therefore, complex **II84** demonstrated benign activity toward HaCaT cells and MCF-7 cell lines, with their IC_50_ values approaching 6.3 and 5.4 mM, respectively. This result suggested that the introduction of a naphthalimide fragment could strengthen the anticancer activity of complex **II84** [192].

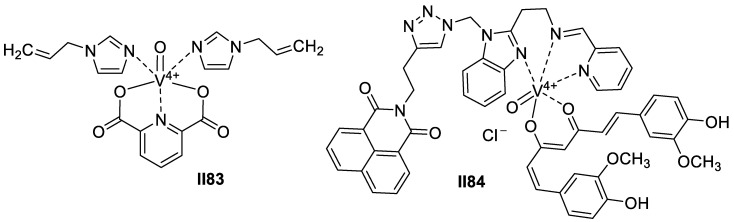



##### Other Transitional Metal-Based Imidazole Supermolecules as Anticancer Agents

Apart from those transition metals mentioned above, other transition metals such as manganese and cobalt complexes are also reported to show medicinal potential.

Carbon monoxide (CO) is an important signaling molecule that plays a significant role in the pathogenesis of cancer. Cancer cells exposed to several stress factors (hypoxia, reactive oxygen species, cisplatin, and oxidative stress) and Heme oxygenase 1 (HO-1) display a cytoprotective role against oxidative stress and inhibit apoptosis, metastases, angiogenesis, and cell proliferation processes. Therefore, metal-containing CO-releasing molecules (CORMs) have been designed as an effective cancer treatment strategy. A CORM is responsible for issuing and controlling the amount of CO entering cells and tissues. Incorporating imidazole analogues as ligands, manganese containing CO-releasing molecule **II85** was devised with the aim of inducing an antiproliferative process by setting free CO when irradiated. It was indicated that this compound inhibited cell proliferation and exhibited a cytotoxic effect on breast cancer cells in in vitro experiments. In the assay of inhibition toward MCF-7, complex **II85** with imidazole side chains acted in stark contrast, probably due to the steric hindrance of an extra methyl substituent on the imidazole ring, which hampered the release of CO [193].

An azo-functionalized Schiff base imidazole thione Co (II) complex **II86** (IC_50_ = 0.90 ± 0.05 μg/mL) was a potent and selective (SI = 90.56) anti-breast cancer agent. The flow-cytometric study revealed that complex **II86** dramatically enhanced the proportion of MCF-7 cells in the sub-G1 phase while decreasing the proportion of cells in the S phase [194].

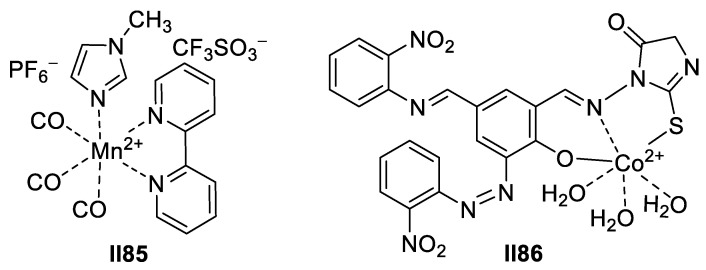



#### 2.1.3. Other Metal-Based Imidazole Supermolecules as Anticancer Agents

The great anticancer potentials of numerous noble metals and transition metal compounds have inspired a lot of exploration into the possibilities for other metals to act as anticarcinogens.

Imidazole-bridged binuclear (Sn (VI) and Cu (II)) supermolecule **II87** could bind to DNA through its strong electrostatic interaction and then break the DNA strand of HCC Huh7 in the form of the free hydroxyl group to achieve an anticancer purpose. Moreover, supermolecule **II87** could bind well with topoisomerase I. Trinuclear organic Sn (IV) demonstrated a higher activity in comparison to that of its binuclear and mononuclear counterparts. This result was mainly related to their binding capabilities to proteins [195].

Lanthanide complexes were synthesized from a Schiff base that was obtained by the reaction of 2-acetylferrocene and L-histidine. The Er (III) and Yb (III) complexes **II88a–b** showed striking antimicrobial and anticancer activities, and these complexes may be promising anticancer drugs for colon and breast cancers as well as antibiotic drugs for certain bacterial species. It was demonstrated that the histidine-derived Schiff base metal complexes showed effective anticancer properties against HCT116 and MCF-7 cancer cells [196].

Cisplatin and its analogues are one of the most widely used anticancer drugs. However, toxicity and drug resistance have limited their use, so gallium compounds have emerged as alternatives due to their significant antitumor activity and low toxicity. It was revealed that the 4-nitrophenol-derived imidazole complex **II89** displayed selectivity and anticancer activity against A549. The IC_50_ of complex **II89** (94.12 ± 4.62) was lower than that of cisplatin (135.10 ± 6.50), which was used as a reference metal drug under the same experimental conditions [197].

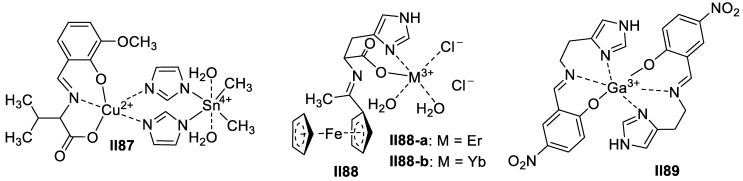



### 2.2. Imidazole-Based Supermolecules as Antibacterial Agents

Antimicrobial agents are widely used in clinical settings to protect human health [17,198]. To date, great scores of various antibiotics have been developed and marketed, such as *β*-lactams [199], tetracyclines [200], aminoglycosides [201], macrolides [202,203], etc. Numerous efforts have been directed toward artificial new antimicrobial candidates such as naphthalimides [204,205,206,207], sulfonamides [208,209], quinolones [50,210,211], coumarins [212,213,214], imidazoles [215], thiazole [216], oxazolidinones [217,218], etc. Nevertheless, the emergence of some new superbugs, due to the extensive use or even abuse of antimicrobial drugs, has greatly weakened the resistance of current drugs in recent years [219,220,221]. Therefore, the innovation of more potent antimicrobial agents is an urgent need globally [222,223,224]. In explorations of the relevant development fields [225,226], imidazoles [227] and benzimidazoles [228] were among the extra-active topics of the endeavored studies against microorganisms for their multitargeting properties, capacities to overcome resistance, and strengthened antimicrobial activities [229,230,231].

#### 2.2.1. Imidazole-Based Supermolecules as Antibacterial Agents

The development of antibacterial agents for the treatment of infection is one of the most well-known achievements worldwide [232]. Imidazole with a five-membered heterocycle is widely used as the essential building block for the development of pharmaceuticals such as antimicrobial metronidazole, tinidazole, ornidazole, etc. [233,234,235]. With regard to the biologically available supermolecules, using imidazole-based supermolecules as antimicrobial agents has attracted wide attention. 

Silver (I) double-imidazole compound **II90-a** restrained the growth of *Escherichia coli* and *Pseudomonas* by destroying the bacterial cell wall with a MIC value of around 4 μg/mL. A structure-activity relationship study showed that compound **II90-b**, which was obtained by replacing the Ag (I) ion with an Au (I) ion, exhibited better antibacterial activity than compound **II90-a**, and the inhibitory activity against the above two types of bacteria was increased by two and four times, respectively. Utilizing the ligands in **II90a–b** as the basic framework in binuclear macrocyclic compounds **II91a–b**, a notable decrease was found compared to **II90a–b**, giving MIC values that exceeded 100 μg/mL toward *Staphylococcus* and *Pseudomonas aeruginosa* [236].

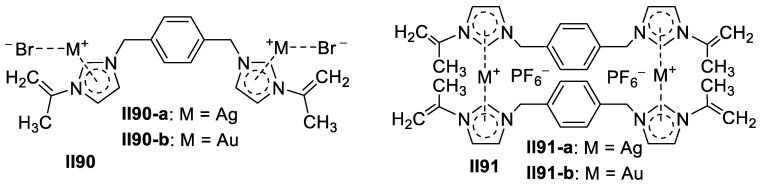



Lots of clinical antibacterial and anticarcinogens maintain their activity against bacteria when coordinated with imidazoles and metallic ions. Tioconazole is a kind of active azole-based component in drug preparations for many fungal or bacterial infections, by restricting enzyme synthesis in microorganisms. Monodentate imidazole together with the hydrochloride of cations (Cu (II), Co (II), Ni (II), and Zn (II)) could generate complexes **II92a–d**, which generally behaved better toward Gram-positive bacteria than Gram-negative bacteria [237].

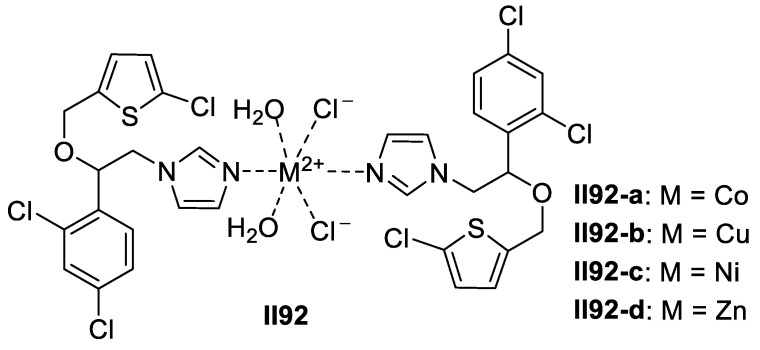



Fluorouracil is categorized as an anti-metabolite of high efficiency, which is liable to form complexes that can exhibit antibacterial activities [238]. Histidine is the major component amino acid of functional proteins. Histidine’s imidazole ring, capable of coordinating with copper ions, enables it to join in the transportation of copper elements. Supermolecule **I93**, with 5-fluorouracil appended to histidine, demonstrated inhibitory activity against *Bacillus subtilis*, *Staphylococcus saprophyticus*, *Staphylococcus aureus*, and *Escherichia coli*, and Cu (II) compound **II93-a** was the most sensitive. The activities toward *Bacillus subtilis*, *Staphylococcus*, and *Escherichia coli* were far more potent than those of 5-fluorouracil, especially in treatment against *Staphylococcus saprophyticus*, with a corresponding MIC value of 2.9 μg/mL. Supermolecules of Ni (II) **II93-c** and Zn (II) **II93-b** gave MIC values ranging from 6 to 8 μg/mL against *Staphylococcus saprophyticus* [239]. Additionally, a similar inhibitory effect was found when the amino acid ligand in **II93a–c** was replaced by simple imidazole compounds **II94a–c**. Copper (II) supermolecule **II94-a** topped the list of activity, and its bactericidal activity against *Bacillus subtilis* was stronger than that of 5-fluorouracil [240].

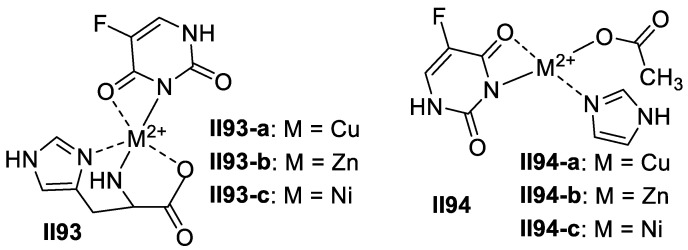



With the low toxicity of silver ions to the human body and the better effect of silver (I) complexes compared to that of commonly used antibiotics, the study of silver complexes has always been a hot spot of concern. Silver (I) supermolecules are also normal in the antibacterial area. For example, mononuclear supermolecule **II95** containing *N*-benzyl imidazole was cytotoxic to the proliferation of *Staphylococcus* and *Escherichia coli*. In contrast, the introduction of a methyl or ester group at the 4-position of the phenyl ring in supermolecule **II95** resulted in nearly no antimicrobial activity [241,242].

Multidentate carboxylate ligands containing nitrogen and oxygen atoms are of great potential in imidazole-based medication upon complexation with Ag (I), given the d^10^ shell configuration and the tendency for weak Ag. An antimicrobial test indicated that compound **II96**, based on propyl-substituted imidazole-4,5-dicarboxylic acid with a MIC_100_ of 0.19 mM, was active against *Escherichia coli.* In addition, compound **II96** could inhibit the growth of *Staphylococcus aureus* (MIC_100_ = 0.24 mM). The activity showed great value for the development of new alternative drugs comparable to the clinical drug silver sulfadiazine. The reference drug silver sulfadiazine displayed inhibition toward *Staphylococcus aureus* and *Escherichia coli*, with MIC_100_ values at 0.54 and 0.22 mM, respectively [243].

Given the enhancement of antibacterial activity upon complexation with active ingredient silver (I), water-soluble complexes ligated with 4-CH_2_OH imidazole with different counter ions were synthesized. Their two-fold-more susceptibility of Gram-positive bacteria than Gram-negative bacteria treated with a metronidazole-Ag (I) complex was in conformity. In view of the bacteriostatic parameter MIC toward Gram-positive bacteria, complex **II97** exhibited excellent activity, especially against *Staphylococcus aureus* (71 μM) and *Staphylococcus epidermidis* (96 μM), giving much lower MIC values than the standard drug silver sulfadiazine (252 μM and 224 μM, respectively). Such activity was also in charge of the variation of counter-ions, in which the CF_3_COO^−^ ion contributed to the best antibiotic activity [244].

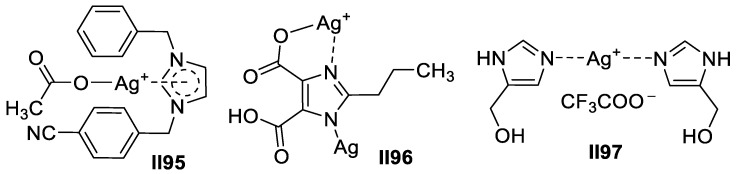



Silver compounds have long been used as antimicrobials, with their irreversible combination with the nucleotide bases of bacterial DNA in the form of silver ions. Silver sulfadiazine is the first silver metal organic compound used as an antimicrobial agent in the clinical treatment of burns. Comprised of alkylated imidazole ligands, Ag (I) supermolecule **II98** could effectively restrain the growth of *Escherichia coli*, *Bacillus subtilis*, and *Staphylococcus*. It was indicated in a structure-activity relationship study that the length of the alkyl chain impacted the antimicrobial effect. When *n* was equal to or below 3, the MIC values of *Escherichia coli*, *Bacillus subtilis*, and *Staphylococcus* were all over 20 μg/mL. As the alkyl chain continued growing, the antibacterial activity also grew, accompanied by relevant MIC values all below 20 μg/mL, especially when *n* was larger than 7, and the MIC values against both *Escherichia coli* and *Staphylococcus* reached 5 μg/mL. Further investigation showed that more lipophilic aromatic benzyls led to the evident loss of antimicrobial activity in comparison with the alkylated analogues of complex **II98**. Therefore, only proper lipophilic *N*-substituents were beneficial to antimicrobial exertion [245].

A diethyl-substituted imidazole silver (I) compound **II99** had promising antibacterial activity against *Acinetobacter baumannii* (MIC = 2–4 μg/mL). The magnitude of this value was similar to that of the antibiotic colistin (≤2 μg/mL), which was a last-resort treatment for Gram-negative bacterial infections [246].

Silver (I) imidazole compound **II100** was compared to new asymmetric imidazolium salts, namely 1-methyl-3-(2,5-dimethylphenyl)-acetamide imidazolium chloride. Compound **II100** showed remarkable efficacy against *Escherichia coli* and *Staphylococcus aureus*, which was much better than that of the reference drug azithromycin [247].

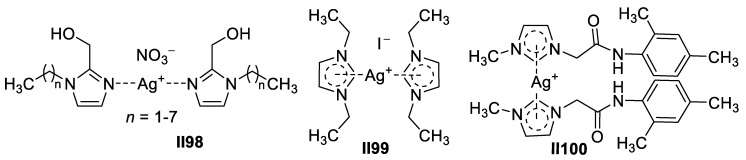



Using miconazole as a bioactive ligand, silver (I) complex **II101** containing nitrate counterions was obtained. Complex **II101** showed significantly higher activity against Gram-positive bacteria and yeasts than silver sulfadiazine and silver salt. In particular, complex **II101** displayed strong antibacterial efficacy against *Micrococcus luteus* ATCC 10240, being 23- and 22-fold more active than AgNO_3_ and silver sulfadiazine, respectively. Moreover, the efficacy against *Candida glabrata* ATCC 90030 and *Candida albicans* ATCC 102231 was better than that of miconazole [248].

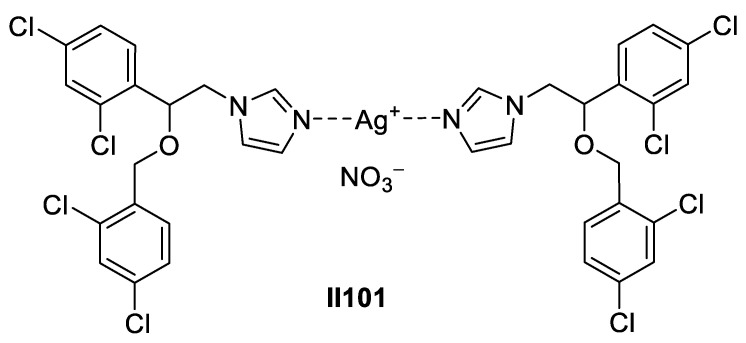



For imidazole Ag (I) compound **II102**, the inhibitory activity increased in unison with the growth of the length of the alkyl chain on the imidazole ring. Among these complexes, the *n*-amyl-substituted complex performed the best, with a MIC value of 25 μg/mL, which was twice and three times more potent than the n-butyl- and n-propyl-substituted compounds, respectively. It could be added that the antimicrobial activity was in positive correlation to the lipophilicity. Binuclear macrocyclic compound **II103** was more active toward bacteria, with its inhibitory activity twice as active than that of the *n*-amyl-substituted compound, rivaling that of the reference drug streptomycin [249].

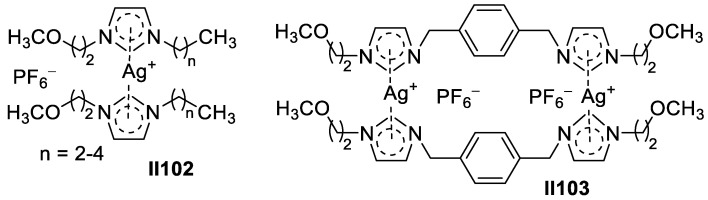



Inhibition toward *Escherichia coli* and *Staphylococcus* was also found in *N*-allyl imidazole Ag (I) compounds **II104a–c**, in which **II104-c** ranked first on the list of activity, with a MIC value of 12.5 μg/mL. The structure-activity relationship showed that a shorter alkyl or weaker electron-withdrawing group on the imidazole rings was undermining the antibacterial effect, with **II104-c** performing with two and eight times more potency than **II104-b** and **II104-a**, respectively [250]. This conclusion might imply that the length of the terminal alkyl chains and the type of substituents were both influential factors for the antimicrobial activity. An aliphatic alcohol-substituted imidazole could act as a bidentate ligand in **II105-b**, in which alcohol hydroxyl and nitrogen-involved imidazole partook in coordination. Compound **II105-b** exhibited strong inhibition against *Escherichia coli* and *Bacillus subtilis*, giving the same MIC value of 5 μg/mL. Findings on the structure-activity relationship showed that removing the *α*-phenyl fragment in the hydroxyl group made the product **II105-a** notably less active, with MIC values toward *Escherichia coli* and *Bacillus subtilis* that were more than 50 μg/mL. The consequence might reveal that the phenyl group increased the lipophilicity and helped the supermolecules more easily penetrate the cell membrane [251]. 

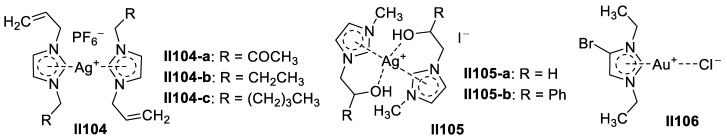



Gold complexes with *N*-heterocyclic carbene ligands could inhibit bacterial growth and display higher inhibitory effects on Gram-positive bacteria than Gram-negative bacteria. Among the screened compounds, the best performer, diethyl-substituted imidazole gold compound **II106**, clearly exceeded the reference drug auranofin toward *Escherichia coli* and two MASA strains, giving lower MIC values of 3.12, 0.64, and 0.64 μM, compared to 18.40, 2.30, and 2.30 μM for auranofin, respectively [252].

Prior to complexation, the thiosemicarbazone ligand was completely deprotonated, enabling it to act as a tridentate ONS donor ligand to the Zn atoms via the thiol group, imine nitrogen atoms, and phenolic oxygen atoms. Notably, in terms of the strain *Pseudomonas* being resistant to the reference drug vancomycin and nalidixic acid, imidazole-substituted complex **II107** (25 mm zone of inhibition) held the greatest promise for inhibition out of all the other complexes, which was rationalized by the imidazole co-ligand being capable of hydrogen bonding with cellular proteins. The results showed that the inhibitory activity of the complex against *Bacillus subtilis*, *Staphylococcus aureus*, and *Pseudomonas aeruginosa* was superior to that of the respective ligands. The mode of action was also attributed to azomethine units that could form hydrogen bonds and, thereby, interfere with normal cell activities [253].

Metalloporphyrin holds a key position in oxygen transport in hemoglobin and the process of photosynthesis in chlorophyll. Moreover, this was immensely explored in Zn (II), Cu (II), Co (II) and Zr (II) complexes with a wide antibacterial spectrum. Among these complexes, the magnesium porphyrin complex possessed more stable and simpler structures than the cobalt, iron, manganese, and other metal analogues. The inhibition zone diameter indicated that the tetrakis bromophenyl-hydrazine porphyrin complex presented an inhibitory effect on *Escherichia coli*, with a value of 16 mm. On the contrary, tetrakis bromophenyl-hydrazine porphyrin Mg (II) complex **II108** with an imidazole ligand gave lower values, at 14 and 15 for strains of *Pseudomonas aeruginosa* and *Enterococcus faecalis*, respectively. Generally, the higher activity of complex **II108** was verified in the diameter of the inhibition zone when compared to Co (II) analogues, which gave values from 6 to 14 mm when treating *Escherichia coli* [254].

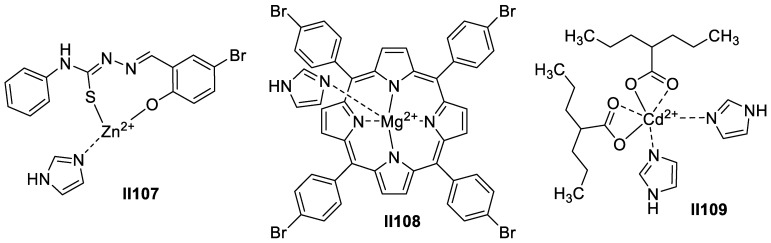



Valproic acid has versatile efficacy in chemotherapy containing epilepsy, manic depression, and anticancer fields. It was also revealed that valproic acid and imidazole have both been demonstrated as potent structural units in the realm of bacteriostatic application. Therefore, the Cd (II) complex **II109** formed by valproic acid and imidazole showed moderate to high antibacterial activity. Remarkably, significant antibacterial activity was obtained in the treatment of *Staphylococcus aureus* and *Bacillus subtilis*, with both having MIC values of 10 μg/mL, which were quite close to that of clinical drug imipenem (both values of 8 μg/mL). With respect to Gram-negative bacteria *Escherichia coli, Salmonella typhi* and *Pseudomonas aeruginosa* had more complex cell walls, and complex **II109** exhibited moderate activity, with MIC values ranging from 10 to 12 μg/mL, which indicated that the different structures of the cell walls accounted for the differentiation in their susceptibility [255].

Chosen as a molecular model of nitroimidazole derivatives, 5-nitroimidazole, which represented a broad-spectrum antimicrobial potential fragment, was incorporated into the ruthenium phenanthroline-substituted complex **II110**. The related research found that the antibacterial activity against *Bacillus subtilis* was effectively improved under the light. These findings provided the basic knowledge for the use of Ru (II) polypyridine complexes and other related nitroimidazole derivative projects as well as those complexes that use 5-nitroimidazole as a “photo linker” between Ru (II) centers and different types of bioactive compounds. This will help with the design of a new type of mixed antibacterial candidates, with activity that can be easily controlled by light to fight against antibacterial infection and drug resistance [256].

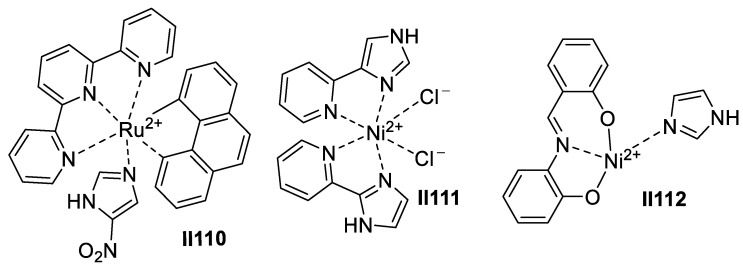



Nickel (II) supermolecules are also used in antibacterial applications. A pyridyl imidazole-based water-soluble nickel (II) complex **II111** was characterized by a distorted octahedral environment around the nickel (II) center, which was cis-coordinated by two pyridyl imidazole ligands and two chlorine ligands. The antibacterial test toward several clinical bacteria showed that compound **II111** had significant bactericidal properties in contrast with the clinical antibacterial ciprofloxacin. The cost-effective complex **II111** showed ROS-induced prominent antibacterial activities against *Escherichia coli* and *Klebsiella aerogenes* microorganisms [257].

Complex **II112** of the Ni (II) ion with a tridentate Schiff base ligand was synthesized from the reaction of the ligand sapH2 (salicylidene-2-aminophenol) with Ni(OAc)_2_ꞏ4H_2_O in the presence of imidazole. Nickel (II) complex **II112** with imidazole as the ligand showed remarkable antibacterial activity against *Staphylococcus aureus* [258]. 

Tridentate Schiff base ligands with ONO donor atoms have attracted a lot of attention due to their rich coordination chemistry, with the intrinsic capability to adopt diverse geometries. A tridentate Schiff base ligand and its mixed-ligand Ni (II) complex **II113** displayed good antibacterial activity against *Escherichia coli* and *Pseudomonas aeruginosa*. However, the antibacterial activity of this complex against *Staphylococcus aureus* and *Bacillus cereus* was lower than that of azithromycin. Surprisingly, complex **II113** had good antibacterial activity against *Pseudomonas aeruginosa*, but this species showed high resistance to azithromycin [259].

A mixed-ligand transition Ni (II) complex of an ionic liquid-based Schiff base was synthesized. The Schiff base coordinated with the metal ions as a potential bidentate ligand through the *N*-atom of azomethine and the *O*-atom of the phenolic group. Nickel (II) complex **II114** showed very significant responses for Gram-positive and Gram-negative pathogens, though the summative antimicrobial activity was marginally higher in Gram-positive bacteria than in Gram-negative bacteria [260].

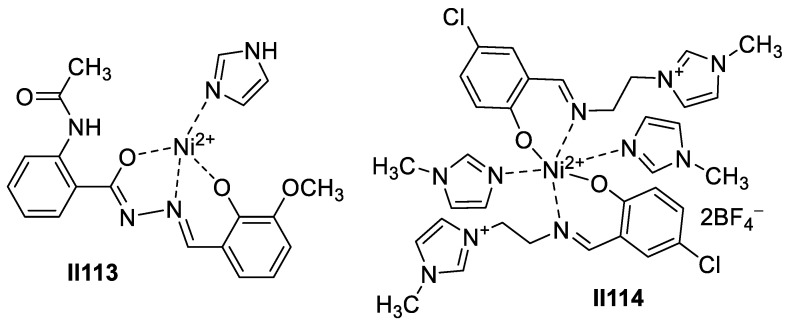



Copper (II) supermolecules also give good antibacterial potential. Copper (II) complex **II115** with 2-(imidazole-2-yl) heteroaryl ligands was observed to effectively suppress bacterial multiplication toward *Escherichia coli* and *Staphylococcus aureus*, with MIC values of 17.8 μmol/mL, but proved inactive toward the examined fungal strains [261].

Imidazole together with a phenol Schiff base could form Cu (II) complex **II116**, which could effectively inhibit the growth of *Staphylococcus*, *Bacillus subtilis*, and *Escherichia coli*. It was revealed in the structure-activity relationship that the antibacterial activities of its analogues varied with the 4-substituents on the aromatic rings and the *α*-substituents of ester. A bromine or oxhydryl group in the place of the nitryl moiety on the phenol ring and the methyl-substituting *α*-position of ester could cripple the antibacterial activity. It could be inferred from the result that the electron-withdrawing and lipophilic groups were beneficial to exert antimicrobial potency. In addition, when the benzene moiety was replaced by the naphthalene group, the antibacterial activity of the complex was lessened, and the MIC values were all above 75 μg/mL [262].

Another analogue **II117**, comprised of a similar tridentate ligand and a Cu (II) ion, exhibited significant inhibitory activity toward *Agrobacterium tumefaciens*, *Pseudomonas*, *Escherichia coli*, and *Streptococcus*. In particular, complex **II117** performed best toward *Agrobacterium tumefaciens*, with an inhibition zone that reached 26.5 mm at a concentration of 30 ppm, which was far more active than chloramphenicol and vancomycin [263].

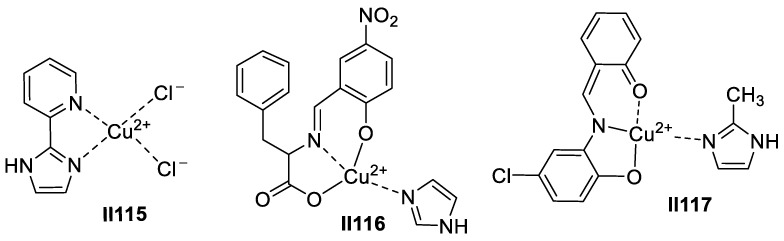



Pyrrole-substituted imidazole carboxylate **II118** is a tridentate ligand that was capable of forming supermolecules with Cu (II) hydrochloride, and the oxygen atoms on carboxylate and both nitrogen atoms on imidazole as well as a Schiff base all took part in the complexation. The supramolecular complex obviously inhibited better than the ligand salt toward *Escherichia coli*, giving a MIC value of 5 μg/mL, which was just as active as the marketed drugs amikacin and ciprofloxacin [264]. Tertiary amines with alkalescent nitrogen atoms as potent chelating sites are widely applied in the medicinal field. On the other hand, Cu (II) and tertiary amide-containing tridentate bis-imidazoles constituted supermolecule **II119**. Moreover, a dose-dependent effect was found toward *Bacillus subtilis*. If the administered concentration was raised from 1.25 to 2.5 μg/mL, the survival rate of bacteria was accordingly reduced from 63% to 8% [265].

Supermolecule **II120-b**, comprised of tridentate ligand with an imidazole group and a copper ion, could better curb the growth of *Pseudomonas* and *Bacillus subtills* than the clinical drug imipenem. In terms of the substituents on the phenyl group, complex **II120-c** substituted by an electron-withdrawing nitro group acted less actively than a methylated one but acted more actively than its analogue with unsubstituted phenyl complex **II120-a**. A methylated complex remained highly active when the central ion was replaced by a Zn (II) ion, indicating that the electron-donating group helpfully exerted the antibacterial activity [266].

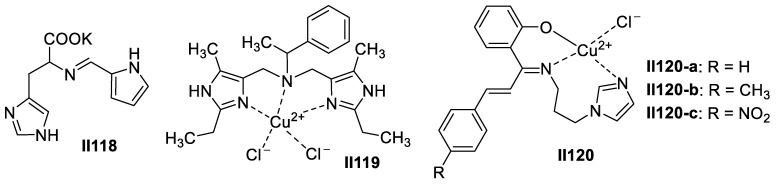



Two metal ternary complexes consisting of glutaric or glutamic acid and imidazole derivatives have been synthesized. After screening the antibacterial activity of the prepared compounds in vitro, it was revealed that Co (II) imidazole complex **II121** with glutamic acid as the ligand showed good antibacterial activity against *Pseudomonas aeruginosa*, and Cu (II) imidazole complex **II122** with glutaric acid as the ligand displayed potent antibacterial activity against *Bacillus cereus* [267].

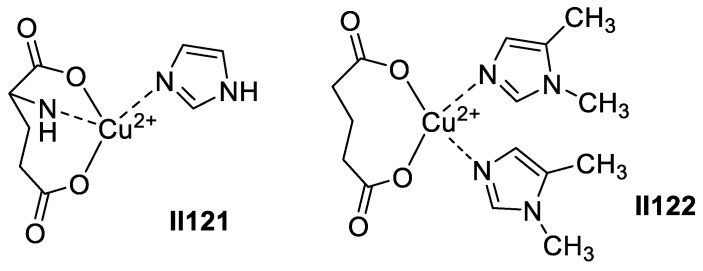



A copper (II) complex with pivalic acid and imidazole was prepared in the presence of sodium azide. An antibacterial assay revealed that complex **II123** was active against *Escherichia coli*, *Staphylococcus aureus*, and *Pasteurella multocida* bacteria, showing the strongest inhibitory activity against *Escherichia coli* [268].

A quinazoline ring system is the parent structure in various alkaloids, which has been widely recognized in synthetic chemistry and many bioactive molecules. An antibacterial assay using cefalexin as the reference drug indicated that the quinazoline-type ligand and its corresponding metal complexes containing an imidazole ring showed moderate activity against the tested bacteria. The antibacterial data showed that the metal complex was more active than the quinazoline-type ligand containing the imidazole ring. Compared to the Co (II) complex, copper (II) complex **II124** displayed stronger inhibitory activity. This increase in the antibacterial activity of the quinazoline-type ligand and complex was accompanied by an increase in concentration [269].

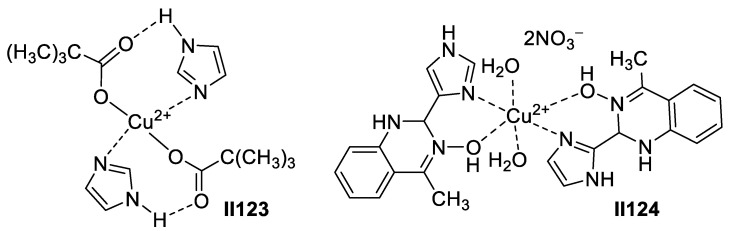



A cationic Co (III) triimidazole complex **II125** containing a Schiff base ligand showed higher activity against the tested bacteria and yeast than the clinical drug ciprofloxacin. A simulation of the binding of the complex to a GlcN-6-P synthase showed good agreement with the experimental results. The MIC and MBC values for the Co (III) complex were considerably lower than those of CoCl_2_, the ligand, and imidazole. This meant that low concentrations of the complex could inhibit the growth of the microorganisms as well as kill them. Complex **II125** was a potent antibacterial and antifungal candidate [270].

Iron (III) complex **II126**, comprised of an aryl-substituted imidazole ligand, has been synthesized, which favored a distorted octahedral geometry and showed promising antimicrobial activities for a wide range of microorganisms and cell lines. The interaction of this metal complex with calf thymus DNA was concluded to occur via an intercalative mode. Iron (III) complex **II126** exhibited powerful antimicrobial activity against various strains of bacteria and fungi, providing the following diameters for the zone of inhibition, which were comparable to those of tetracycline: 16 mm for *Pseudomonas aeruginosa*, 11 mm for *Escherichia coli*, and 17 mm for *Staphylococcus aureus* [271].

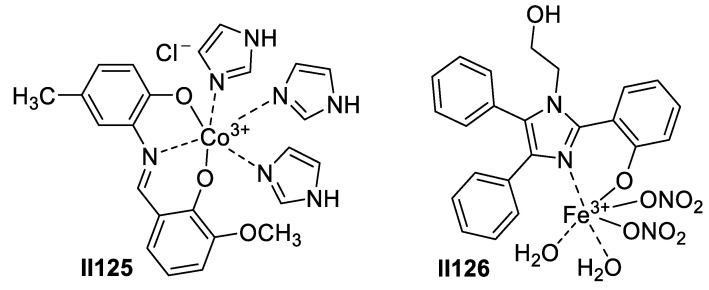



Tioconazole is a broad-spectrum antifungal drug containing imidazole, which has demonstrated antibacterial activity against tinea *Candida albicans* and yeast. In a search for novel antimicrobial metal-based therapeutic agents, mononuclear gold (I) complex **II127** formed with a tioconazole ligand significantly inhibited the production of bacterial pyocyanin, which was a virulence factor in *Pseudomonas aeruginosa*. In addition, complex **II127** displayed better antifungal activity than tioconazole against *Candida albicans* and *Candida kruisei*, with MIC values of 0.4 and 1 μg/mL, respectively [272].

Hydrazone is essential as an antiinflammation, analgesic, and antiplatelet pharmacophore. Hydrazone derivatives could also coordinate with imidazole and Mo (VI) to form supermolecule **II128**, with β-*N* but not α-*N* that joined in coordination. It was also shown in research that no acute toxicity was found in *Pseudomonas aeruginosa* (MIC > 100 μg/mL). Notably, complex **II128** displayed strong inhibition toward *Escherichia coli* and *Bacillus subtilis*, twice as potent as that of reference drug vancomycin, which gave all MIC values of 15.6 μg/mL. The structure-activity relationship showed that when the hydrogen atom at the 1-position in the imidazole ring was substituted by the methyl group, the resulting product was as active as **II128** toward *Escherichia coli* but acted quite weakly toward *Bacillus subtilis*, with a MIC value above 100 μg/mL. It was demonstrated that the 1-substituent on the imidazole rings was an important factor in the integral activity of the complex [273].

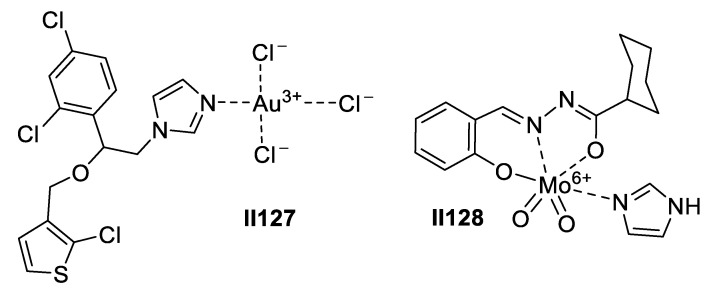



A hybrid of metronidazole and quinolone **II129** could bind with *Streptococcus pneumoniae* topoisomerase IV–DNA complexes via the noncovalent interaction to form a biosupermolecule, which exhibited good or even stronger antimicrobial activities when compared to the reference drugs. The hydroxyl group and nitrogen atom of imidazole formed two hydrogen bonds with the phosphates of the topo IV-DNA phosphodiester bond. The nitrogen and oxygen atoms at the nitro group of imidazole formed hydrogen bonds with the ParC helix a4 residue SER-79. These hydrogen bonds at the resistance mutation region might be the important reason that compound **II129** gave strong inhibitory efficacy against strains of quinolone-resistant bacteria such as MRSA. This highly active quinolone hybrid showed an appropriate range of pKa, log P, and aqueous solubility to pharmacokinetic behaviors and no obvious toxicity to A549 and human hepatocyte LO2 cells [274].

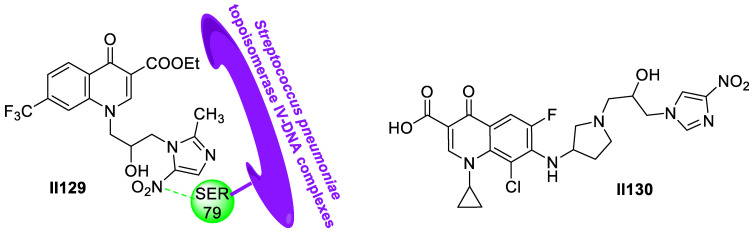



Quinolone derivative **II130** could intercalate into *Pseudomonas aeruginosa* DNA through a copper ion bridge to form a steady **II130**-Cu (II)-DNA ternary complex, which might further block DNA replication from exerting its powerful bioactivities. Compound **II130** initially formed a binary complex with Cu (II) ion, then formed a ternary complex with DNA by an intercalated binding form, and finally released a Cu (II) ion to form a supramolecular interaction between compound **II130** and DNA, which might further block DNA replication from exerting its powerful antimicrobial activities. Compound **II130** exhibited a low MIC value of 0.25 μg/mL against *Pseudomonas aeruginosa*, which was even superior to that of reference drugs norfloxacin, ciprofloxacin, and clinafloxacin, and gave low cytotoxicity against HEK 293, MEFS, and C2C12 cell lines [275].

Quinazolinone imidazole **II131** could bind to DNA via an intercalative mode to form the **II131**–DNA supermolecule, which might hinder DNA replication from exerting its powerful antimicrobial activity. Furthermore, the study of transportation behavior demonstrated that compound **II131** could associate with human serum albumin (HSA) through hydrogen bonds and van der Waals forces [276]. Imidazolyl hydrazone-based quinazolinone **II132** could also bind to DNA through the hydrogen bond between the oxygen atom of quinazolinone and the base DG-10 of *Staphylococcus aureus* DNA and, thus, exert antimicrobial potency [54].

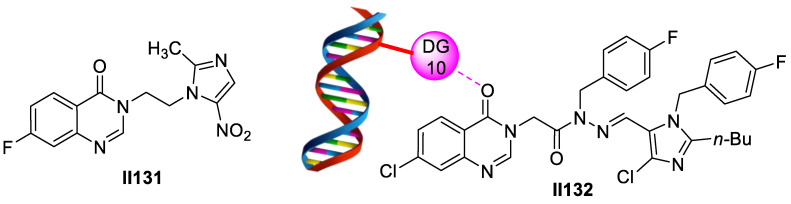



A methoxyphenyl nitroimidazole enol **II133** could interact with the topoisomerase II receptor through hydrogen bonds to form a supramolecular complex, which might block DNA replication from exerting its efficient bioactivities. The active molecule **II133** could rapidly kill sensitive *Pseudomonas aeruginosa* and had the ability to directly cleave *Pseudomonas aeruginosa* DNA, but there was no significant intercalation into *Pseudomonas aeruginosa* DNA. The nitro group could interact with the Arg162, Asn150, and Ser148 residues of Top II through hydrogen bonds. The nitrogen atom of the imidazole ring was in close vicinity to the Ala167 residue of topoisomerase II through a hydrogen bond. The hydroxyl group could form a hydrogen bond with the Ser148 residue. The imidazole molecule **II133** was also able to be stored and carried by human serum albumin via hydrophobic interactions and hydrogen bonds. Compound **II133** possessed stronger anti-*Pseudomonas aeruginosa* efficacy (MIC = 0.10 μmol/mL) than the reference drugs norfloxacin and metronidazole [277].

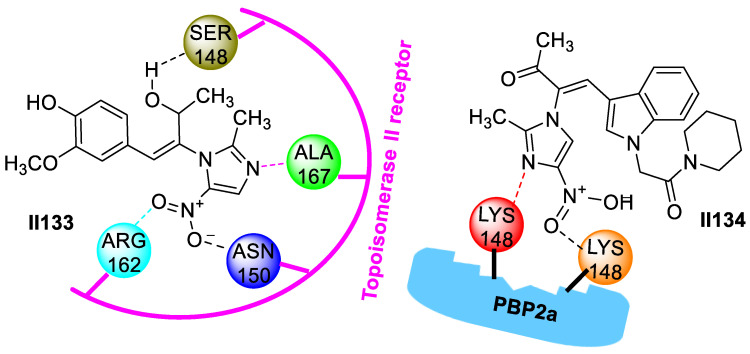



Enone-bridged indole nitroimidazole compound **II134** could bind with penicillin-binding protein 2a (PBP2a) to form supramolecular complexes, thus decreasing the expression of three relevant genes in MRSA and showing good inhibitory activity against MRSA (MIC = 1 mg/mL). The nitro and nitrogen atoms of the imidazole group could form key hydrogen bonds with LYS-148, since the PBP2a of MRSA may be a dazzling target for the screened compounds to inhibit the growth of MRSA. The active molecule **II134** had low cytotoxicity in normal lung epithelial cell line BEAS-2B and was able to be stored and carried by human serum albumin (HSA) [278].

Metronidazole-derived *p*-chlorophenylhydrazone compound **II135** could bind with lactate dehydrogenase (LDH) to form supramolecular complexes, thus reducing the activity of lactate dehydrogenase destroying the bacterial cytoplasmic membrane, and blocking cell metabolism. The oxygen atom of the nitro group and the hydrogen atom of the hydroxyl group could form hydrogen bonds with the THR-136 residue. The hydrogen atom of the phenylhydrazone moiety could be adjacent to the PHE-107 residue through a hydrogen bond. Compound **II135** possessed an excellent ability to suppress the growth of drug-resistant *Escherichia coli* (MIC = 0.5 μg/mL), being 16-fold more potent than norfloxacin (MIC = 8 μg/mL) [279].

Nitroimidazole derivative **II136** could associate with DNA-gyrase by means of hydrogen bonds and form a stable **II136**–DNA complex with MRSA DNA through an intercalation mode, which imparted potent bioactivity. Molecule **II136** could form with a gyrase–DNA complex by means of π–π stacking between the two carbonyl groups of the imidazo[2,1-b] benzothiazole backbone and the base pairs of DNA (DG-11 and DG-10). The nitrogen atom of the azole ring presented hydrogen bonding with the residue MET-1121. The nitro group showed close interaction with the residue AGP-1083, which signified the role of the nitro group in exerting strong bioactivity. Compound **II136** presented superior inhibit activity against MRSA and *Typhoid bacilli*, with MIC = 4 μg/mL and MIC = 1 μg/mL, respectively [280].

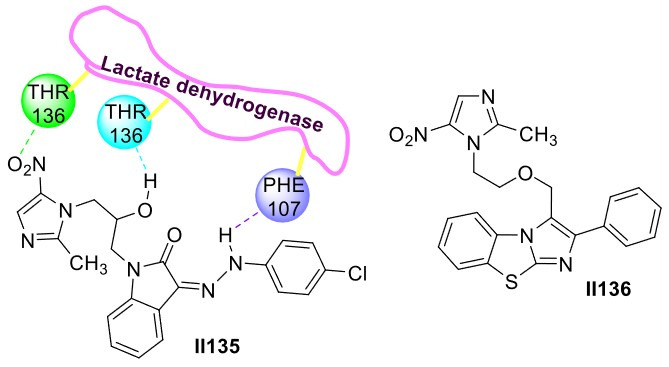



Sulfamethoxazole hybridized dihydropyrimidinone imidazole **II137** adjoined with the multiple amino acid residues of DNA gyrase in a mode of hydrogen bonds to form a supramolecular complex, which might act as a bacteriostatic by inhibiting DNA replication. The hydrogen atom at the N-3 position of the pyrimidinone and N-1 position of the imidazole ring could form a hydrogen bond with the ILE-370 and ILE-236 residues, respectively. A hydrogen bond also existed between one of the oxygen atoms of sulfonyl moiety and the LYS-374 residue. Compound **II137** was found to be extremely active against multidrug-resistant *Klebsiellar pneumonia* and *Acinetobacter baumannii* at a low concentration of 0.5 mg/mL, which outperformed norfloxacin and even clinafloxacin [281].

Sulfonyl-hybridized imidazolyl ethanol **II138** could bind with MRSA dihydrofolate synthetase at multiple sites to form a steady **II138**–enzyme supramolecular complex via hydrogen-bond interactions, which reduced the synthesis of the bacterial nucleic acid precursors, thus inhibiting the growth and reproduction of bacteria. The hydrogen of the hydroxyl group and the oxygen atom of the SO_2_ group formed two hydrogen bonds with the THR-62 residue. The hydrogen bonds could also be observed in the –NH and C=O sites, where they were bound with ASP-96 and ASN-115, respectively. Compound **II138** strongly suppressed the growth of MRSA (MIC = 4 μg/mL), which was 2-fold and 16-fold more potent than the positive controls sulfathiazole and norfloxacin, respectively [282].

Butene-modified sulfonamide imidazole hybrid **II139** was inserted into bacterial DNA isolated from clinical MRSA strains through noncovalent bonding to produce a supramolecular complex, thus exerting its strong antibacterial efficacy by impeding DNA replication. Compound **II139** demonstrated excellent inhibitory activity against MRSA (MIC = 1 μg/mL), which was 8 times higher than that of norfloxacin (MIC = 8 μg/mL) and 32 times higher than that of sulfathiazole (MIC = 32 μg/mL), and had no significant toxic effect on normal mammalian cells (RAW 264.7) [283].

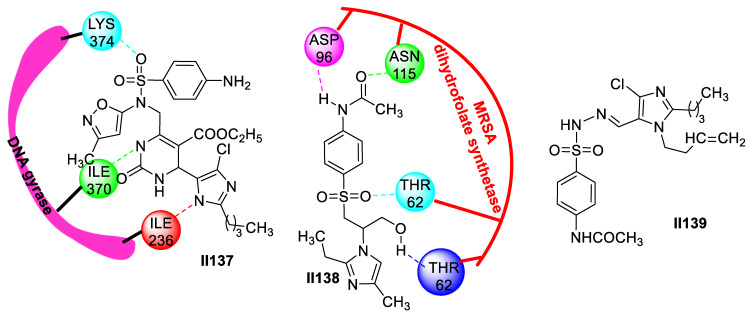



Imidazole-hybridized sulfonamide aminophosphate **II140** prepared by a one-pot three-component reaction could interact with dihydrofolate reductase through multiple sites’ noncovalent bonds to form a stable **II140**–enzyme supramolecular complex, thus exerting potent antibacterial potency. The nitrogen atom of imidazole was adjacent to the ALA-7 residue via a hydrogen bond. The oxygen atoms of the sulfonamide fragment and phosphorus oxygen double bond also took part in the hydrogen-bond interaction with the ASN-23 and THR-46 residues. Moreover, the hydrogen atoms of the benzenesulfonamide fragment could form hydrogen bonds with the SER-49 and ALA-145 residues. A series of biological experiments revealed that compound **II140** could prominently suppress the growth of *Escherichia coli* through multifaceted synergistic effects, with a low MIC of 2 μg/mL, which was four-fold more active in comparison to norfloxacin [284].

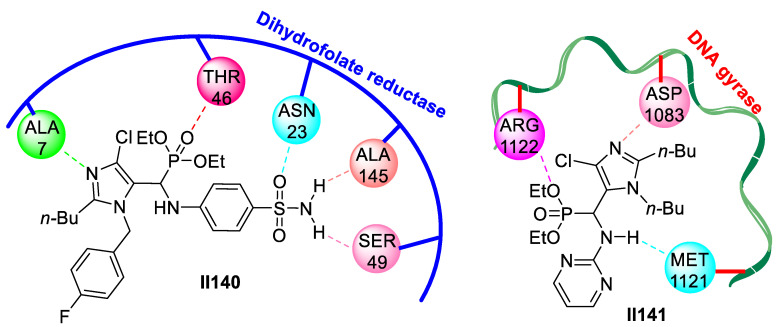



Imidazole-derived organophosphorus pyrimidine **II141** exhibited more potent anti-MRSA activity, with a MIC value of 4 μg/mL, in comparison to the clinical drugs chloromycin and norfloxacin. An experiment showed that compound **II141** could bind to DNA gyrase via hydrogen bonds and other weak interactions to form a biosupermolecule. The hydrogen atom of aminopyrimidine and the oxygen atom of the ester group could form hydrogen bonds with the MET-1121 and ARG-1122 residues of DNA gyrase. Moreover, there was a hydrogen bond between the nitrogen atom at the 3-position of imidazole and the ASP-1083 residue. These multi-site interactions might facilitate the formation of a **II141**-enzyme supermolecule and further block the function of DNA gyrase from exerting powerful antibacterial effects [285].

Piperazine-incorporated naphthalimide nitroimidazole **II142** could bind with DNA gyrase B via supramolecular bindings, which might be favorable to stabilize the **II142**–DNA gyrase B supramolecular complex and further block its function, thus exerting remarkable antibacterial activity. The oxygen atoms of naphthalimide and nitro moiety could interact with the ARG-20 and SER-202 residues through hydrogen bonds. Compound **II142** controlled more remarkable anti-*Acinetobacter baumannii* activity, with a MIC of 0.013 μmol/mL, than clinafloxacin [286]. Further work was completed to introduce a flexible chain-based amine into a naphthalimide-derived nitroimidazole, instead of a piperazine moiety, to construct potential antibacterial agents and study their supramolecular antibacterial behavior with topoisomerase II. The hydrogen atoms of the aminoethyl and hydroxyl groups in aminoethyl naphthalimide nitroimidazole **II143** could form hydrogen bonds with the SER-149 and ASN-91 residues, and the oxygen atoms of naphthalimide and the hydroxyl group participated in the construction of hydrogen bonds with the LYS-168 and ALA-167 residues of topoisomerase II. Moreover, a hydrogen bond was also observed between the oxygen atom of nitroimidazole and the ASN-163 residue. These multi-site bindings with topoisomerase II might be responsible for the good inhibitory efficacy of compound **II143** against *Proteus vulgaris* and *Shigella dysenteriae* [287].

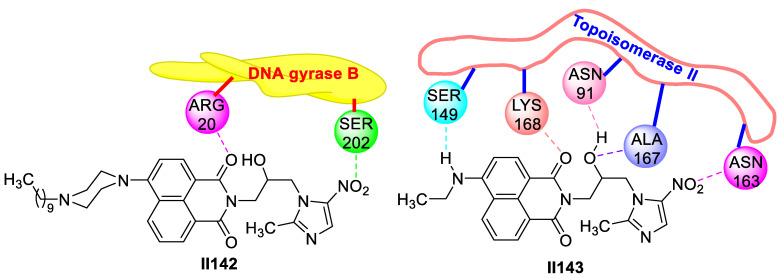



Berberine nitroimidazole hybrid **II144** could effectively suppress the growth of *Shigella dysenteriae*, with a MIC of 4 μg/mL, which was 64 times lower than that of norfloxacin (MIC = 8 μg/mL) but 32 times higher than that of berberine. Further transportation behavior study showed that compound **II144** could bind to HSA through electrostatic interactions to exert supramolecular transportation, and the calculated parameters revealed that the association was spontaneous, indicating that compound **II144** could be effectively stored and carried by HSA [288].

Imidazolyl berberine **II145** was adjacent to DNA topoisomerase IB via hydrogen bonds and other noncovalent bonding and could construct a **II145**-enzyme supermolecule, which might hinder its function and suppress bacterial growth. The oxygen atom of the methoxyl group was in close proximity to the ALA-182 residue via a hydrogen bond, and the nitrogen atom of imidazole could form a hydrogen bond with the HIS-180 residue of DNA topoisomerase IB. Moreover, compound **II145** was described as effectively intercalating into calf thymus DNA to form a **II145**–DNA complex and showed low toxicity by down-regulating ROS generation. These findings resulted in the potent antibacterial activity of compound **II145** with admirable safety against *Eberthella typhosa* ATCC14028 (MIC = 1 μg/mL) [289].

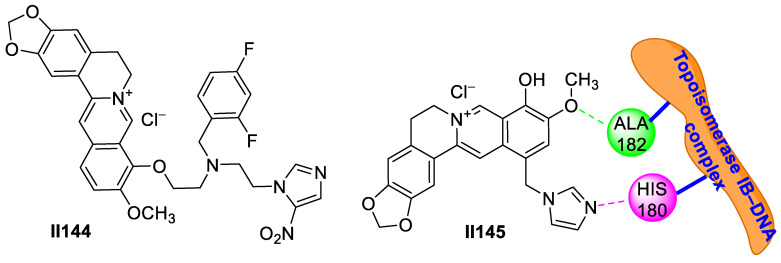



Tetrahydroberberine-derived nitroimidazole **II146** was found to bind to *Escherichia coli* DNA polymerase III through supramolecular interaction between the nitro group of compound **II146** and the GLY-219 residue, hindering its normal function, further blocking DNA replication, and, thus, inhibiting bacterial reproduction, with a MIC value of 0.003 mM [56]. Analogously, membrane-active iminotetrahydroberberine-corbelled metronidazole **II147** could interact with penicillin-binding protein (PBP) through noncovalent binding. The hydrogen atom of the OH group was in close proximity to the HIS-55 residue through a hydrogen bond. Another hydrogen bond was constructed by the oxygen atom of the NO_2_ group with the LYS–192 residues. Compound **II147** showed a broad antibacterial spectrum, with a quite low MIC value of 0.024 mM against *Pseudomonas aeruginosa*, being 63-, 62-, and 2-fold compared to the MIC value for berberine, metronidazole, and norfloxacin, respectively [290].

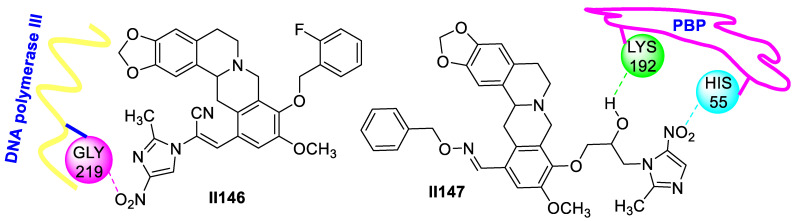



#### 2.2.2. Benzimidazole-Based Supermolecules as Antibacterial Agents

Benzimidazole is the fused ring of imidazole and benzene with a larger conjugated system than triazole, imidazole, pyrazole, etc. This special structure endows benzimidazole with a wide application in the fields of enzyme inhibitors [291], ion identifications [292], and particularly antimicrobial agents [293,294,295,296]. Recent research revealed that benzimidazole is low in toxicity with high efficiency, providing new hope for solving the aggravating drug-resistance problem [297,298].

Silver complexes as antibacterial agents have been a hot topic recently, especially silver sulfadiazine, a broad-spectrum antimicrobial medicine. Although the concrete antimicrobial mechanism of silver complexes has not been clarified, many experimental results found that a silver complex coordinated with nitrogen and oxygen atoms generally showed a higher antimicrobial effect. For example, a benzimidazole derivative with Ag (I) could form supermolecule **II148**, in which nitrogen atoms on benzimidazole rings acted as chelators. This compound could act with high activity toward *Pseudomonas aeruginosa* and methicillin-resistant *Staphylococcus aureus* (MRSA), with a MIC value of 3.9 and 7.8 μg/mL, respectively. Moreover, the activity was four- and two-fold more active, respectively, than that of silver sulfadiazine [299,300].

A class of asymmetrical Ag (I) compound **II149** substituted by *p*-cyanobenzyl behaved with a comparably moderate-or-high bactericidal activity, providing a diameter of the zone of inhibition of 21 mm for *Staphylococcus aureus* and of 20 mm for *Escherichia coli* [301].

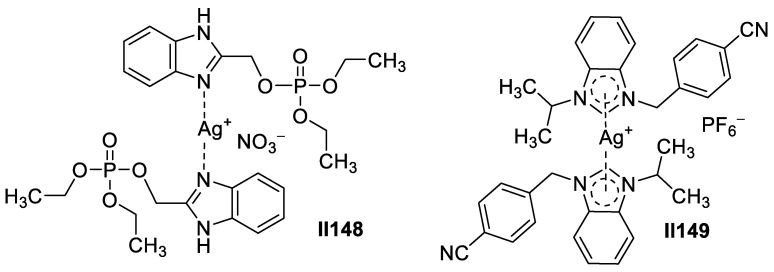



Silver, as the active constituent for antimicrobial usage, and *N*-heterocycle carbene, as a tunable building block for apt combinations, have been immensely explored to develop desirable drugs. In addition, the *p*-nitrobenzyl group is also applied in compound modulation on account of its broad involvements in the various types of drug engineering (e.g., analgesics, antipyretics, and antipsychotic drug design) for the nitrobenzene compounds. 

In this context, a class of asymmetrical *p*-nitrobenzyl-functionalized benzimidazole-based mono Ag (I) complexes and hexafluorophosphate Ag (I) complexes with a bis-NHC structure were prepared with yields in the range of 72–75%. A distinct activity differentiation was demonstrated between the complexes and their nearly inactive benzimidazolium hexafluorophosphate salt precursors. Furthering these studies, no significant difference in the inhibitory activity was observed between the mono Ag(I) acetate compounds **II150** and the bis Ag(I) **II151** ones, all giving MIC values varying from 16 μg/mL to 32 μg/mL for *Escherichia coli* and from 32 to 128 μg/mL for *Pseudomonas aeruginosa*. Similar MIC values were also provided in the treatment of *Bacillus subtilis*. Furthermore, a remarkably positive correlation of the inhibition activity with the length of the alkyl chain in the *N*-substituent supported the fact that the activity was enhanced by the correspondingly increased lipophilicity [302].

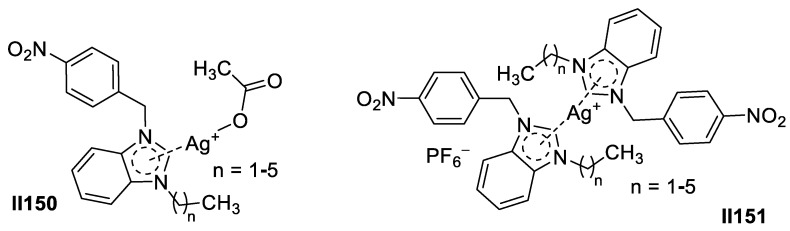



Transition metal complexes in the combination of NHCs are also explored as an improvement of the appended NHC systems. Different from the traditional means of introducing simple *O*-functional groups, carbenes tethered with *O*-heterocyclic systems were expected to be more capable in stabilizing the complex by altering the strength of the carbon-silver bond, thereby controlling the release of the active component [303]. As a consequence, a series of coumarin-appended Ag (I) imidazole complexes were prepared. Poor or negligible activity was observed in imidazolium and benzimidazolium salts against the two bacteria. Conversely, moderate activity, quantified as the diameter of the zone of inhibition and MIC values for complexes **II152**, was obtained toward *Staphylococcus aureus* and *Bacillus subtilis*, which was two times less active than reference drug ampicillin [304,305,306].

The main goal in the development of new bioactive compounds bearing coumarin-tethered benzimidazole is to access a class of derivatives that show appropriate stability under physiological conditions [307,308,309,310]. Structurally related ether-functionalized benzimidazolium hexafluorophosphate salts bearing methylcoumarin substituents **II153** have been synthesized. In the preliminary antibacterial evaluations, silver (I) compound **II153** displayed moderate activity against *Staphylococcus aureus*, *Escherichia coli*, and *Salmonella typhi* [311].

The *N*- or *O*-functionalized coumarin-benzimidazole silver complexes, when used as metal-based drug molecules, could improve stability and bio-efficiency by reducing decomposition rates. Silver (I) compound **II154**, formed by benzimidazole hexafluorophosphate salt and silver oxide, had an obvious inhibitory effect against *Escherichia coli*, with a MIC of 16 μg/mL, and showed moderate activity against *Staphylococcus aureus* [312]. 

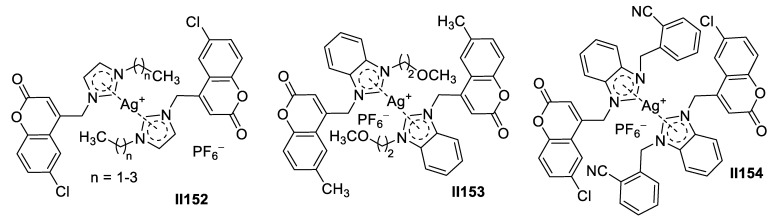



Nitrogen-functionalized benzimidazole silver (I) complexes were developed. The nitrogen functionalization on the ligand provided additional space for bonding or bridging to the silver (I) center. This unique ability facilitated the formation of coordination or supramolecular structures. Nitrogen-functionalized benzimidazole compound **II155** gave moderate antibacterial activity [313].

A *p*-isobutylbenzyl-substituted benzimidazole silver (I) compound **II156** displayed excellent biological activity, with a MIC value below 1.9 μg/mL. Compound **II156** could bind well with BSA by forming hydrogen bonds [314].

Silver (I) benzimidazole derivative **II157** emerged with widely antibacterial effects, including effects on *Escherichia coli*, *Staphylococcus aureus*, *Enterococcus faecalis*, and *Pseudomonas aeruginosa*. Subsequent research showed that the second methyl group on the *N*-benzyl benzene ring of **II157** could weaken the bioactivity. Moreover, the reduced activity could be also detected if there was no substituent on the phenyl ring in the complex. This result revealed that the introduction of an alkyl group could raise the lipophilicity to enhance the antibacterial potency, but the steric hindrance of the substituents harmed the interaction between the complex and target sites [315].

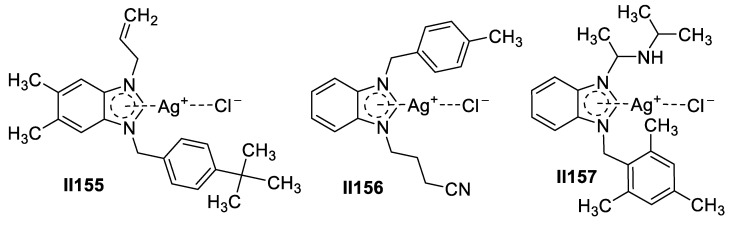



A large number of N-, O-, P-, or S-functionalized benzimidazolium salts have been synthesized to obtain NHC complexes that have different properties, which provide advantages in catalysis and other applications. Two silver (I) complexes **II158a–b** with ionic *N*-donor benzimidazoles showed a remarkable antimicrobial effect at normalized minimum inhibitory concentrations in the range of 33–268 μM against Gram-negative and Gram-positive bacteria as well as fungi [316].

The efficacy of metal complexes is very much based on the metal center and could be improved by the incorporation of multiple metal centers. Two binuclear silver (I) benzimidazole compounds **II159a–b** were synthesized, which were found to be good antioxidant and antimicrobial agents, with a MIC of 2.02–2.28 μg/mL against Gram-positive and Gram-negative bacteria, and were more efficient than the reference drug silver sulfadiazine [317].

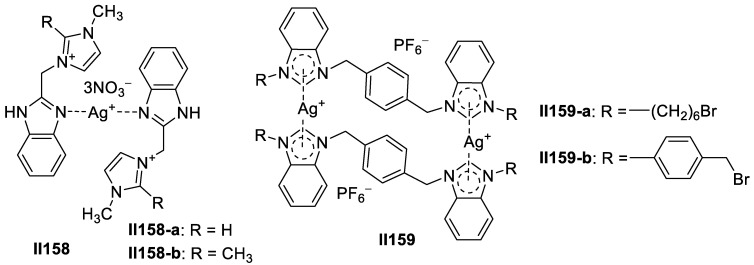



Dibenzyl-substituted Au (I) supermolecule **II160** acted actively toward *Staphylococcus* and *Enterococcus faecalis*, providing identical MIC values of 12.5 μg/mL. However, when methylated at all positions on the benzene rings, no inhibition was detected toward *Staphylococcus* and *Enterococcus faecalis*, while fungal isolates of *Candida albicans* and *Candida tropicalis* were strongly inhibited in their growth, with all MIC values at 12.5 μg/mL. At the same time, the structure-activity relationship revealed that the substituents on the nitrogen atoms of benzimidazole greatly affected the antibacterial ability of the product [318].

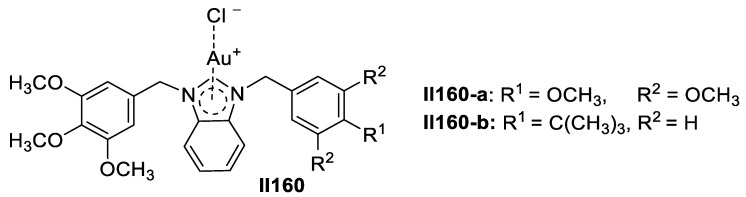



Benzimidazole thioether and the metal complexes **II161a–b** were explored not only as a corrosion inhibitor, due to their rich electron density, but also as a potential family of antibacterial agents. The inhibition zone diameters indicated the generally moderate and good antibacterial activities of these compounds toward *Staphylococcus aureus*, *Pseudomonas syringae*, and the yeast *Pichia pastoris*, but no detectable inhibition was found toward *Klebsiella pneumonia*. In addition, all ligands and organometallic compounds showed better efficacy against the latter than the reference drug benzylpenicillin sodium. It was noted that chelation was able to modulate the bactericidal activity, while the metallic center and ligand variation among these four complexes failed to induce any significant alteration of the inhibition zone diameters [319].

Macrocyclic polyamine 1,4,7,10-TATMP has been extensively investigated for its strong ability to coordinate potency and vital antibacterial activity. Benzimidazole-derived TATMP supermolecules **II162a–b** showed weak inhibitory activity against *Escherichia coli* and *Bacillus subtilis* but gave a good inhibitory effect against *Staphylococcus aureus*, *Micrococcus luteus*, *Pseudomonas aeruginosa*, and *Bacillus proteus*. A structure-activity relationship analysis discovered that the types of central ions and benzene-bridged linkers could affect the antibacterial activity of the complexes. Zinc complex **II162-a** performed a very potent inhibition effect toward *Pseudomonas aeruginosa* and *Micrococcus luteus*, with a corresponding MIC_50_ value of 1 and 0.5 μg/mL, which were 32- and 4-fold more active than the reference drug chloramphenicol, respectively. In contrast, complex **II162-b**, with a Cu (II) ion as the central ion, acted obviously less potently, with a MIC_50_ value for *Pseudomonas aeruginosa* that was over 512 μg/mL. In addition, if the bridged benzene in **II162-a** was *o*-substituted, lower antibacterial activity was observed, while the opposite was found when the bridged benzene in **II162-b** was *o*-substituted. The *o*-substituted copper complex **II162-b** was on par with the reference drug chloramphenicol against *Micrococcus luteus* and *Staphylococcus aureus*, all with MIC_50_ values of 2 μg/mL. In addition, the antifungal assay revealed that complex **II162-a** also had a potent inhibitory ability against *Candida albicans*, with a MIC_50_ value of 0.5 μg/mL, and the inhibitory ability was comparable to that of the reference drug fluconazole. This showed that complex **II162-a** had important medicinal value and was quite worthy of clinical research [320].

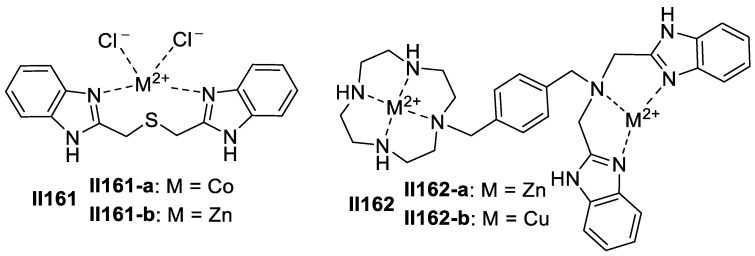



Ternary complex **II163** was made up of aliphatic amine-substituted benzimidazole, glycine, and a Cu (II) ion. It was demonstrated in a study that scarcely any activity was found in complex **II163** toward *Pseudomonas*, but good inhibitory activity was shown against *Bacillus subtilis*, *Staphylococcus*, and *Escherichia coli*, especially for *Bacillus subtilis*, with a MIC value of 6.25 μg/mL. Further research found that the complex performed more actively toward Gram-positive bacteria than Gram-negative bacteria, which was mainly attributed to the more complicated structure of the Gram-negative bacteria’s cell walls. In addition, the antimicrobial effect was decreased when a copper ion was replaced by a zinc ion, nickel ion, or cadmium ion, which was in accordance with their stability constant. What was revealed in the interaction mechanism was that the complexes prohibited peptidoglycan, the major component of cell walls, from being synthesized to exert antimicrobial capability [321].

Pyridine, as a significant heterocyclic biological electron equivalence of benzene, is also extensively used in medicine, pesticides, and the dyestuff industry. Pyridine-modified benzimidazole and glycinate could coordinate with a Cu (II) ion to generate complex **II164**, which performed well in fighting against *Bacillus subtilis*, *Staphylococcus aureus*, and *Salmonella*. A structure-activity relationship study showed that thiazole taking the place of pyridine at the C-2 position of benzimidazole led to less antibacterial activity, which was four times less potent than compound **II164**. Similarly, in Pd (II) supramolecular complex **II165**, the antibacterial activity of the complex decreased when the pyridine at the 2-position of benzimidazole was replaced by benzimidazole. The result showed that pyridine-substituted ligands played an important role in the antibacterial exertion [322].

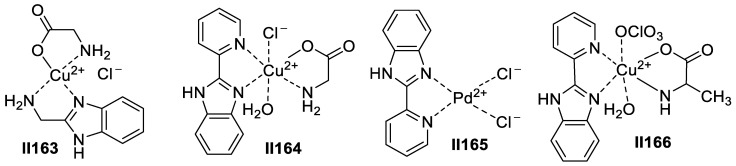



Pyridine-substituted benzimidazole derivatives could react with *L*-alanine and the Cu (II) ion in perchlorate to form supramolecular complex **II166**, which possessed a broad inhibitory spectrum toward *Bacillus subtilis*, *Staphylococcus aureus*, *Escherichia coli*, and *Salmonella*, better than their ligands. *L*-arginine or *L*-methionine was substituted by *L*-alanine to access a new complex with a supramolecular antibacterial activity equivalent to that of complex **II166**. It could be concluded that the *L*-*α*-amino side-chain structure had less of an influence on the inhibitory activity of this kind of compound [323,324].

Benzimidazole and a phenol substituted by aliphatic alcohol coordinated with the Cu (II) ion to produce supermolecule **II167**. Although this complex showed almost no inhibitory activity toward *Pseudomonas aeruginosa* and *Penicillium species*, the activity against *Staphylococcus aureus* and *Escherichia coli* was 70% and 87% of that of ampicillin, respectively. For *Klebsiella pneumoniae*, complex **II167** outperformed ampicillin. When a Cu (II) ion was replaced by Ni (II) and Zn (II) ions, an apparent decrease in antimicrobial activity could be observed [325].

Benzimidazole Cu (II) complex **II168** displayed a broad antibacterial spectrum and could effectively inhibit the growth of *Pseudomonas aeruginosa*, *Klebsiella pneumoniae*, *Bacillus subtilis*, *Streptococcus pneumoniae*, *Staphylococcus aureus*, and other strains. Its activity against *Klebsiella pneumoniae* and *Streptococcus pneumoniae* was weaker than that of the reference drugs, amikacin, gentamicin, and ciprofloxacin. However, complex **II168** exhibited better activity against *Pseudomonas aeruginosa*, *Bacillus subtilis*, and *Staphylococcus aureus* than amikacin and gentamicin, which was of promising research value [326].

Dipeptides act as a fundamental part in the biomolecules commonly found in hormones, immunomodulators, enzyme inhibitors, and neurotransmitters. Previous screening work showed that copper (II) peptide complexes incorporating glycyl-*L*-valine and 2-(2′-pyridyl) benzimidazole behaved with a desirably high affinity to the nucleobase and exerted antitumor activity, whereas the exploration into antibacterial application still remains underexplored. The order in which benzimidazole complex **II169** was sensitive to various bacteria was *Bacillus subtilis* > *Staphylococcus aureus* > *Escherichia coli* > *Pseudomonas aeruginosa*. Further, complex **II169** had an upper hand compared to the complex, which might be attributed to the better lipophilicity of 5-methyl-2-(2′-pyridyl) benzimidazole, which gave MIC values toward *Bacillus subtilis, Staphylococcus aureus*, *Escherichia coli*, and *Pseudomonas aeruginosa* of 13, 18, 26, and 64 μg/mL, respectively [327].

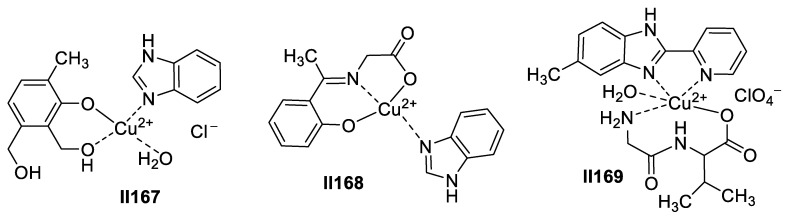



Metal complexes consisting of aromatic Schiff base ligands, especially those with imidazolium groups, were used in the design of molecule-based magnets as well as in the simulation of enzyme active sites due to their antimicrobial activities. *p*-Nitrophenyl-substituted benzimidazole Cu (II) complex **II170** showed much better antibacterial and antifungal activities compared to the other complexes and was supported as successful. Complex **II170** blocked the synthesis of proteins by disturbing the respiration process of the cell and further restricted the growth of organism [328].

Polypyridine compounds have a comprising prospective for application because of their unique conjugation system and benign coordinating ability. Thiazole-derived benzimidazole and fused bipyridine were able to form biligand complex **II171** in the hydrochloride of the copper ion, in which nitrogen atoms of benzimidazole, thiazole, and bipyridine were also involved in the complexation [329]. Supramolecular complex **II171** could effectively inhibit the growth of *Bacillus subtilis*, *Staphylococcus aureus*, *Salmonella*, and *Escherichia coli*, especially against *Bacillus subtilis* and *Escherichia coli*, and the MIC value was 16 μg/mL. If the fused-bipyridine ligand was replaced by a simple bipyridine, the inhibitory ability of the product was evidently weakened, with all MIC values over 100 μg/mL [330].

Sulfonamides and their *N*-substituted derivatives are extensively applied in the antibacterial field [331,332]. Many metal complexes of sulfonamides have been revealed to display better biological activity. Benzimidazole complex **II172** with sulfathiazole and a Cu (II) ion was synthesized and demonstrated strong inhibitory activity toward *Bacillus typhi* and *Fusarium oxysporum*, which was even better than that of the reference drug ampicillin. The mechanism of reaction demonstrated that the complex could disturb cellular respiration, obstruct the synthesis of proteins, and, thus, restrict the growth of microorganisms [333].

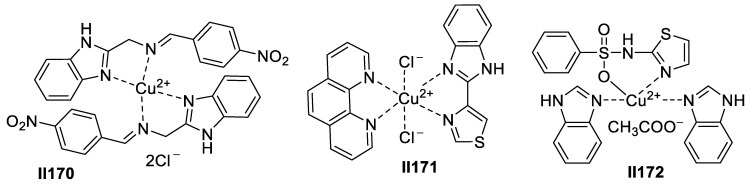



Antibiotics or clinical complexes with metallic ions are an effective treatment to eradicate gastrointestinal microbial infections. For instance, the proton pump inhibitor omeprazole complex is the potent killer for *Helicobacter pylori*. For benzimidazole derivative lansoprazole, it is used as proton pump inhibition, gastric acid secretion inhibitor, and strong metallic ligand. Stable lansoprazole Cu (II) complex **II173** could well restrain the growth of *Pseudomonas aeruginosa* and *Escherichia coli* far better than the corresponding ligands or the rival reference drug gentamicin. This property could be explained by chelation theory: the decrease in polarity and the rise of lipophilicity after forming supermolecules helped the compound to penetrate cells and afterward disturbed normal bioactivity [334].

Bipyridine, a Schiff base of bridged benzimidazole, and Cu (II) hydrochloride were also included in the formation of **II174**, which exhibited good inhibitory activity, with the MIC values ranging from 5.53 to 7.12 μg/mL. The structure-activity relationship showed that the fused-bipyridine Cu (II) complex **II174** acted better than a simple bipyridine-Cu (II) complex. Moreover, a copper (II) complex with nitro substituent was a better performer compared to one with a methoxy substituent. It could be concluded that the fused-bipyridine ligands were quite important for antibacterial interaction, and the electron-withdrawing substituents were helpful for strengthening antimicrobial activities [335].

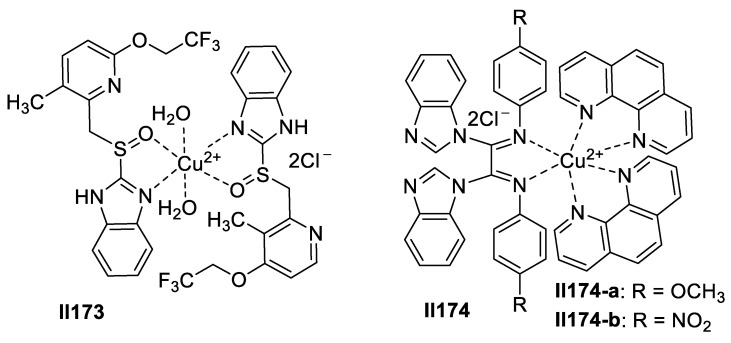



Alkylated benzimidazole and a cobalt ion could form a ternary complex **II175**, with an inhibitory ability toward *Pseudomonas aeruginosa* and *Bacillus subtilis* that was less potent than that of the reference drug ampicillin. However, this could be ameliorated by removing the methylene between the two carbonyls or substituting with cyclobutane for the methylene, resulting in stronger activities that were almost as active as those of the reference drug ampicillin [336].

A cadmium (II) benzimidazole complex was synthesized via the solvothermal reaction of metal salt with a well-designed semirigid benzimidazole ligand. Cadmium (II) benzimidazole complex **II176** showed the effective inhibition of *Dysentery bacillus* and *Staphylococcus aureus*. Importantly, this complex showed good photoluminescence properties in water, and its photoluminescence intensity was related to the properties of heavy metal ions. It displayed high selectivity and sensitivity for Cu (II) and Hg (II) ions in water, with a low detection limit [337].

Benzimidazole Ni (II) complex **II177-a** was proven to have a broad antibacterial spectrum and strong activity, which showed a strong inhibitory effect on *Bacillus subtilis*, *Staphylococcus*, *Enterococcus faecalis*, *Pseudomonas aeruginosa*, and *Escherichia coli*. The structure-activity relationship showed that the antibacterial activity of supermolecule **II177-b**, obtained by introducing the ester group at the *ortho* position of phenyl group of **II177-a**, was significantly reduced. This may be assigned to the instability of supermolecule **II177-b** and its low-fat solubility, which could not form an effective noncovalent bond with the active center, making it difficult to interfere with the metabolism of microorganisms [338].

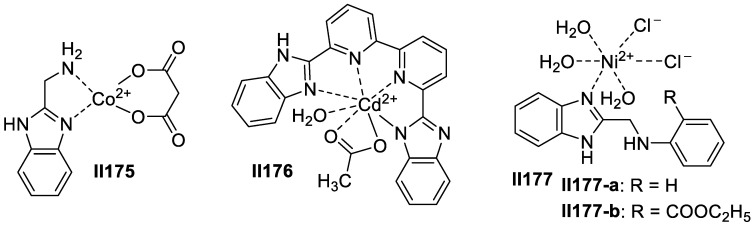



Iron is abundant in nature and acts as an extensive participant in most catalysis of molecular oxygen in vivo. Ferric chloride and tetradentate bisbenzimidazole could form supramolecular complex **II178**, which could effectively curb the proliferation of *Lactobacillus* at the administrated concentration at 40 μg/mL [339]. Complex **II178**, formed by sulfydryl benzimidazole and an Fe (III) ion, showed broad-spectrum antibacterial potential, inhibiting the growth of *Escherichia coli*, *Staphylococcus aureus*, *Bacillus subtilis*, etc., with an inhibition zone at more than 10 mm. Complex **II179** acted as potently toward *Escherichia coli* as chloromycetin and even more strongly toward *Staphylococcus* and *Bacillus subtilis* than the reference drug chloromycetin [340].

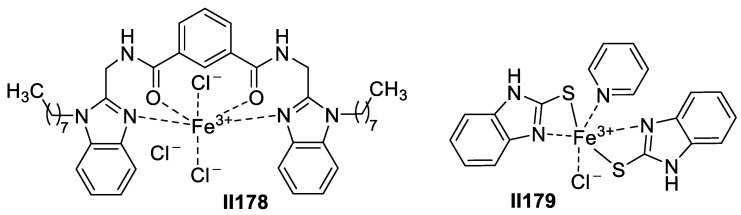



Ethyl-bridged benzimidazole with a La (III) ion could produce supermolecule **II180** in a high yield. Although the inhibition ability of the complex toward *Pseudomonas aeruginosa* was very weak, it could well inhibit the growth of *Bacillus subtilis*, and the activity was much higher than that of the corresponding ligand. The structure-activity relationship showed that the elongation of the alkyl chain between the two benzimidazoles only affected the antibacterial activity of complexes a little [341]. 

The strategy of organometallic derivatization has also garnered significant attention in medicinal chemistry. In this regard, ferroquine, a ferrocene-aminoquinoline antimalarial currently in Phase II combination studies, represents a landmark discovery. The aminoquinoline-benzimidazole Ir (III) complex **II181** displayed good activity against the chloroquine-sensitive NF54 strain of *Plasmodium falciparum*. Complex **II181** showed potent activity against the multidrug-resistant K1 strain (IC_50_ = 0.488 mM) and was proven to be active against *Mycobacterium tuberculosis* H37Rv (MIC_90_ = 0.488 mM) [342].

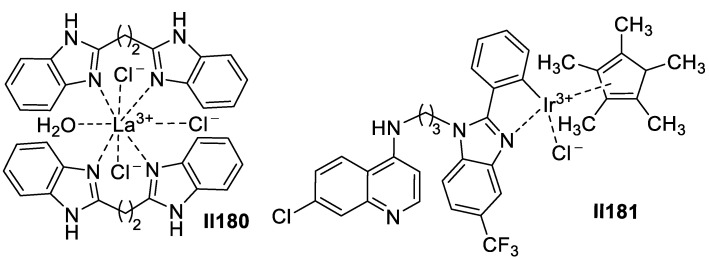



The *m*-aminophenyl substituted benzimidazole ligand **II182** and NiBr_2_ could form a complex where the nitrogen atoms in benzimidazole and the amino group of aniline took part in the chelation. This complex exhibited strong inhibitory activity toward *Staphylococcus* comparable to that of the reference drug ampicillin. The replacement of the coordination atom bromine by perchloric acid in the complex resulted in a weak ability to inhibit the growth of *Staphylococcus aureus*, and its activity was only half of the reference drug ampicillin. However, its antibacterial ability against *Klebsiella pneumoniae* was appreciable, and its antibacterial rate reached 80% of that of the reference drug ampicillin. However, the copper complex of **II182** possessed broad-spectrum activities toward *Staphylococcus aureus*, *Escherichia coli*, *Bacillus subtilis*, and *Klebsiella pneumoniae* [343]. Noticeably, the Ru (III) supermolecule of **II182** gave a weaker antimicrobial activity, and nearly no effect was found toward *Escherichia coli* [344].

The metal complexes of Zn (II), Co (II), Ni (II), and Cu (II) ions with the ligand 2-(thiophen-2-yl)-1-(thiophen-2-ylmethyl)-1*H*-benzo[d]imidazole **II183** were synthesized. Compared to free ligands, these metal complexes displayed better antibacterial activity. In the research, zinc (II) complex demonstrated effective antibacterial activity against *Bacillus subtilis*, with a MIC value of 0.312 mg/mL. The cobalt (II) complex showed high activity against *Pseudomonas aeruginosa* and *Staphylococcus aureus*, with the same MIC value of 0.156 mg/mL. The nickel (II) complex was more active against *Escherichia coli*, with a MIC value of 0.312 mg/mL. It was found that the Co (II) and Ni (II) complexes had good activities against all bacteria [345].

The copper (II) supramolecular complexes, formed by the mono ligand dibenzimidazole ethylene glycol **II184** with perchlorate, *L*-phenylalanine, and *L*-tryptophan, showed strong binding capacity with CT-DNA, and the binding constants were greater than 10^4^ M^−1^. In an antibacterial ability study, the apparent inhibitory activities of all the supermolecules were shown, especially for the perchlorate-involved copper complex and the tryptophane-containing Cu (II) complex, which were highly active toward *Escherichia coli*, sharing the same MIC value of 3.12 μM. The antibacterial ability of the copper phenylalanine supramolecular complex against *Staphylococcus aureus* was the best. A further antibacterial mechanism showed that the IC_50_ values of the perchlorate Cu (II) complex and the phenylalanine Cu (II) complex inhibiting topoisomerase I were about 20 mol/L, which was equivalent to the effect of a classical topoisomerase I inhibitor, camptothecin [346].

Ampicillin **II185** exhibited good inhibitory activities against both Gram-positive and Gram-negative bacteria, being the first broad-spectrum penicillin. It acted by prohibiting the relevant proteins from being synthesized, though its bioavailability and oral effect were poor. Fortunately, its supermolecule with benzimidazole and Cu(OAc)_2_ displayed broad active spectrum toward *Bacillus typhi*, *Staphylococcus*, *Pseudomonas aeruginosa, Escherichia coli*, *Bacillus proteus*, and *Klebsiella pneumoniae*. Thus, it is well-worth further investigation [347].

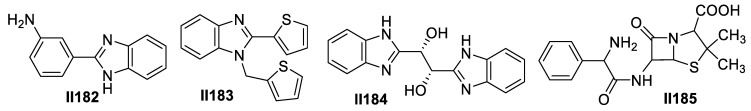



Benzene-bridged Schiff base benzimidazole derivative **II186** could form supramolecular complexes with Zn (II), Cu (II), Co (II), Ni (II), and Mn (II) ions, which could effectively inhibit *Escherichia coli*, *Bacterium fluorescens*, and *Staphylococcus* from proliferating. In particular, the zinc (II) complex performed the best, and all complexes outperformed ligand **II186** with their higher lipophilicity [348].

Thiophene, especially its *α*-thiophene derivatives, showed diverse potential in the application and synthesis of medicinal agents, pesticides, dyestuffs, materials, additives, etc. [349,350,351]. The skeleton structure of ligand **II186** was further modified by thiophene to produce benzimidazole-thiophene derivative **II187**, which coordinated with five metallic ions, and the resulting complex could display potent inhibitory activity against *Escherichia coli*, *Pseudomonas aeruginosa*, *Bacillus subtilis*, and *Staphylococcus* too. However, different from ligand **II186**, the inhibition activity of the Co (II) complex was the best after complexation, and the antibacterial zone was almost greater than 20 mm when the concentration was 60 μL [352].

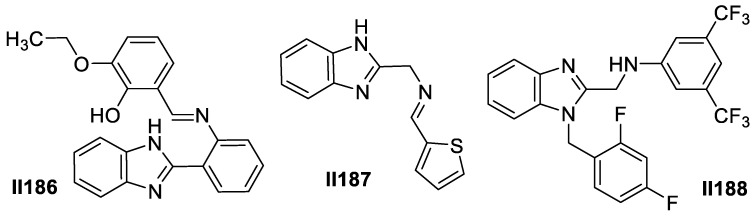



Difluoro-substituted *N*-benzyl benzimidazole **II188** could bind bacterial DNA by noncovalent bonds to form complexes, and synergies with clinical drugs chloromycetin and norfloxacin could lead to good antimicrobial efficiency. As compared to the reference drug, concomitant drugs had a lower dosage and broader antimicrobial spectrum toward *Staphylococcus aureus*, *Bacillus subtilis*, MASA, *Proteus*, *Escherichia coli*, *Pseudomonas aeruginosa*, *Salmonella typhi*, and other strains. In particular, they performed best toward MASA, with its activity being four and eight times more active than that of chloromycetin and norfloxacin. Moreover, the compound provided a fractional inhibitory concentration (FIC) value for all the examined bacteria that was lower than 0.5 [353].

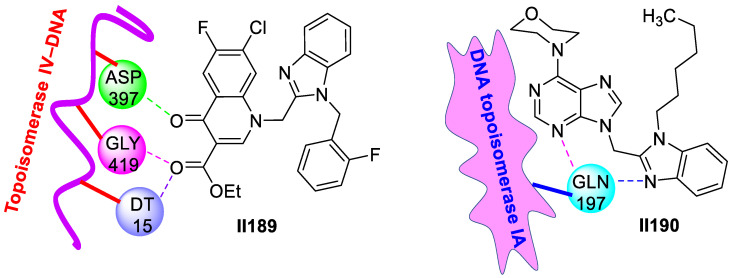



Benzimidazole-quinolone hybrid **II189** could bind to the topoisomerase IV-DNA complex through cooperative binding to stabilize the compound **II189**-enzyme-DNA ternary supramolecular complexes, which might block DNA replication and further display potent antibacterial activity. The oxygen atoms of the carbonyl group at the C-4 position and the ester group at the C-3 position could interact with the residues ASP-397 and GLY-419 of the topoisomerase IV-DNA complex and the base DT-15 of DNA through hydrogen bonds. Compound **II189** presented prominent anti-*Pseudomonas aeruginosa* activity, with a low MIC of 1 μg/mL, and showed a low-resistance tendency as well as imperceptible cytotoxicity [354].

Purine-derived benzimidazole **II190** was found to form a supermolecule with DNA topoisomerase IA through the use of hydrogen bonds through the nitrogen atoms at the 3-position of purine and the 3-position of benzimidazole, which might block its normal expression, thus suppressing bacterial growth (MIC = 1 μg/mL against *Staphylococcus aureus*). Moreover, compound **II190** could effectively interact with DNA isolated from *Staphylococcus aureus* by groove binding to the **II190**-DNA supramolecular complex, which showed that it could open up a new prospect as a supramolecular agent for coping with multidrug-resistant bacterial infections [355].

Fluorobenzyl benzimidazole derivative **II191** could effectively intercalate into calf thymus DNA to form a **II191**-DNA complex, which might block DNA replication and, thus, exert antimicrobial activities. Compound **II191** could bind with DNA topoisomerase IA through three hydrogen bonds through the use of the fluorine atoms and oxygen atoms in 5-fluorouracil with the residue LYS-423. Compound **II191** gave remarkable antimicrobial activities against *Saccharomyces cerevisiae*, MRSA, and *Bacillus proteus*, with MIC values of 1, 2, and 4 μg/mL, respectively [356].

Benzimidazole-appended 6-(*p*-tolyl)-2-aminopyrimidine **II192** prepared by the cyclization from ketenes and guanidine hydrochloride could interact with the DNA gyrase complex through the formation of hydrogen bonds. The hydrogen atoms of benzimidazole and the NH_2_ group were in close proximity to the residue ASP-1083 through hydrogen bonds. A further supramolecular study using calf thymus DNA showed that compound **II192** could bind to DNA via an intercalative pattern to form the **II192**-DNA supermolecule, which might make the DNA lose its normal function to suppress the growth of *Escherichia coli* DH52, with a low MIC of 1 μg/mL [293].

Benzimidazole-derived naphthalimide triazole could effectively intercalate into calf thymus DNA to form the **II193**-DNA complex, which could block DNA replication, exerting powerful antimicrobial activities. Compound **II193** exhibited good antibacterial activities, especially against *Staphylococcus aureus*, with an inhibitory concentration of 2 μg/mL, which was equipotent to that of norfloxacin (MIC = 2 μg/mL) and more active than that of chloromycin (MIC = 7 μg/mL) [357].

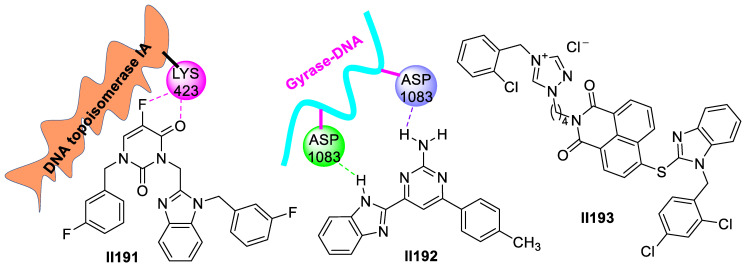



Pyrimidinone benzimidazole hybrid **II194** could bind with lactate dehydrogenase (LDH) to form supramolecular complexes and reduce the activity of lactate dehydrogenase, thus blocking cell metabolism. The oxygen atom of the carbonyl group at the C-2 and C-5 positions of the pyrimidinone participated in the formation of a hydrogen bond with the SER-135 and THR-136 residues, respectively. The hydrogen atoms at the N-3 position of pyridone also formed hydrogen bonds with the PHE-107 residues. The chlorine atom of benzene moiety and ARG-109, as well as the imidazole core in benzimidazole, PRO-110, and ARG-109, had hydrophobic alkyl and Pi–alkyl interactions, respectively. Compound **II194** possessed the strongest inhibitory effects on the growth of *Enterococcus faecalis* and *Pseudomonas aeruginosa*, with a MIC value of 1 μg/mL, which was lower than that of norfloxacin [358].

Benzimidazole-incorporated sulfonamide analogues **II195a–b** could effectively intercalate into calf thymus DNA to form **II195a–b**-DNA complexes, respectively, which might block DNA replication from exerting its powerful antimicrobial activity. Compound **II195-a** gave potent activities against Gram-positive bacteria and fungi, and 2,4-dichlorobenzyl derivative **II195-b** showed good activities against Gram-negative bacteria [359].

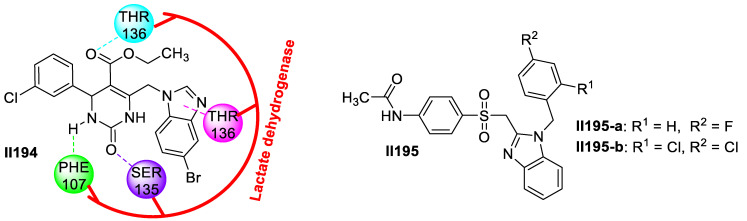



Berberine-benzimidazole derivative **II196** could effectively intercalate into DNA to form the **II196**-DNA supramolecular complex and cleave DNA, which exhibited good anti-MRSA, anti-*Escherichia coli*, and anti-*Salmonella typhi* activity, with low MIC values of 2–8 mg/mL, which were equivalent to or even better than those of the reference drugs. Human serum albumin could effectively transport compound **II196**, and the binding of compound **II196** to HSA was mainly through hydrophobic interaction and hydrogen bonding [360].

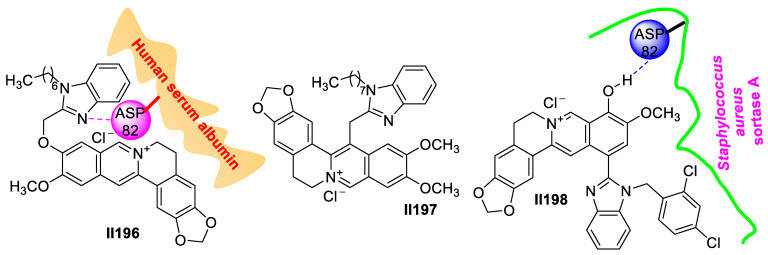



Methyl-substituted benzimidazole berberine **II197** could efficiently intercalate into DNA to form supramolecular complexes, which showed a promising candidate that not only exerted a strong activity (MIC = 0.25–2 μg/mL) and low cytotoxicity but also possessed a fast bactericidal capacity and low propensity to develop resistance toward *Staphylococcus aureus* and *Escherichia coli*, even after 26 serial passages. Compound **II197** displayed the ability to prevent bacterial biofilm formation at low and high temperatures and could significantly disintegrate bacterial membranes, markedly facilitating intracellular ROS generation [361].

The 2,4-dichlorobenzyl derivative **II198** could bind with DNA as well as *Staphylococcus aureus* sortase A to form supramolecular complexes, which not only showed strong activity against *Staphylococcus aureus* ATCC 29213, with a MIC value of 0.006 mM, but also effectively eradicated bacterial biofilm and exhibited low toxicity toward mammalian cells. The hydroxyl group at the C-9 position of the berberine skeleton could pick up a hydrogen bond with the oxygen atom of the carbonyl group in the ASP-82 residue. The active molecule **II198** could damage the membrane integrity and stimulate ROS generation [362].

Benzimidazole quinolone-based compound **II199** could form hydrogen bonds with the SER-79 of the topoisomerase IV-DNA complex through the hydrogen atom of the carboxyl group and intercalate into the superhelical DNA of the enzyme-DNA supramolecular complex, which might be responsible for the strong inhibitory efficacy against MRSA. Compound **II199** showed significant inhibition against the *Staphylococcus aureus* and *Escherichia coli* bacterial strains with low inhibitory concentrations (MIC = 0.0312–8 μg/mL) [363].

Bis-benzimidazole derivative **II200** could produce fluorescence quenching by forming the ground-state compound **II200**-BSA supramolecular complex under physiological conditions similar to those of humans. The probable quenching mechanism of the fluorescence of BSA by compound **II200** was mainly a static quenching procedure. The benzimidazole compound **II200** exhibited remarkable antimicrobial activities, which was comparable or even better than those of the reference drugs norfloxacin, chloromycin, and fluconazole [364].

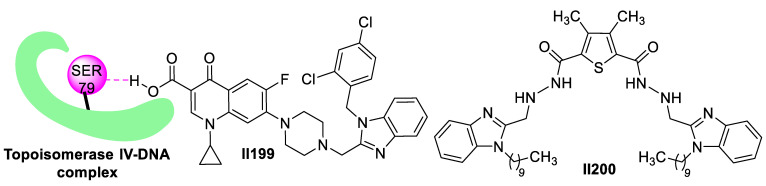



Triazolylpyrimidinediol **II201** could bind with dihydrofolate reductase (DHFR) to form complexes, thus hindering the synthesis of tetrahydrofolate and finally affecting the formation of bacterial nucleic acid and protein. Compound **II201** exhibited good anti-*Acinetobacter baumannii* potential, with a low MIC of 0.002 mmol/L. However, when the triazole group was replaced by other azoles such as benzimidazoles, imidazoles, and tetrazoles, the anti-*Acinetobacter baumannii* activity was significantly reduced [365].

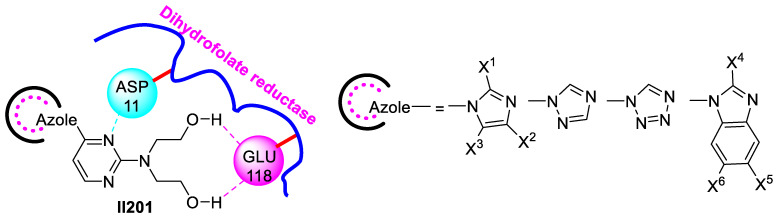



### 2.3. Imidazole-Based Supermolecules as Antifungal Agents

Fungal infection, which is quite normal and frequent, is responsible for the deaths of immunocompromised patients, as one recent factor [366,367,368]. The first developed azole antifungal drugs were of the imidazole type; so far, a large number of imidazole-based antifungal drugs have been successfully marketed and widely used in clinical settings. For example, ketoconazole, miconazole, econazole, and other many imidazole-based drugs showed a benign antifungal ability. Along with the emergence of drug-resistant fungal strains caused by the wide use or even abuse of antifungal drugs, the diminishing therapeutic ability of current antifungal drugs has brought a severe threat to human health [369,370,371]. Hence, it has become more and more important to develop safer and more efficient antifungal agents [372]. In terms of complexes, they have been increasingly researched for their good hydrophilicity, bioavailability, and other advantages, revealing their unique value within antifungal medicinal science. In recent years, research toward developing imidazole-based complexes as antifungal agents has shown an increasing tendency [373,374].

Fluobenzyl pyrimidinetrione imidazole compound **II202** might bind with sterol 14α-demethylase (CYP51) to form a supramolecular complex through hydrogen bonds, which inhibited the formation of fungal cell membranes and, thus, achieved antifungal effects. The carbonyl group between two nitrogen atoms and the residue TYR-118 formed a hydrogen bond. There was a coordinate bond existing between the N atom of the imidazole ring and the Fe (II) ion of heme, which might be attributed to the existence of the butyl group enhancing the electron cloud density of the N atom. Compound **II202** exerted favorable inhibition toward *Candida Albicans* (MIC = 0.002 mM), being 6.5-fold more active than the clinical antifungal drug fluconazole (MIC = 0.013 mM) [375].

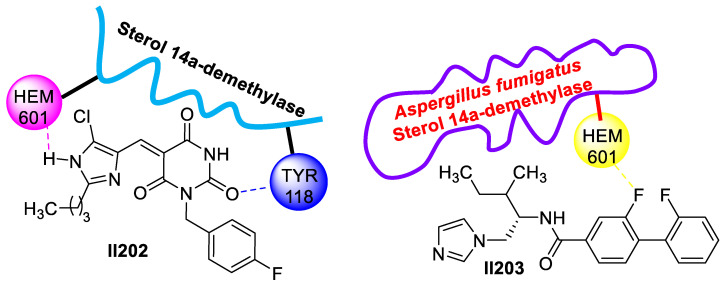



Imidazole-substituted L-amino alcohol derivative **II203** could bind with *Aspergillus fumigatus* CYP51 to form a supramolecular complex through hydrogen bonds, which inhibited the formation of fungal cell membranes and, thus, achieved antifungal effects. The 3-F atom on the biphenyl group could form a hydrogen bond with ILE-373. Compound **II203** exhibited excellent activities against *Candida albicans*, *Cryptococcus neoformans*, and *Candida tropicalis* and displayed antifungal activity against *Aspergillus fumigatus*, with a MIC value of 1 μg/mL. Compound **II203** was almost nontoxic to A549 cells at 50 mM/L and exhibited excellent stabilities in human plasma [376].

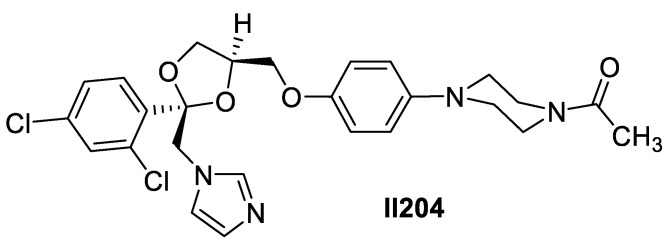



Drug carriers such as perylene and cyclodextrin have become a quite significant research field [377,378]. For example, *β*-cyclodextrin has a moderate cavity with a strong inclusion ability and can be easily absorbed and decomposed, leaving no toxicity in the human body. If adding small molecules into the cavity of *β*-cyclodextrin, the stability and solubility of drugs can be apparently ameliorated, providing efficient solutions for low water-soluble drugs. Ketoconazole **II204** was an imidazole-based broad-spectrum antifungal agent, with a drug efficiency that was limited due to its poor hydrophilicity causing low bioavailability. In further investigations, elevated solubility in comparison with the commercial suppository and antifungal activity better than that of ketoconazole were observed after the formation of the inclusion supramolecular complex of ketoconazole and *β*-cyclodextrin [379]. 

Bis imidazole Ag (I) complex **II205** had a fairly strong activity toward fungus, with a MIC_80_ value against *Candida albicans* of around 7.6 μM, acting far more actively than reference drug fluconazole. A Structure-activity study showed that, after the introduction of methyl onto the N-1 or 4-position in the imidazole rings, the activity of complexes against *Candida albicans* was reduced by seven times and 1 time, respectively [380]. Anthracene carboxylic acid and two molecules of imidazole derivatives could form Ag (I) complex **II206** with benign inhibitory activity toward *Candida albicans*. Subsequent research found that unsubstituted imidazole rings improved the antifungal capability, but electron-withdrawing groups introduced into the imidazole ring brought about the opposite effect, decreasing the antifungal activity. This demonstrated that substituents impacted the antifungal exertion of the complexes [381,382].

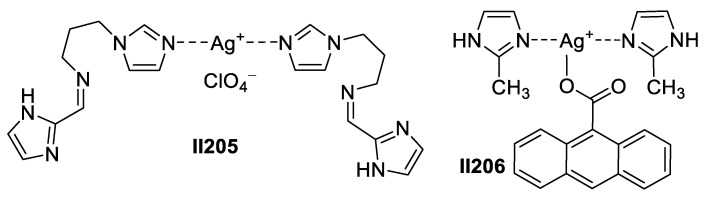



Lansoprazole **II207** is a kind of proton pump inhibitor usually used for treating peptic ulcers, and it has no antifungal activity. Antifungal activity could be found in the lansoprazole complexes of metallic ions such as Gd (III), Th (IV), and Ce (IV) transition metals ions, which could effectively inhibit the growth of *Aspergillus niger*. The yield percentage of the complexes ranged from 80% to 90%. A further investigation revealed that all the complexes showed moderate antifungal activity against *Aspergillus niger*, of which the Gd (III) complex was excellent in refraining from fungal infection and is well worth further researching [383].

Fluconazole **II208**, an antifungal agent with less hepatic toxicity, orally available administration, and high bioavailability, had a prominent curative effect toward deep fungal infection, especially *Albicans saccharomyces* and novel *Cryptococcus neoformans*, thus being designated as a choice drug against fungal infection by the World Health Organization. Nevertheless, the long-term use of fluconazole has given rise to drug-resistant strains. What is worse, the curative effect on non-*Candida albicans* was not good, urgently calling for the development of novel antifungal agents [384,385]. In the subsequent explorations, taking advantage of the synergistic effect between fluconazole and the other active molecules via noncovalent interaction was the feasible solution to drug tolerance. The combination of fluconazole and benzimidazole derivative **II209** not only inhibited *Candida albicans* well but also *Aspergillus flavus* and *Candida mycoderma*, all with a FIC index below 0.5, meaning they are quite worthy of further study in clinical settings [386].

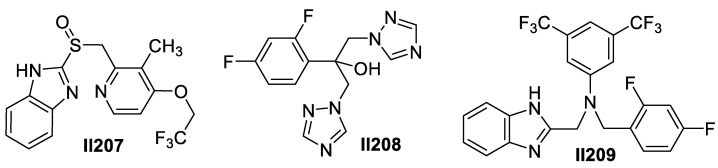



A hybrid of hydroxyethyl naphthimide and benzimidazole **II210** could constitute a biosupermolecule through its multiple hydrogen bonds and other noncovalent interactions with the cytochrome P450 reductase (CPR) from *Candida tropicalis* that hinder CPR biological function. The O atom of the carbonyl group at the 1-position in naphthalimide formed a hydrogen bond with the H atom of the amino group in SER-441, and the H atom of the hydroxyethyl segment could interact with the O atom of the carboxyl group in ASP-677. The N atom and H atom of the benzimidazole fragment took part in the hydrogen bonds’ reciprocity with the TRP-679 and GLU-460 residues. Compound **II210**, with its excellent anti-*Candida tropicalis* efficacy (MIC = 4 μg/mL), possessed low cytotoxicity, a safe hemolysis level, and less susceptibility to inducing resistance [387].

Benzimidazole-hybridized cinnamate **II211** might bind with the sterol 14α-demethylase (CYP51) to form a supermolecule through hydrogen bonds, which achieved antifungal effects. The carbonyl group and the residue ILE-471 formed a hydrogen bond, and the O atoms in the nitro group formed hydrogen bonds with the residue ARG-381. With an MFC = 125 μg/mL, compound **II211** demonstrated excellent activity against *Candida Albicans*. Furthermore, compound **II211** was shown to have better activity than the clinical drug griseofulvin against *Candida krusei*, with an MFC = 200 μg/mL [388].

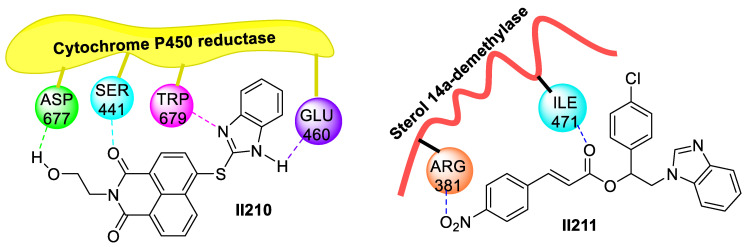



The efficacy of silver complexes against bacteria and cancer cells depends on a number of factors that are strictly controlled by the characteristics of the ligands and their requirements for both steric and electronic properties. Three symmetrical and nonsymmetrical *N*, *N*-disubstituted benzimidazolium salts were synthesized as *N*-heterocyclic carbene precursors. These salts were treated with silver oxide to afford their corresponding mononuclear Ag (I) complexes. Complex **II212** displayed improved the antibacterial efficacy against *Candida glabrata* and *Candida albicans*, comparable with the standard drug fluconazole (MIC = 6.25 μg/mL for *Candida albicans*; MIC = 3.12 μg/mL for *Candida glabrata*). All the complexes showed the same activity against *Candida glabrata* and *Candida albicans* (MIC = 50–100 μg/mL). Complex **II212-a** substituted by *p*-chlorotoluene and complex **II212-b** substituted by *p*-cyanotoluene gave the best values, with MICs of 50 μg/mL for the fungal strains [389].

By coincidence, the vinyl-substituted silver complex **II212-c** was also highly active against *Candida albicans*, with an inhibition zone at 15 mm [390].

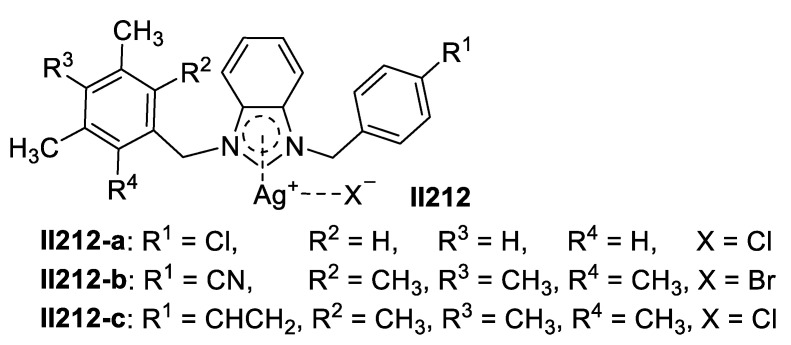



Benzimidazole Schiff base Cu (II) complex **II213** possessed the inhibition zone of *Aspergillus niger* at 19.5 mm when administered at a concentration of 50 μg/mL, which was slightly weaker than that of nystatin, while the inhibition zone against *Candida albicans* was equivalent to that of nystatin. Subsequent research found that changing central ions to Ni (II) or Ag (I) ions apparently decreased the antifungal activity. It was revealed by the structure-activity relationship that the ester group in the place of the cyano group was also detrimental to antifungal activity [391].

The copper (II) and zinc (II) complexes of the Schiff bases have been the subject of much research work, presumably due to their biological role and synergetic activity with the drug. The Schiff base metal complex **II214** showed greater activity toward the fungus *Canidia albicans* than the reference drug nystatin. Chelation reduced the polarity of the metal ions mainly because of the partial sharing of its positive charge with the donor groups and possibly the π–electron delocalization within the whole chelate ring system; thus, this process of chelation increased the lipophilic nature of the central metal atom, which in turn favored its permeation through the lipid layer of the membrane [392].

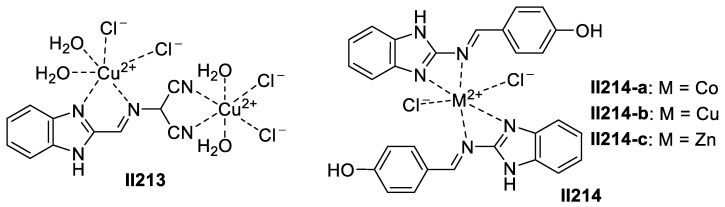



That gold and its complexes show remarkable antimicrobial activity against various fungi and their pharmacological properties has been known since antiquity. Disruption of the bacterial cell wall and cytosolic membrane in different bacterial strains induced by gold (I) complexes is postulated as a possible mechanism of their antibacterial action. Despite the medical success of various gold-based drugs and antifungal azole drugs, new compounds are being explored to improve existing treatments that lead to severe side effects or the development of drug resistance.

The chemistry of Au (III) has been highlighted for its significant cytotoxicity against biofilm-mediated antibiotic-resistant infections. A gold (III) complex containing *N*, *N*-pyridyl benzimidazole derivative **II215** modified with an ethyl group in benzimidazole gave good antifungal activity against *Canidia albicans* and *Cryptococcus neoformans*, and the MIC values were not more than 0.25 μg/mL. For this Au (III) complex, it was preferred to decorate the benzimidazole moiety with the ethyl group, rather than the sulfonate or phthalimido, to enhance the antifungal activity [393].

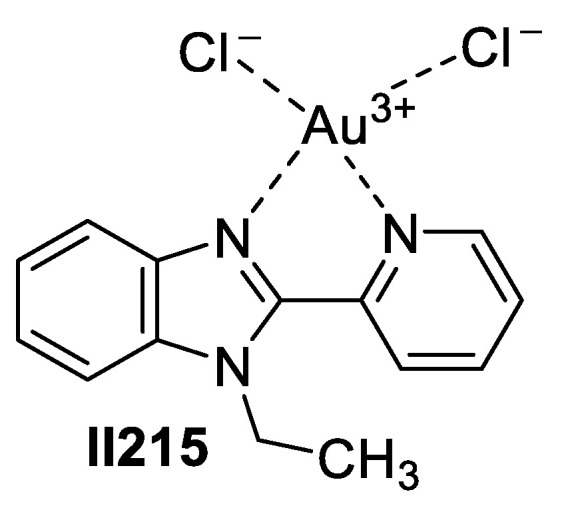



### 2.4. Imidazole-Based Supermolecules as Antiparasitic Agents

Parasitic disease is widely spread all over the world as a common ailment that brings great damage to society and the economy in tropical and subtropical areas [394]. For the time being, no vaccine is used to guard against the parasitic diseases caused by protozoa [395]. The currently used first-line drugs such as amphotericin B, miltefosine, and Sb(V)-based organic salts have been confronted with not only high prices and difficulty in drug administration but also several side effects during treatment in addition to the emergence of resistant insects [396,397,398]. Hence, developing novel structures to treat protozoa has become a significant topic. Imidazole-involved fragments have latent biological activities, so they are widely used to construct novel medicinal molecules [399,400]. Among the drugs, imidazole-based heterocyclic molecules have become a heatedly discussed topic in research devoted to antiprotozoal drugs because of their safety, high efficiency, convenient administration, and other advantages [401].

Leishmaniasis is an underlying lethal parasitic ailment caused by leishmania. It has been reported that phosphoglycerate mutase (PGAM) had a great significance in the glycolysis and heterogenization of glucose, the processes leishmania was highly dependent on to sustain its life activities. Thus, developing drugs capable of inhibiting the activity of PGAM provided new hope for leishmaniasis treatment. At 10 μg/mL, supermolecule **II216**, formed by an imidazole carboxylic acid derivative and V (III) ions, could inhibit the activity of PGAM and 25% growth of leishmania after 24 h of treatment [402]. 

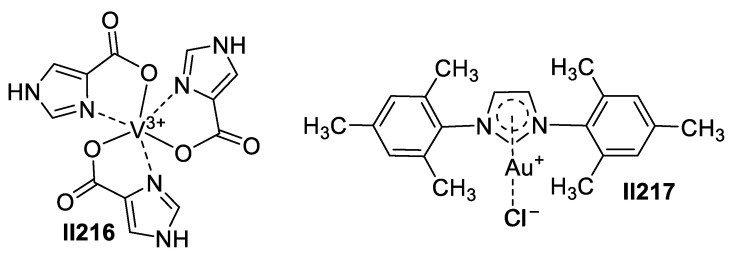



Considering the limited effectiveness and high toxicity of the current drug bank for the treatment of American cutaneous leishmaniasis, new options are urgently needed. Au (I) complexes were widely considered for their thiophilic feature and anti-inflammatory, anticancer, and antiparasitic activities. Two-mesitylene-substituted imidazole Au (I) compound **II217** was active against *Leishmania proflagellates* at low micromolar concentrations, with a 50% effective concentration of 1.57 μM. Compound **II217** was proven to be a potential candidate with a selectivity index of 13 against both bacteria. Further studies showed that compound **II217** induced significant changes in parasite morphology and membrane permeability. In addition, compound **II217** was able to reduce the residual activity of three leishmania recombinant cysteine proteases, which have been suggested as possible targets for the Au (I) complex. These promising results opened the possibility of exploring Au (I) complexes as leishmania molecules for further screening in in vivo infection models [403].

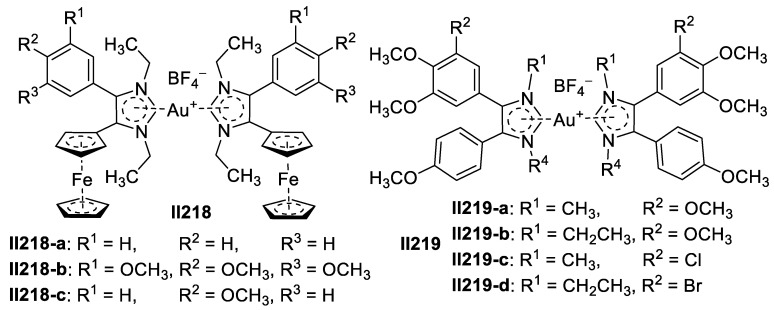



Gold *N*-heterocyclic carbene complexes have powerful biological effects, including anticancer and antiparasitic activities. A series of cationic gold (I) imidazole compounds with various 4,5-diarylimidazolylidene ligands were either prepared or repurposed for testing against protozoal *Leishmania major*, *Toxoplasma gondii*, and *Trypanosoma brucei parasites*. The ferrocene derivative **II218** showed distinctly higher activities against *Toxoplasma gondii* (EC_50_ = 0.013–0.046 μM) than the methoxyphenyl derivative **II219** (EC_50_ = 0.116–0.678 μM). The ferrocene **II218** also showed a reasonable selectivity for *Toxoplasma gondii cells* (best for **II218-a**, with a selectivity index [SI] = 28.1) [404].

Metronidazole is an essential antiprotozoan and antimicrobial agent with fine efficacy toward amebic colitis and trichomoniasis. Nonetheless, metronidazole tastes bitter, making it not so acceptable for young patients. By esterifying the hydroxyl group of metronidazole with benzoic acid, the produced benzoyl metronidazole **II220** could remove the weird odor but possessed poor hydrophilicity. If *β*-cyclodextrin joined in the modification, the inclusion compound of benzoyl metronidazole **II220** and *β*-cyclodextrin in a 1:1 ratio column significantly increased the solubility (from 5.22 × 10^−4^ M to 5.05 × 10^−3^ M). Consequently, *β*-cyclodextrin could be used as a solubilizer for benzoyl metronidazole, improving oral bioavailability [405,406].

Trichinosis is the most human-susceptible parasitic disease, in the forms of raw or undercooked meat with larvae in human food, which results in ailments. Albendazole (ABZ) and mebendazole (MBZ) are extensively employed in treating trichinosis such as *Trichinellosis*, *Echinococcis*, and *Neurocysticercosis*. However, their inappreciable curative effects remain with low hydrophilicity, low bioavailability, high doses, and a long treatment period. Trifluoromethyl benzimidazole analogue **II221** shared a similar parasitic activity with albendazole but was free from the first-pass effect due to the absence of the ester group in bio-oxidated albendazole. Unfortunately, poor hydrophilicity remained a limitation on pharmaceutical formulation and flexibility in administration. To deal with this, cyclodextrin could be introduced to act with compound **II221** to form inclusion complexes, among which 2-hydroxypropyl-*β*-cyclodextrin (HP-*β*-CD) performed the best. Surveys showed that not only enhanced solubility but also increased activities against juvenile and adult *Trichinellosis* were observed after forming inclusion complexes **II221**-HP-*β*-CD and ABZ-HP-*β*-CD. Subsequent research found that **II221**-HP-*β*-CD was more active than ABZ-HP-*β*-CD against adult *Trichinellosis*, which turned out to be the opposite when referring to juvenile *Trichinellosis* [407].

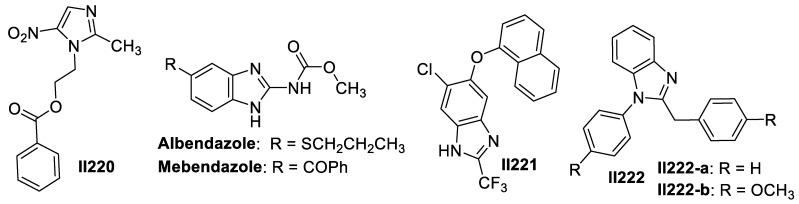



Based on benzimidazole being substituted by two aryl groups, four gold (I or III) complexes were newly prepared and subject to characterization and in vitro antiparasitic assays. In terms of leishmanicidal activity, free ligands **II222-a** and **II222-b** were ineffective, while, upon complexation, leishmanicidal activity was obtained in the four complexes. In particular, the complex formed by ligand **II222-b** and gold (III) ion was demonstrated to be the most effective toward *Leishmania* species in both stages of the parasite. This was especially true for *Leishmania braziliensis*, which was 21.75 times more potent than miltefosine, giving a corresponding IC_50_ value of 1.29 ± 0.22 μM. In aspect of this leishmanicidal activity, the IC_50_ values varied from 3.16 to 25.95 μM. Considering the macrophages that made up the majority of the host cells for leishmania, a selectivity index assay was also carried out, showing an apparent inclination for inhibiting parasitic components instead of macrophages, especially for the complex formed by ligand **II222-a** and Au (I) ion, which was almost 50 times more toxic for the parasite than for the macrophages. In addition to the leishmanicidal activity, all the complexes exhibited toxic effects against SK-Mel 103 and Balb/c 3T3 cancer cells [408].

### 2.5. Imidazole-Based Supermolecules as Antidiabetic Agents

With the improvement of living standards and the reduction in exercise, the incidence of diabetes is increasing year by year, gradually tending to affect younger people, with the incidence of children and adolescents rising significantly. Diabetes has become one of the greatest challenges facing science in the 21st century. It is a recognized multifactorial health problem that causes serious health complications, which is mainly associated with cardiovascular disease, kidney injury, and neuropathy, resulting in significantly high mortality [409]. This has attracted great attention worldwide, and much related work is devoted to research on and the development of hypoglycemic drugs. At present, the drugs that have been used in clinical settings, such as acarbose, metformin, and miglitol, have adverse side effects such as gastrointestinal discomfort, which hinder their application [410,411,412]. Therefore, finding new, more effective antidiabetic drugs, with fewer side effects and a lower cost, is still a fascinating research field. In this context, recent studies have found that imidazole-based heterocycle complexes are of great importance in mitigating insulin resistance and enhancing insulin secretion for their high bioavailabilities and weak drug interactions, thus opening up a new perspective for developing novel and highly active antidiabetic agents.

The enzyme protein tyrosine phosphatases (PTP) are known to be involved in the mechanism of glucose transport into the cell. Imidazole vanadium metal complex **II223**, as an inhibitor exclusively for PTP-1B, exhibited better potential in reducing serum glucose levels than metformin. It behaved as an inhibitor to suppress the overexpression of the PTP-1B enzyme, for which the inhibition was competitive [413].

The search for similar, newer antidiabetic complexes has become an interesting area of current biochemical research. As for the present tridentate Schiff base, no report on the structural characterization and its chelate with vanadium appear to have been reported. Two dioxidovanadium (V) complexes **II224a-b** incorporating tridentate ligand nicotinic acid and imidazoles were synthesized. Antidiabetic features such as α-amylase and α-glucosidase inhibition made them promising agents as inhibitors of insulin enzymes. Methylimidazole complex **II224-b** had low IC_50_ values toward the inhibition of α-glucosidase, which was similar to the control acarbose. It was, thus, revealed that imidazole complex **II224-a** showed the highest in vitro α-amylase (insulin-mimetic) activity among the compounds examined in this study, with an IC_50_ value of 23.669 [414].

The potential applicability of palladium complexes containing imidazole ligands for medicinal purposes prompted us to study their structure and biological activity. Palladium (II) complex **II225**, formed by two molecules 1,2-dimethylimidazole and one molecule of palladium chloride, demonstrated an IC_50_ value as low as 7.04 μg/mL, which was far less than that of acarbose (IC_50_ = 33.73 μg/mL). The reason for this might be associated with the specific configuration that was achieved after the complexation of palladium chloride with the different imidazole derivatives. The significant antidiabetic effects of the complexes would make them strong candidates for further testing against diabetes mellitus for future drug-discovery programmers [415,416].

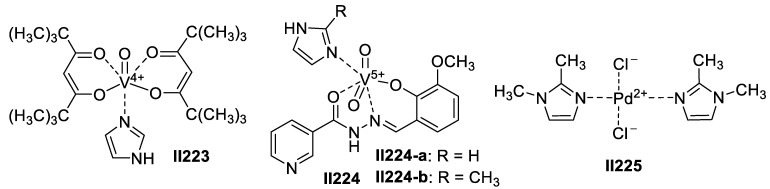



### 2.6. Imidazole-Based Supermolecules as Antihypertensive Agents

Hypertension is the most common cardiovascular disease and is the main factor to induce heart or renal failure as part of coronary disease. The pharmaceuticals used for the treatment of hypertension have a high requirement for their drug-release rate. For example, some drugs need to be released quickly, while others need to be released continuously to reduce the number of doses. Imidazole-based supermolecules and the involved inclusion complexes can fulfill the requirements above well, offering new options for developing or synthesizing hypertension drugs [417,418].

Angiotensin II causes vasoconstriction, elevated blood pressure, and, thus, hypertension. For the moment, oral drugs for hypertension, atherosclerotic lesions, and diabetes are mainly sartan drugs that could bind to subtype AT1 of the angiotensin II receptor. Antihypertensive drug telmisartan **II226** worked via its intensive interaction with the angiotensin II receptor. It was revealed in surveys that telmisartan maintained the same action mechanism after transforming into metallic complexes. Moreover, its Cu (II) complex possessed an enhanced antihypertensive effect [419].

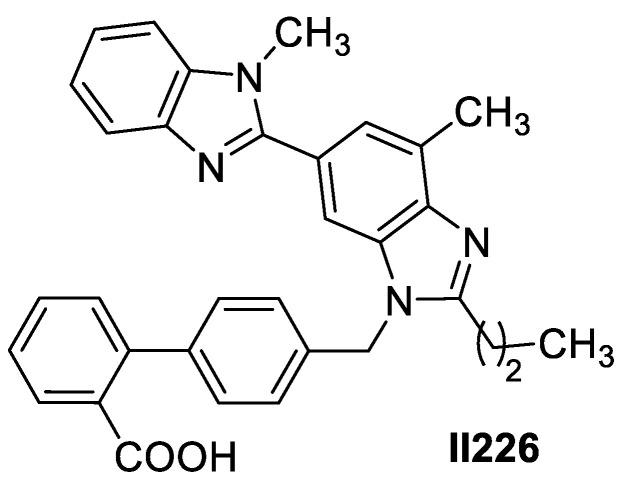



### 2.7. Imidazole-Based Supermolecules as Anti-Inflammatory Agents

Inflammation is one kind of basic pathological process induced by inflammatory-stimulating tissue lesions [420], which is mainly manifested as redness, swelling, pains, etc. Early widespread inflammatory drugs were glucocorticoid ones, which have gained remarkable achievement. However, the long-term use of such anti-inflammatory drugs produces dependence, and it is easy to cause adrenal cortical function decline and other side effects. With the appearance of drugs such as phenylbutazone, indomethacin, and ibuprofen, nonsteroidal anti-inflammatory drugs have attracted widespread concentration and inspired much work devoted to imidazole-based complexes in the field of inflammation treatment [421,422,423].

Superoxide dismutase (SOD) is a novel and important oxygen-free radical scavenger mainly used for inflammatory treatment, but the limitation of its wider usage exists because of its high cost of synthesis, large molecular weight, and low stability. Much work has confirmed that nonsteroidal anti-inflammatory drugs with carboxylic esters involved as ligands could form Cu (II) complexes with the activity of SOD. The nonsteroidal anti-inflammatory drug ibuprofen could generate Cu (II) complexes **II227** and **II228** with imidazole and guaranine, respectively. Inhibition toward the transformation of free oxygen radial was found in **II227** and **II228** treatments, with IC_50_ values of 0.70 μg/mL and 0.24 μg/mL, respectively, outperforming reference drug ibuprofen and rivaling or functioning less actively than natural SOD (IC_50_ = 0.70 μg/mL) [424].

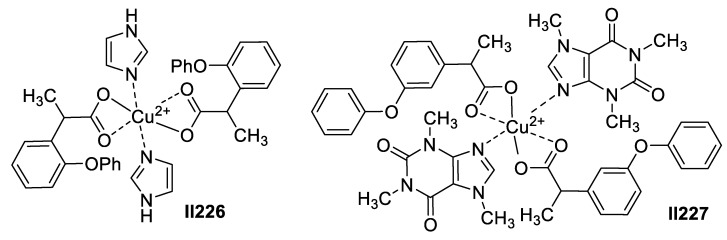



Ruthenium (II) complexes containing nitroimidazole derivatives have a wide range of application platforms, including anticancer, antimicrobial, insect repellant, and anti-inflammatory ones as well as other aspects of great development. For example, TNF-α, COX-2, and interleukins 1β and 6 are important mediators produced during the inflammatory response. Controlling the action of such mediators is the first step to reduce the deleterious effects of extensive inflammatory processes and avoid chronification. All concentrations (6–25 μM) of compound **II229** significantly inhibited TNF-α and IL-6 production by LPS-stimulated RAW 264.7 cells, similar to the dexamethasone reference standard, when compared to untreated LPS-stimulated cells. These findings demonstrated the significant antioxidant and anti-inflammatory properties of **II229**, showing activity levels comparable to those of corticosteroid drugs [425].

Copper (II) and zinc (II) ions are cofactors in the metabolic processes involved in collagen and bone metabolism, via articular/connective tissue and the immune system, and play a crucial role in prostaglandin synthesis. In particular, copper carboxylate drugs belong to an important class of anti-inflammatory and anticancer agents, and some of them are commercially available in clinical settings. Two new complexes **II230a–b**, of Cu (II) and Zn (II) ions with a tridentate –ONN′– Schiff base ligand, which was a bioactive scaffold derived from 2-aminobenzimidazole and 2-hydroxy-3-methoxybenzaldehyde, were synthesized. The results showed that Cu (II) complex **II230-a** (at 100 mg/kg) possessed potent anti-inflammatory activity, whereas Zn (II) complex **II230-b** (at 50 mg/kg and 100 mg/kg) exhibited significant analgesic activity, when compared to the standard drug (diclofenac sodium). All the complexes had apparently moderate and nearly akin antipyretic activity [426].

Organotin Schiff base complexes are endowed with extensive bioactivities, being greatly developed in their anticancer, antimicrobial, anthelmintic, anti-inflammatory, and other aspects. Imidazole ligand **II231** together with Sn (IV) ions could form a potent anti-inflammatory complex Ph_3_Sn. At the dosage of 20 mg/kg, the anti-inflammatory effect of Ph_3_Sn reaches 80% of that of the reference drug phenylbutazone. A toxicity test showed that the lethal median dose was over 400 mg/kg. Subsequent research found that phenyl-substituted complexes acted more actively toward inflammation than alkyl-substituted ones. Furthermore, trialkyl-substituted complexes were more active to treat inflammation than those with two alkyl groups substituted [427].

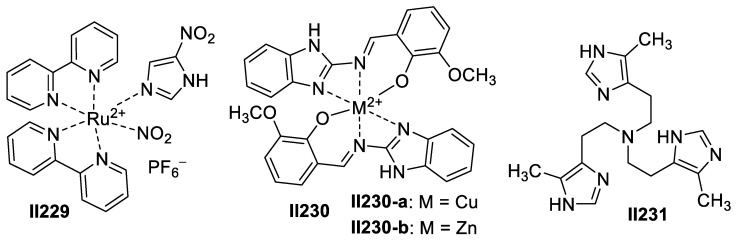



### 2.8. Imidazole-Based Supermolecules as Other Medicinal Agents

*Mycobacterium tuberculosis* (Mtb) is an intracellular pathogenic bacterium and the causative agent of tuberculosis. This disease is one of the most ancient and deadliest bacterial infections, as it poses major health, social, and economic challenges at a global level, primarily in low- and middle-income countries [428]. 

Thiosemicarbazides are biologically active, noncytotoxic organic compounds with proven antitumor, antiviral, antifungal, and antioxidant activities. Furthermore, the strong antibacterial potential of various thiosemicarbazide derivatives has also been documented. Among all the synthesized imidazole-thiosemicarbazides, the most potent compound was **II232**, with the estimated lowest minimal inhibitory concentration against *Mtb* growth, 15.62 μg/mL. The obtained results indicated that compound **II232** was replaced by a 4-dimethylaminophenyl group, which resulted in high antimycobacterial activity accompanied by low cytotoxicity toward host cells, the ability to inhibit *Mtb* intracellular growth, and the formation and maintenance of bacterial biofilms. Considering the importance of intracellular location and biofilm generation in bacterial survival and resistance to both host immunity and chemotherapy, the imidazole-thiosemicarbazide derivative **II232** appeared particularly promising as a target for further research aimed at the implementation of new, safe, and effective drugs in antitubercular regimens [429].

In different drug categories, triazoles and imidazoles are determined by their broad and effective activity as pharmacologically significant scaffolds [384]. In view of this, some new molecular entities of imidazole and 1,2,3-triazole pharmacophores were synthesized in a single molecular framework to explore the combined action of these two groups against *Mtb*. The antitubercular activity evidenced that compound **II233** was a potent antitubercular agent, with a MIC value of 1.47 μM [430].

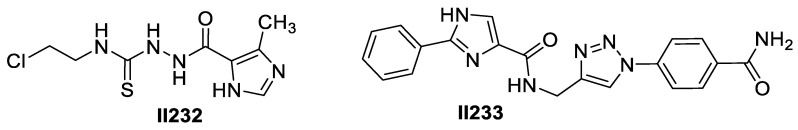



## 3. Imidazole-Based Supermolecules as Ion Receptors

The imidazole ring is a heterocyclic system, meaning its amphoteric nature can function as a cation or anion and even as a neutral organic molecule receptor [431]. The imidazole ring behaves as an excellent hydrogen bond donor moiety, capable of binding anionic species, while the presence of a donor pyridine-like nitrogen atom within the ring also converts imidazole derivatives into excellent metal ion receptors [432]. 

Imidazole-based receptors that selectively recognize ions have received more and more attention. In the past two decades, there have been many studies on the application of selective ion receptors in biology and medicine [433]. However, challenges remain in developing receptors that selectively bind ions. Significant progress has been made in the development of new ion receptors in recent years. The imidazole groups strongly interact with ions through noncovalent bonds such as hydrogen bonds and charge electrostatic interactions, so they are used as the basis of new receptors.

### 3.1. Imidazole-Based Supermolecules as Cation Receptors

Cations are undoubtedly among the crucial components of physiological processes, whereas cations also impact the normal operation of living creatures. One of the widely known metal species, mercury, is to blame for severe ailments such as kidney failure, Minamata disease, and other disorders. Similarly, the detection of silver ions is important not only for its indispensable role in photographic, electronic, and bactericidal applications but also for hazardous impairments such as the deactivation of sulfhydryl enzymes and the complexation to metabolites as accumulation grows [434,435,436]. All these situations call for an effective sensing technology for sensing cations with high selectivity and convenient observation.

The electron-rich imidazole ring endows its derivatives with the ability to interact with various cations. More encouragingly, imidazole-based complexes incorporated with electron-donating nitrogen atoms hold the promise of binding the positive-charged cations via electrostatic interaction, utilizing electrostatic interactions to provide a dramatic change in spectral properties when cations are added, thus distinguishing specific cations from others [437,438].

#### 3.1.1. Imidazole-Based Supermolecules as Cation Receptors for Iron Ions

Iron is one of the essential nutrients for the human body and plays an important role in metabolic processes, including oxygen transport, cell oxidation, DNA replication, and cell proliferation. Iron is an essential trace element in the human body, but excess iron can lead to poisoning. Thus, it is necessary to develop a simple, selective, and sensitive colorimetric sensor for iron ions [439].

In cation sensing, the colorimetric detection of Fe (II) was proven to be highly selectively detected, as is shown in the visible change from yellowish orange to red-violet over its competing cations (e.g., Mn (II), Fe (III), Co (II), Ni (II), Cu (II), Zn (II), Cd (II), and Hg (II) ions). The spectral profile of a 0.5-equivalent Fe (II) ion was also under investigation, in which complete quenching was achieved, and a new band centered at 275 nm was induced with an appreciable red shift from 468 nm to 572 nm in the UV-vis spectrum. A single conversion from compound **III1** to [**III1**-Fe (II)-**III1**] was also confirmed by absorption and emission titration [440].

Imidazole-involved terpyridyl Ru (II) complex **III2** was used to selectively detect Fe (II) ions. Iron (II), out of all the other bivalent cations with 3d electrons, was also sensed via Fe(dπ)→terpyridyl(π*), with acetonitrile as the solvent, exhibiting a new remarkable band centered at 572 nm in the absorption spectrum [441].

A Schiff base fluorescence sensor **III3** based on imidazole-coumarin derivative represented the selective “turn-off” fluorescence phenomenon caused by the paramagnetic quenching effect of Fe (III) ions, with the formation of 1:1 sensor–Fe (III) complexes in the DMF/HEPES aqueous buffer (7/3, *v*/*v*, 10 mM, pH = 7.4). Although free **III3** exhibited distinctive pH responses, this approach could be applied to detect Fe (III) ions in most biological systems. Furthermore, the detection limits of **III3** for Fe (III) ions were calculated by the fluorescence titration to be 0.83 μM, and the determination of Fe (III) ions’ concentration in the real sample waters was applied successfully [439].

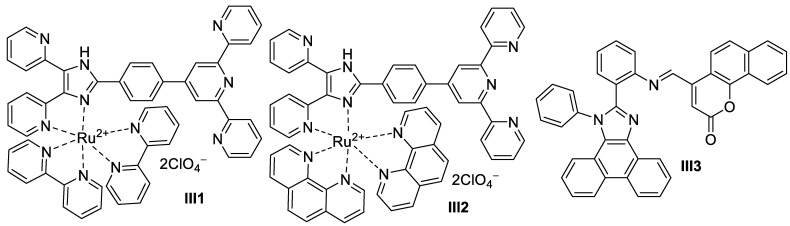



#### 3.1.2. Imidazole-Based Supermolecules as Cation Receptors for Copper Ions

Copper is a potential metal-based pollutant as well. Hence, its prodigal use in chemistry, biosciences, medicine, and biotechnology is not judicious. Copper ions are also toxic to microorganisms even at submolar to micromolar concentrations. Thus, the design as well as the synthesis of a highly sensitive yet selective chemosensor for copper ions is of current research interest.

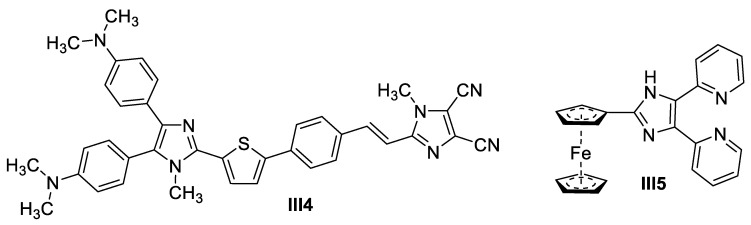



A highly conjugated biimidazole push–pull dye **III4** could simultaneously detect Cu (II) and Hg (II) ions in an aqueous environment. Compound **III4** was inclined to coordinate with Hg (II) ions via the thiophene residue and inhibited the charge-transfer (CT) process, leading to an apparent color change from a yellow-colored solution to a colorless one. In addition, when Cu (II) ions were added, an unusually large bathochromic shift phenomenon was observed, accompanied by a change in color from yellow to red. This result accounted for the formation of the 6-imino-5,6-dihydropyrrolo[3,4-d] imidazole-4(3H)-one (IPIMO) derivative with the catalysis of the Cu (II) ions, which strongly affected the charge-transfer state of the compound as well as its polarizability [442].

A pyridine arm-modified imidazole compound **III5** was a highly selective fluorescent “turn-on” receptor for the Cu (II) ions. In addition, the composition of **III5**–Al (III) and **III5**–Cu (II) systems could detect Br^−^ and I^−^ through color change. Importantly, receptor **III5** was successfully used to detect Al (III) ions in living A549 cells [443].

The bifunctional probe of 2-(1*H*-imidazol-2-yl)-1H-phenol[9,10-d] imidazole **III6** was used as a ratio-sensitive sensor. It was found that receptor **III6** showed good recognition ability for Cu (II) ions. According to Job’s diagram, the binding stoichiometry of a receptor with a cation complex was 1:1. The density functional theory results clearly demonstrated that the binding modes and the properties of these receptors could be used as selective fluorescent sensors for analytes with the ratiometric response and found potential applications in biological systems [444]. 

A trisubstituted imidazole-based colorimetric and fluorogenic chemosensor **III7**, for the detection of Cu (II) ions and subsequent colorimetric detection of an amino acid, cysteine, was investigated. Chemosensor **III7** exhibited a distinct color change from colorless to red in the presence of Cu (II) ions in an aqueous medium. The **III7**–Cu (II) complex could also be used to detect cysteine with the naked eye over a series of amino acids. The receptor **III7** behaved as a highly selective colorimetric and fluorescent sensor for Cu (II) ions at concentrations as low as 4.33 and 2.25 μM, respectively. These values were much less compared to the WHO’s recommended limit of 30 μM for Cu (II) ions in drinking water [445].

A highly selective Ru (II)-polypyridyl-based chemosensor **III8** has been developed for the rapid analysis of Cu (II) ions in aqueous solution and on paper strips. Complex **III8** displayed excellent fluorescence quenching when binding with Cu (II) ions, and the limit of detection was computed to be 50.67 nM, which was much lower than the allowable Cu (II) ion standard (~20 μM) in drinking water. Interestingly, Cu (II) ion sensing was found to be reversible with EDTA addition [446].

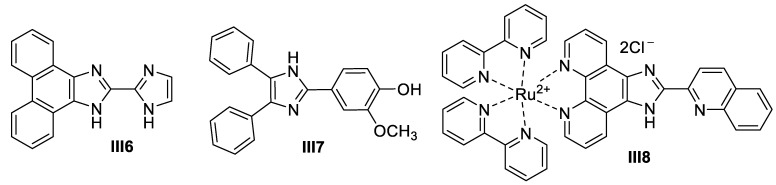



#### 3.1.3. Imidazole-Based Supermolecules as Cation Receptors for Zinc Ions

Zinc has important effects on human health, and it is crucial to maintain an optimum level of zinc ions (0.9 ppm in the blood) for homeostasis in the human body. This necessitates the easy detection of zinc in the blood.

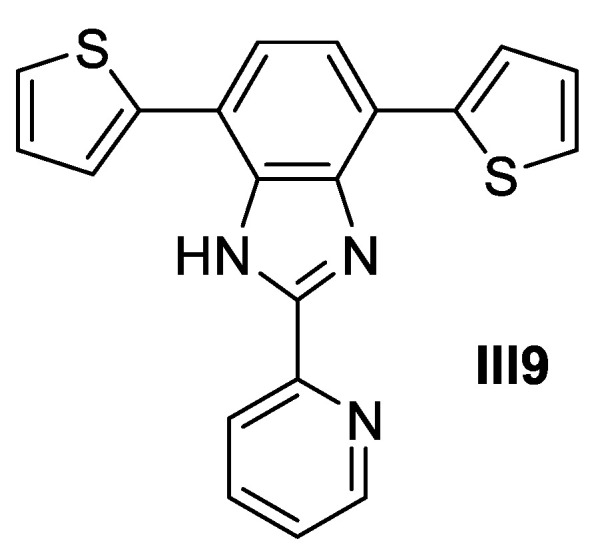



Benzimidazole-based sensor **III9**, which could be used to detect zinc ions and form the Zn (II) complex, was synthesized and was found to be highly selective as a fluorogenic chemosensor and in the solid-state sensor mode for the detection of Zn (II) ions. Other metal ions did not show any appreciable change in the fluorescence emission intensity of receptor **III9**. The lower limit of detection for Zn (II) ions was found to be 16 nM, which could be helpful in the detection of trace levels of Zn (II) ions in any sample [447].

#### 3.1.4. Imidazole-Based Supermolecules as Cation Receptors for Mercury Ions

It is well-known that mercury can be absorbed through the skin, mucous membrane, and respiratory system and cause serious health problems including DNA impairment, dysfunction of the liver and kidney, and permanent lesions of the central nervous system. Hence, development of simple, selective, and sensitive colorimetric sensors for Hg (II) ions in aqueous solution is very much necessary, as they would be required for the rapid and on-site detection of this metal ion. 

A quinone-diimidazole ensemble **III10** exhibited an instantaneous and striking color change, selectively and sensitively, from brown to blue with Hg (II) ions in DMF/water (1/9, *v*/*v*). Compound **III10** with one molecule of mercury ions could form a complex via the nitrogen on imidazole. The addition of Hg (II) ions to **III10** led to fluorescence quenching with a limit of detection of 10 nM [448].

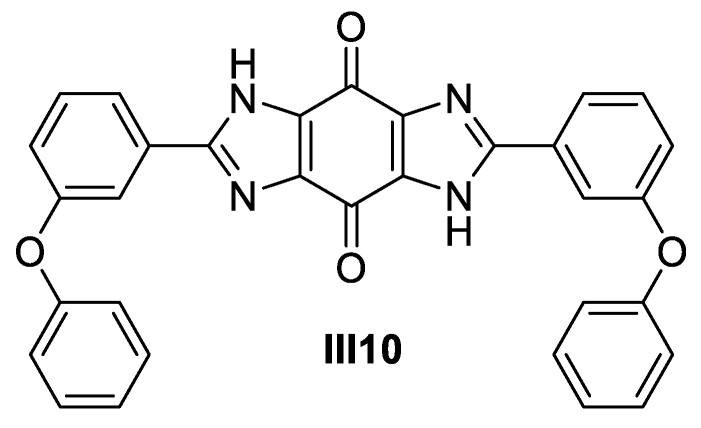



### 3.2. Imidazole-Based Supermolecules as Anion Receptors

The ubiquitous anions have long been recognized to be crucial players in substantial areas, especially multidisciplinary applications. The fluorine ion works in caries prevention, the treatment of osteoporosis, and other aspects; carboxylate anions are greatly required in numerous parts of metabolism and food storage; the acetate groups of human proteins act as molecular switches in the inheritance of DNA, cell division, and cell aging; and phosphoric acid and its derivatives also play an important role in bioenergy storage and signal transduction. Nevertheless, an excess number of anions is not favorable. For instance, excess phosphates tend to account for the eutrophication that poses a severe threat to aquatic life. To meet the demand for eco-friendly industrialization, insights into anion receptors endowed with an anion-binding nature are necessary [449].

Imidazole-based complexes might open up a new yield for artificial anion receptors. The research on imidazolium salts as anion receptors began nearly 20 years ago. Imidazolium groups have been used as the basis of the new receptors because they strongly interact with anions through (C-H)^+^ꞏꞏꞏ X^−^-type ionic hydrogen bonds and charge-charge electrostatic interactions [450]. Since imidazole groups can bind anions in aqueous solutions, their derivatives with different topological characteristics are widely used for sensing various anions. In the recent years, the development of imidazole receptors has made new important contributions, such as the combination of imidazole and halogen bonds, the expansion of imidazole coupling systems, and the creation of new scaffolds. Some imidazole-derived fluorescent probes have been used to detect phosphate-containing anions, such as ATP, GTP, DNA, and RNA.

Imidazole is an essential player in anion sensing. Apart from the stable configuration of the complex, the electron-withdrawing capability of naphthoquinone renders naphthoquinone-imidazole a better hydrogen bond donor as well as a more sensitive respondent in the altered environment if a hydrogen bond is formed. Complex **III11** was an instant fluoride sensor for colorimetric detection with the naphthoquinone-imidazole ligand, Ni (II), and Cl^−^ ions in a 1:1:2 stoichiometry. Even if the center ion was replaced with Zn (II) ions of the identical stoichiometry, the complex could also respond to cyanide, exhibiting visible colorimetric change from yellow to red with the naked eye. The promising application for the immediate detection of F^−^ and CN^−^ ions is also in progress [451].

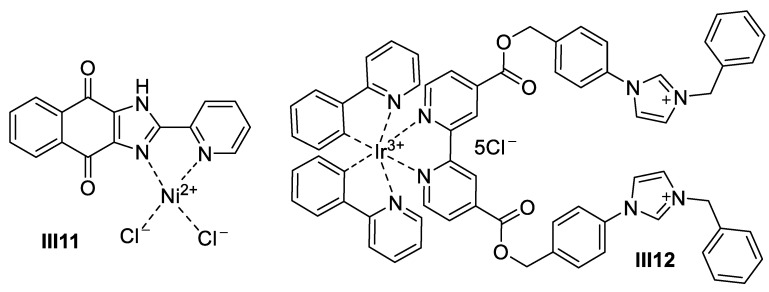



Iridium (III) complexes are conferred with a long-lived excited state, a high quantum yield in phosphorescence, and variable photophysical properties in addition to multi-mode coordination with diverse ligands [452,453,454]. Here, two imidazolium-like groups were incorporated into an Ir (III) complex to increase solubility and positive charge. This kind of turn-on sensor **III12** for ClO_4_^−^ ions was also designed utilizing aggregation-induced emission (AIE). In the study, a record-breaking 250-fold intensification in the red emission signal ensued from the addition of an aqueous solution of ClO_4_^−^ ions. As verified by transmission electron microscope, scanning electron microscope, confocal laser scanning microscope, and dynamic light scattering, nanoparticles form owing to the aggregation induced by ClO_4_^−^ ions [455,456].

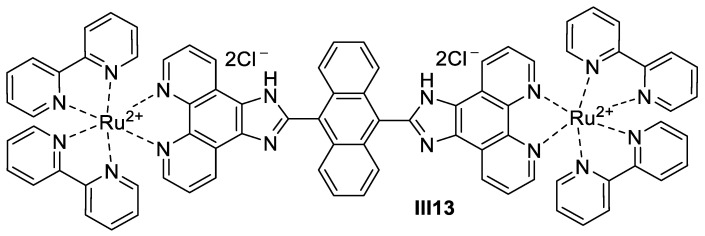



An anthryl moiety-containing dinuclear Ru (II) complex **III13** was under investigation. The highly selective and sensitive detection of singlet oxygen was achieved by means of luminescent observation at 458 nm wavelength with a 5.2- and 7.6-times stronger signal than the initial emission ones tested in the neutral and alkaline solvents, respectively. Owing to its hydrophilic property, low limit of detection (2.7–3.1 nM), and easily obtained visible light excitation, it was of great potential to optically sense ^1^O_2_ singlet oxygen [457].

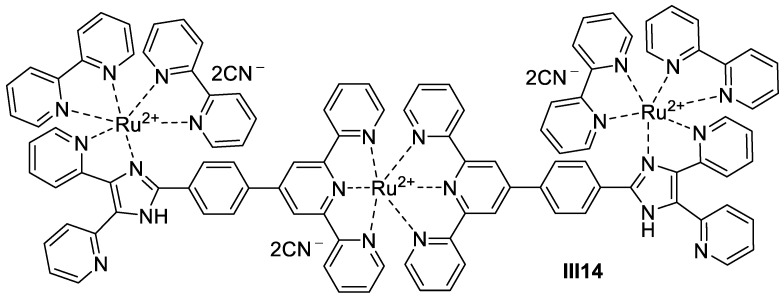



The anion-sensitive photophysical responses of a new class of Ru (II) ions, the luminescent homo- and heterotrimetallic complex **III14** that is derived from heteroditopic bipyridine-terpyridine bridging ligands, are used to develop their applications as the anion probes of choice. The design strategy provided two acidic imidazole NH protons in the secondary coordination sphere of all complexes, and interaction between the polarized NH moieties and the anions alter the ground and excited state behaviors of the triads. The triad functioned as chemosensors for the F^−^, CN^−^, AcO^−^, and H_2_PO_4_^−^ ions in MeCN but behaved as selective sensors for CN^−^ and SCN^−^ ions in water, with a very low detection limit of as low as 10^−8^ M. Furthermore, complex **III14** was capable of discriminating CN^−^ and SCN^−^ ions in water by taking advantage of its varied extent of emission spectral responses [458].

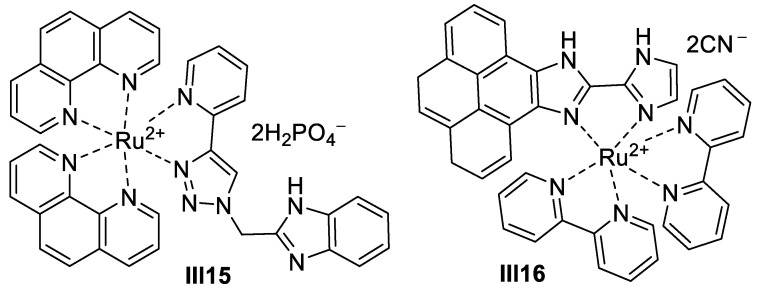



Ruthenium-centered complex **III15**, with a benzimidazole-appended triazole as the ligand, was also designed for anion recognition as a luminescent probe. Here, cation sensing could also be achieved by the hydrogen binding sites offered by the introduced benzimidazole moieties. The probe worked in pure acetonitrile, with an obvious incremental sensitivity toward O_7_P_2_^4−^ and H_2_PO_4_^−^ ions compared to the probe-free system, displaying almost a 10-fold increase in photoluminescent intensity, 583 nm. The association constant was provided to be 3.3 × 10^3^ M^−1^ and 6.8 × 10^3^ M^−1^ for the O_7_P_2_^4−^ and H_2_PO_4_^2−^ ions, and the low detection limits were also obtained, 5.73 and 5.19 ppb, respectively [459].

In the realm of mimicking molecular traffic signals, polypyridyl–imidazole-incorporated Ru (II) complex **III16** showed great potential in the optical recognition for the successive addition of CN^−^, H_2_PO_4_^−^, and H^+^ ions, offering a desirable insight into logic gates in addition to memory devices. The mechanism underlying the recognition lay in the polypyridyl-imidazole group being endowed with photophysical properties including the reversible protonation and deprotonation of the NH groups. Among the ions detected, F^−^ as well as H^+^ ions were proven to be well stimulation for this kind of imidazole-based complex to be adoptable in memorizing devices for their reversible logic functions, with the aid of their new absorption signal centered at 540 nm. High selectivity was obtained only in the presence of CN^−^ ions, in stark contrast to the F^−^, AcO^−^, and H_2_PO_4_^−^ ions [460].

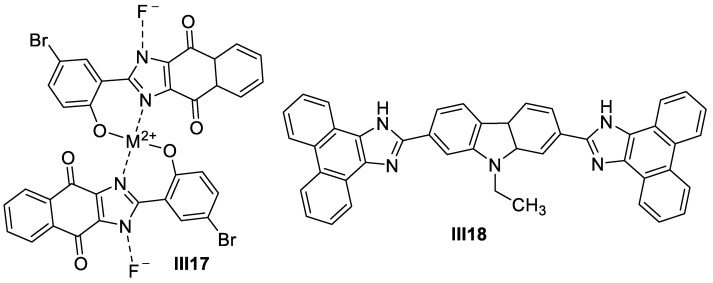



Among various anions, the colorimetric detection of fluoride ions in an aqueous solution is essential. Hence, it is important to develop highly selective, sensitive, and rapid colorimetric sensors for the detection of fluoride ions in aqueous solutions. 

A quinone-imidazole ensemble copper metal complex **III17** sensed fluoride ions in 80% DMF solution with an instantaneous color change from yellow to red. The fluoride ions’ sensing was through the formation of a hydrogen bond between the imidazole N-H group and fluoride ion, leading to a strong receptor–fluoride ion complex with binding constants in the order of 10^7^ M^−1^. The coordination of metal ions with the quinone–imidazole ensemble was found to increase the hydrogen bond donor property of the N-H group toward fluoride ions [461].

Coincidentally, a phenanthrol (9,10-d)imidazole-based colorimetric receptor **III18** (CPH) detected fluoride ions by a change in fluorescence color from blue to green and differentiated the fluoride ions from the other competing anions. The **III18** F^−^ complex formed during the sensing was reversible by treating it with the acid, so the receptor **III18** could be reusable [462]. 

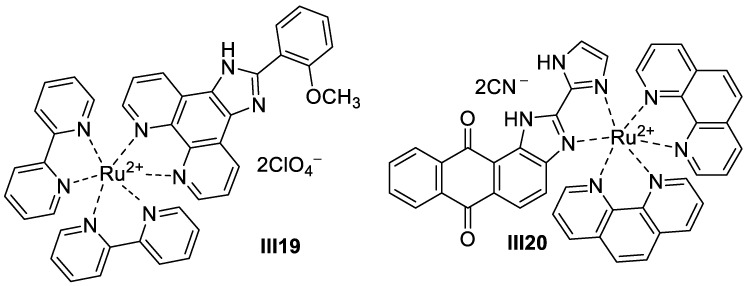



A receptor based on imidazole phenanthroline and its Ru (II) polypyridyl complexes possessing the imidazole NH have been reported as anion sensors, the majority of them possessing electron-withdrawing substituents. The present results displayed an extreme “On-Off” effect in the Ru (II) complex **III19** formed by methoxyphenyl-substituted imidazole phenanthroline and two bipyridines. The introduction of the electron-withdrawing metal center drastically changed the recognition behavior of the imidazole phenanthroline receptor toward anions and increased the acidity of the imidazole N-H to serve as the binding site for the added anions. The fluorescence intensity of complex **III19** was quenched upon the addition of basic anions such as AcO^−^, CN^−^, F^−^, H_2_PO_4_^−^, and SO_4_^2−^ ions [463].

Anthraquinone-biimidazole-based ruthenium (II) complex **III20**, which had good photophysical and electrochemical properties, was capable of responding to selected anionic inputs and mimicking the functions of several molecular logic devices. The covalent linking of the electron-accepting 9,10-anthraquinone moiety to the biimidazole moiety, followed by its coordination to the Ru (II) center, led to the enhancement of the acidity of the imidazole NH protons in the complexes, which in turn were utilized for interaction with selected anions either via hydrogen bonding interactions or by completing a proton transfer. The complex **III20** acted as a multi-channel sensor for the F^−^, CN^−^, and AcO^−^ ions in an organic medium and as highly selective sensors for the CN^−^ ions in an aqueous medium with an extremely low detection limit [464].

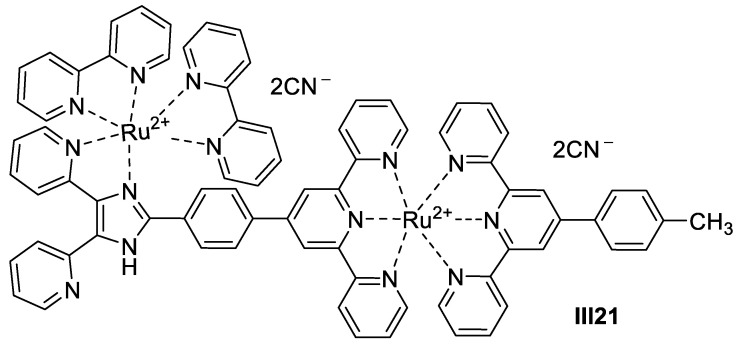



A bimetallic Ru (II) complex **III21** derived from a heteroditopic bridging ligand consisting of a bipyridine chelating site covalently connected via a phenyl–imidazole spacer exhibited very high selectivity and sensitivity toward CN^−^ ions in water, with a very low detection limit of up to 10^−8^ M. The acidic NH protons in the complex interacted with basic anions either through hydrogen bonding or through a complete proton transfer. By virtue of its rich and versatile photophysical properties, the bimetallic Ru (II) complex offered multiple optical channels. Thus, by changing the tridentate terminal ligand in the asymmetric bimetallic Ru (II) complexes, the mode of luminescence sensing toward CN^−^ ion was reversed [465].

## 4. Imidazole-Based Supermolecules as Imaging Agents

Imaging techniques can be used to generate high-resolution images of cells and tissues with real-time data acquisition, which have many important potential applications in radiation-drug development and surgical planning and guidance [466]. Imidazoles with nitrogen atoms as coordination sites are of great potential in chelation, especially in the chelation of molecules that serves as an indicator of cell condition. Moreover, the nitrogen-involved aromatic rings of imidazoles also enable them to afford a variety of noncovalent interactions such as C-H···N, π-π stacking, and C-H···π interaction, with a modification that leads to flexible identification or selectivity toward specific targets. Imidazole participates in the formation of ligands or supermolecules that can be targeted to label cells or to track intracellular materials by fluorescence for imaging and more sensible monitoring. 

### 4.1. Imidazole-Based Supermolecules as Imaging Agents for Cells

Imidazole-based supermolecules can be used for imaging cells or organelles because they are radioactive or can be highly specific when accumulating in cells. The resultant complexes can accumulate in cells with high specificity, targeting labeled cells and enabling cell imaging.

Supported by visible-light-absorbing C^N and bis-NHC ligands incorporating pyrazole rings, iridium (III) complex **IV1** was engaged in anticancer applications (IC_50_ values from 0.5 to 56.1 μM toward HeLa cells) and the bioimaging of cancerous cells due to its long-lived excited states and high quantum yields. Green or yellow fluorescence was given in cytoplasm, or, more exactly, mostly in the endoplasmic reticulum. It is well worth mentioning that complex **IV1** could highly specifically accumulate in the endoplasmic reticulum (ER) of cancer cells. The induced ER stress and immunogenic cell death were assumed to account for the cytotoxicity of the prepared complexes [467].

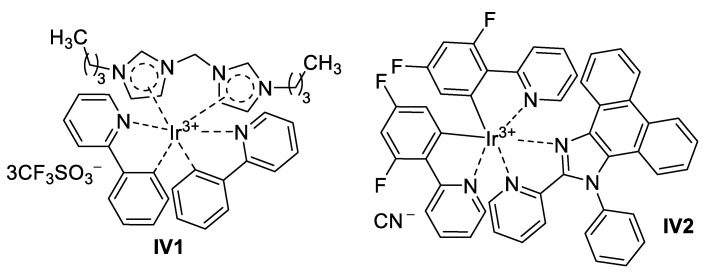



A mitochondrion is an essential organelle acting as a powerhouse. Consequently, efforts have been undertaken to target mitochondria for cancer therapy. Making use of a two-proton emission property, an imidazole-incorporated Ir (III) complex **IV2** was devised to tackle the photobleaching of photosensitizers (PSs). This complex aggregated in water because of its lipophilicity and was successfully directed to mitochondria in monolayer cells and multicellular spheroids, providing sequentially enhanced ^1^O_2_ quantum yields and exhibiting favorable photostability and two-photon photodynamic therapy (PDT) properties. In the form of aggregation, the prepared complex held appealing promise for organelle-targeted PDT applications [468].

Technetium-99m (^99m^Tc) is an important radionuclide with wide applications in diagnostic nuclear medicine. ^99m^Tc radiopharmaceuticals are routinely used for the diagnosis of various medical conditions, by imaging organ function (myocardial/brain perfusion, etc.) or by targeting the specific biological structures (cells and receptors) involved in disease. During the past few years, efforts have emerged to develop radioactive tracers based on ^99m^Tc-tricarbonyl nuclei to target nuclear DNA for cancer imaging or radiotherapy. Labeling strategies with the ^99m^Tc-tricarbonyl core involve the use of tridentate chelators or the combination of a bidentate chelating and a monodentate ligand; the latter is known as the [2 + 1] approach.

The ^99m^Tc-labeled imidazole derivatives were identified as potential radiotracers for hypoxic tumor imaging, which was considered very useful because of their non-invasive detection capabilities. Biological distribution and imaging studies showed that 2-mercaptobenzimidazole-substituted ^99m^Tc-tricarbonyl complex **IV3** had significant tumor absorption and a high target/non-target ratio (T/B, T/M), suggesting that complex **IV3** might be a potential tracer for the early diagnosis of hypoxic tumors [469].

The ^99m^Tc-labeled imidazole substituted quinolone tracer **IV4** was prepared in high yield and was able to enter K-562 human erythroleukemia cells. Most of the radioactivity was found in the cytoplasmic and mitochondrial fractions. These results suggested that the distribution of ^99m^Tc tracers might be related to the binding of intracellular targets such as mitochondrial DNA or the DNA–topoisomerase complexes. In addition, the biodistribution results showed that the [2 + 1] ^99m^Tc-tricarcarbonyl quinolone complex had appropriate pharmacokinetic characteristics, and its value as an imaging agent could be further evaluated in future studies [470].

For radiolabeled bisphosphonates, the ^99m^Tc-methylenediphosphonic acid was used in the imaging of calcium turnover and bone damage. By connecting the central metal ion through the imidazole linker and adding the bisphosphonate to complex, the high yield of ^99m^Tc-tricarbonyl imidazole-alendronate derivative **IV5** was obtained. The in vitro stability and specific localization of complex **IV5** was observed in the bone-metabolism-active shoulder (7.9 ± 0.2% ID/g) and knees (15.1 ± 0.9% ID/g), after six hours of administration [466].

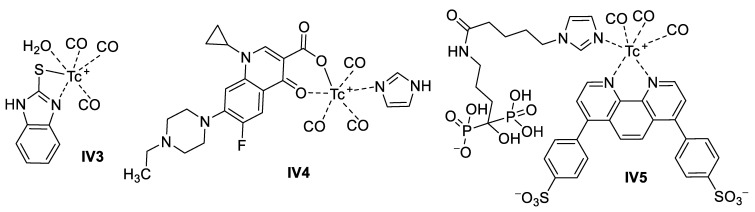



### 4.2. Imidazole-Based Supermolecules as Imaging Agents for Intracellular Materials

The designed and synthesized imidazole ligands can detect and bind intracellular ions to form complexes, which have a highly selective and sensitive fluorescence response, enabling the tracking of intracellular ions by fluorescence microscopy to achieve cell imaging.

Due to its potential in fluorescent and chelating aspects, benzimidazole-based Schiff base (BMSA) **IV6** was applied for the selective fluorescent sensing of the Al (III) and Cu (II) ions in HeLa cells, providing limits of detection at 0.31 μM and 0.54 μM, respectively. Upon the addition of the two cations, red-shift and significant quenching took place in the case of the Al (III) and Cu (II) ions, respectively [471]. 

Pyrazole-derived imidazoles were designed and synthesized for sensing Al (III) and Fe (III) ions. Sensor **IV7** showed a highly selective and sensitive fluorescent response with the addition of Al (III)/Fe (III) ions in an acetonitrile-water mixture. The strong fluorescent molecule exhibited a notable radiometric emission at 462 nm and 470 nm for the Al (III) and Fe (III) ions, respectively (λ_ex_ = 280 nm). The limit of detection of **IV7** with the Al (III)/Fe (III) ions was calculated as 2.12 × 10^−7^ M and 1.73 × 10^−6^ M, respectively. The probe was used to track Al (III) and Fe (III) ions in cancer cells via fluorescence microscopy. In addition, the sensor was practically suited for the bio-imaging studies of the Al (III) and Fe (III) ions in HeLa cells. This sensor showed practical applications in real sample analyses [472].

The phenylene [9,10-*d*] imidazole-coumarin derivative **IV8** based on an Fe (III) fluorescence chemical sensor showed a selective and sensitive off fluorescence reaction by forming a 1:1 **IV8**-Fe (III) complex, which could be used for the determination of Fe (III) ions in real water samples. The quenched fluorescence system of the **IV8**-Fe (III) complex was able to recognize phosphate anions in the aqueous solution by a displacement strategy with fluorescence recovery. Complex **IV8** was successfully applied for the determination of PO_4_^3−^ ions in the imaging of live Hep G2 cells. Therefore, this chemo-sensor is not only a powerful tool for detecting phosphate anions in aqueous solutions and living cells but also facilitates the development of other anionic fluorescence chemo-sensors [473].

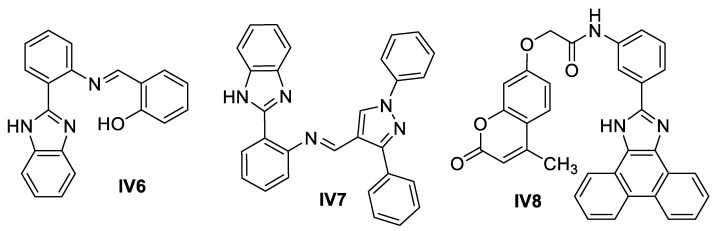



## 5. Imidazole-Based Supermolecules as Pathological Probes

Abnormal levels of elements or molecules in organisms have long been taken into consideration for devising pathological probes. For instance, the fluorescent molecular probe has emerged as an attractive tool for the detection of various chemical and biological components, such as thiols and amino acids, because of its simplicity, low cost, sensitivity, fast response time, and application not only in in vitro assays but also in in vivo imaging studies.

An imidazole ring has been frequently incorporated into a fluorescent skeleton to provide artificial molecules that are suitable for the selective recognition of the ions and biological molecules in the human body, which have manifested the potent capacity of imidazoles as pathologic probes [17]. Imidazole-based supermolecules have been documented as having capabilities of sensing pathological biomarkers. Rich electronic properties, multiple coordination sites, and various intermolecular interactions are all favorable in the further exploitation of imidazole-based supermolecules as pathological probes.

### 5.1. Imidazole-Based Supermolecules as Pathological Probes toward Organelles 

Imidazole-based supermolecules can be aggregated into organelles or detect specific substances in organelles to be used as pathologic probes to diagnose diseases. The more relevant studies are on cell membranes and lysosomes.

Boron pyridyl imidazole complex **V1**, involving the twisted intra-molecular charge transfer (TICT) characteristic, was investigated to probe BSA. Complex **V1** was demonstrated to be an efficient fluorescence probe to discriminate BSA from other proteins such as HSA. The encapsulation of complex **V1** in the hydrophobic subdomain IIA of BSA inhibited the TICT state and resulted in the 70-fold emission enhancement of complex **V1**. In addition, it was found that complex **V1** could distinguish denatured BSA from native BSA, due to the difference of at least a 15 nm emission wavelength. The tracking of the BSA denaturing process was easily achieved, through the change in the complex **V1** emission wavelength from 485 to 465 nm. Thus, a promising pharmacological dual-functional probe was provided for both the selective recognition of BSA and the identification of denatured BSA from the native BSA [474].

The development of an optical tumor imaging probe with minimum background noise is of great significance for the early detection of small lesions and the accurate diagnosis of tumors. Lysosomes are organelles that break down proteins, nucleic acids, polysaccharides, and other biological macromolecules. The pH of the lysosome in cancer cells can be lower than 4.5, with high Na^+^/H^+^ exchange activity. It is an ideal target intracellular organelle for cancer diagnosis, according to pH changes. The boron pyridyl imidazole complex **V1** had the ability to show a pH-activatable “Off-On” fluorescent switch by inhibiting twisted intramolecular charge transfer upon protonation at a pH of 3.8–4.5. Complex **V1** specifically emitted green fluorescence in the lysosome of cancer cells, indicating that it has a good cancer-cell-specific imaging ability [475].

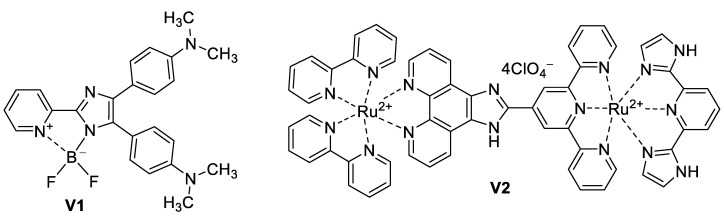



The dinuclear Ru (II) complex **V2** was found to be capable of differentiating live HeLa cells from healthy HEK293 cells, by selectively accumulating in the lysosomes of the HeLa cells. It had the advantages of a large Stokes shift, low cytotoxicity, and single- and two-photon excitation near the infrared emission. Such a ruthenium complex has great potential to become an ideal lysosome-specific probe [476].

Lipopolysaccharide (LPS) endotoxin was common in the outer cell membrane of Gram-negative bacteria, in contrast to that of Gram-positive bacteria, with a thick layer of peptidoglycan. Utilizing the luminescent profiles derived from Pt···Pt and π···π stacking, the platinum (II) complex **V3** formed by dibenzimidazole-substituted pyridine and the hexamethylene glycol methyl ether groups was capable of discriminating Gram-negative bacteria from Gram-positive ones. The Pt (II) complex, which drove aggregation by electrostatic interaction with LPS in Gram-negative bacteria, produced a unique fluorescence due to its heavy metal ligand charge transfer and had a low detection limit of 5.7 nM [477].

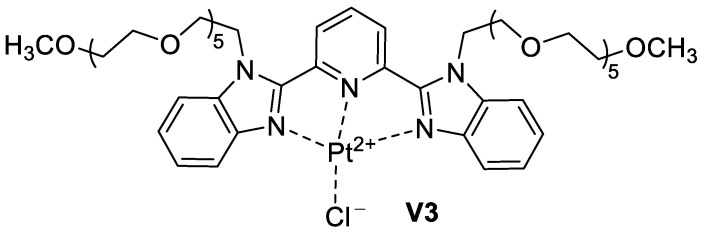



### 5.2. Imidazole-Based Supermolecules as Pathological Probes toward the Detection of Biological Active Substances

The biological body contains a variety of active substances that can affect the phenomena of life, such as nucleic acid, mineral elements, etc. Therefore, it is urgent to find a probe that can detect these substances quickly and easily. Imidazole-based supermolecules can be used as pathologic probes for the detection of biologically active substances. The imidazole ligand itself can be used as a probe to form a complex with metal ions and can also form a probe with metal ions for detection, to achieve the purpose of substance detection.

#### 5.2.1. Imidazole-Based Supermolecules as Probes to Detect Biological Mercaptans

Biological mercaptans play a key role in many cellular functions. Abnormal levels of biological mercaptans are considered to be signs of many diseases. As a result, fluorescent probes for biological mercaptans have been developed [478].

Diphenyl-substituted imidazole derivative **V4** formed a highly colored and non-emissive 1/1 stoichiometry complex with Cu (II) ions in water–acetonitrile 1/1 (*v*/*v*) solutions. This **V4**-Cu (II) complex exhibited unique selectivity and sensitivity for glutathione (GSH) detection in aqueous environments. When GSH was added to the aqueous (pH 7.4)–acetonitrile (1/1, *v*/*v*) solution of the **V4**-Cu (II) complex, the color was obviously bleached, and a strong emission band appeared. This was because the GSH-induced dementalization of the **V4**-Cu (II) complex allowed the free probe to regenerate. The reaction to glutathione was quite selective because other bio-thiols tested (Cysteine and Homocysteine) were unable to induce any color or emission changes [479].

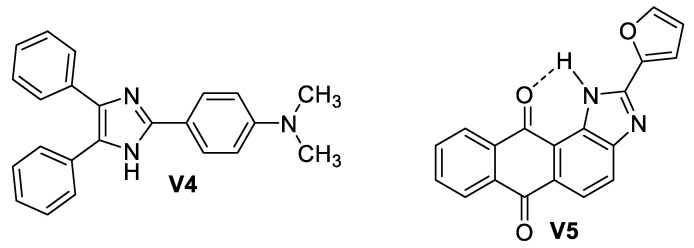



Anthraquinone and its derivatives had been used to construct chemosensors owing to their excellent properties such as high absorption coefficients, excitation and emission with the visible wavelength region, and low toxicity. A simple anthraquinone–imidazole-based compound **V5** was synthesized as an effective colorimetric and fluorometric Ag (I) sensor. Compound **V5** exhibited high sensitivity to Ag (I) ions in a linear dynamic range of 0.8–32 μM, with a detection limit of 66 nM. The **V5**-Ag (I) complex, which was prepared “in situ” based on the strategy of metal displacement, was used as a reversible sensor to detect the biological mercaptans (Cysteine, Homocysteine, and Glutathione) through the complexation reaction. The detection limits of cysteine, homocysteine, and glutathione were 89 nM, 174 nM, and 208 nM, respectively. As an “on off on” sensor of Ag (I) ions and biological mercaptan, compound **V5** showed good recoverability and was recycled up to four times. In addition, compound **V5** with low cytotoxicity was efficient in monitoring Ag (I) ions and biological mercaptan in living human hepatoma cells SMMC-7721 [480].

#### 5.2.2. Imidazole-Based Supermolecules as Probes to Detect Adenine

Adenine as a crucial component of nucleic acid has been recognized as an indication of whether there exists disorder in organisms. For instance, common diseases such as epilepsy, cancer, mental retardation, HIV infection, and Parkinson’s disease all can incur an abnormal level of adenine. 

The adenine fluorescent sensor formed by Eu (III) ions and bis(guanine) ligand **V6** was found as a promising candidate for a pathological detector with a high selectivity out of structurally similar guanine, xanthine, hypoxanthine, and uric acid. The exclusive selection was ascribed to π···π stacking for its ends that both bear similar structures to the detected adenine. Its low detection limit was computed to be around 4.70 × 10^−7^ mol/L [481].

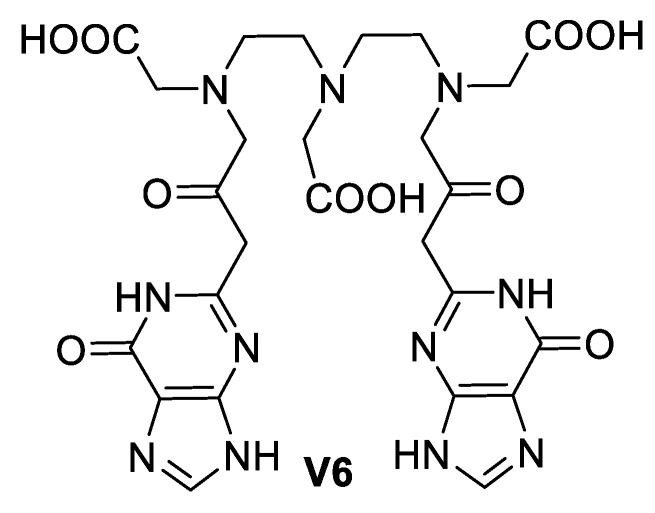



#### 5.2.3. Imidazole-Based Supermolecules as Probes to Detect Metal Ions In Vivo

A part of metal ions are essential trace elements of human health, but excessive metal ions are harmful to human health [482]. Most of the metal ions are carcinogens and lead to serious health concerns by producing free radicals. Hence, the fast and accurate detection of metal ions has become a critical issue [483].

Potential fluorescent probe **V7**, which contained a conjugated imidazole–pyridine structure, was rationally developed for Ag (I) ion with a rapid response, excellent binding constant (3.38 μM), high selectivity, and sensitivity. Moreover, probe **V7** was successfully employed in real aqueous samples and fluorescent imaging for the detection of Ag (I) ions in living cells and zebrafish larvae with low cytotoxicity. All these results demonstrated its promising application prospects for Ag (I) sensing in both environmental and biological fields [484].

Fluorescent probe **V8** based on the peptide receptor could sensitively and selectively detect Cu (I) ions among various biologically relevant metal ions in aqueous solutions at a physiological pH through a ratiometric response. The binding affinity of probe **V8** to Cu (I) ions was 5.73 × 10^−21^ M^2^, and a 2:1 complex was formed. Probe **V8** successfully penetrated living A549 cells and detected intracellular Cu (I) ions in the Golgi apparatus through a ratiometric response [485].

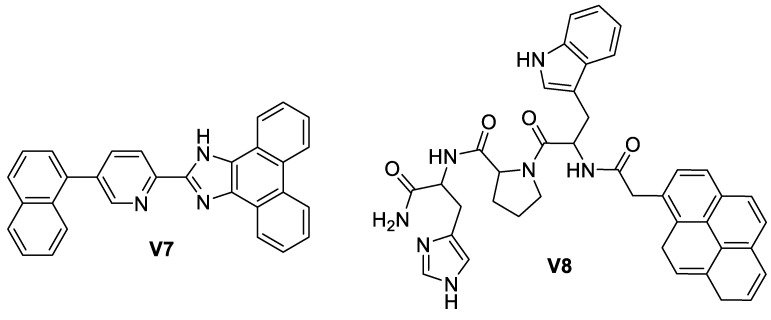



Instruments for the quantitative detection of palladium ions are expensive and complex to operate, so the development of palladium-detection probes, especially for imaging biological systems and the detection of trace metal residues in air, water, food, beverages, etc., is important. Thus, imidazole derivative **V9** bearing a thiophene group was developed as an on-off fluorescent reversible chemo-sensor for palladium ions. The absorbance intensity of probe **V9** was considerably enhanced, the fluorescence emission intensity was quenched in the presence of Pd (II) ions, and the presence of other metal ions had no notable interference. The detection of intracellular Pd (II) ions in living cells was performed using probe **V9** on brine shrimp nauplii (Artemia salina), up to 20 μg/mL [486].

Tripyridyl imidazole molecule **V10** has been developed as a probe for the dual sensing of Hg (II) and Cu (II) ions in an EtOH/HEPES buffer medium (5 mM, pH = 7.34, 1/1, *v*/*v*). Probe **V10** showed a good sensitive and selective turn-off response in the presence of both Hg (II) and Cu (II) ions. The probe could detect Cu (II) ions in the pH range of 3–11 and Hg (II) ions in the pH range of 6–8. The limit of detection (LOD) values toward Hg (II) and Cu (II) ions were 0.77 and 1.58 μM, respectively. Cell imaging studies using HDF and MDA-MB-231 cells also supported the viability of probe **V10** in detecting Hg (II) and Cu (II) ions in living cells [487].

The major disadvantages of many reported Zn (II) sensors are the interference from other transition metal ions, particularly Cd (II) ions, and the tedious multistep syntheses. Therefore, the synthesis of a highly sensitive and selective sensor probe for the detection of trace amounts of Zn (II) ions (especially intracellular) is important. Fluorescent receptor **V11** was synthesized by an easy condensation reaction of ortho-vanillin and 1-(3-aminopropyl) imidazole. Probe **V11** was found to be highly selective and sensitive toward Zn (II) ions in the presence of a wide range of metal cations, which exhibited fluorescence turn-on behavior with Zn (II) ions. The interaction of probe **V11** with Zn (II) ions showed a distinct fluorescence enhancement (turn-on) at 470 nm. Moreover, probe **V11** toward HeLa cancer cells was found to essentially be non-cytotoxic, which enabled the intracellular sensing of **V11** toward Zn (II) ions in the HeLa and DU-145 cancer cell lines [488].

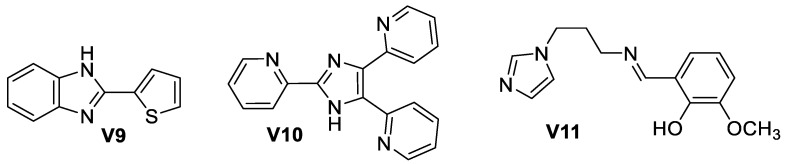



The development of peptide receptor-based fluorescence ratio probes that mimic the binding sites of metalloprotein for metal ions provides a potential tool for the detection of intracellular metal ions in living cells. Fluorescent probe **V12**, based on the symmetric peptide receptor bearing two imidazole and two sulfonamide groups, was used for the ratiometric detection of Zn (II) ions in an aqueous solution. Probe **V12** selectively and sensitively detected Zn (II) ions among biologically relevant metal ions by a ratiometric response that formed a 1:1 supramolecular complex with the Zn (II) ion of a picomolar binding affinity (300 pM). Moreover, the fluorescent peptidyl probe penetrated and successfully detected intracellular Zn (II) ions in live cells [489].

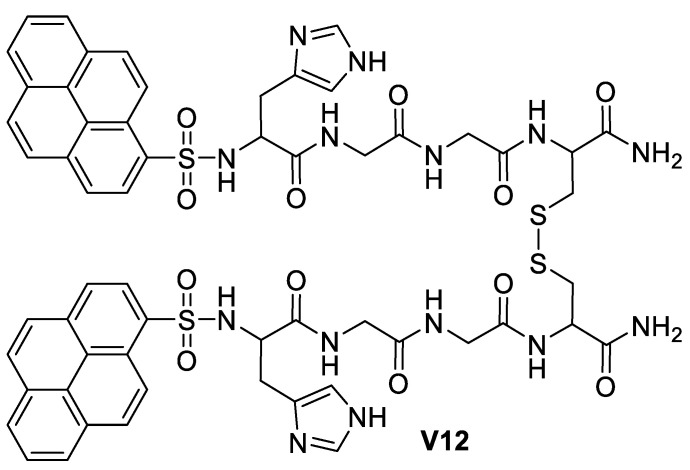



### 5.3. Imidazole-Based Supermolecules as Other Pathological Probes 

In other ways, related substances such as various ions could bind with the nitrogen atoms in imidazole molecules to form imidazole supermolecules that are used as probes to detect their concentrations in aqueous solutions. 

#### 5.3.1. Imidazole-Based Supermolecules as Probes to Detect the Change in pH

Since the pH value affects almost all aspects of cell and biological function, in biology, biochemistry, and many other research fields, it is necessary to carry out simple, intuitive, sensitive, and stable detection of the pH value inside and outside cells [490].

Ruthenium (II) complexes containing an imidazole group have been studied with respect to reversible acid–base interconversion, which may induce a large energy perturbation because they are good π donors and poor π receptors. In order to obtain ^1^O_2_-responsive fluorescent probes with pH-sensitive functionality, a binuclear Ru (II) complex **V13** containing anthryl and imidazole groups was designed and synthesized, which was an “on-off”-type fluorescent pH sensor. The ground- and excited-state acidity ionization constants have been determined by UV–visible spectrophotometric pH titration to be pK_a1_ = 1.12 ± 0.15, pK_a2_ = 6.90 ± 0.24, pK_a1_* = 1.09, and pK_a2_* = 6.92 [491].

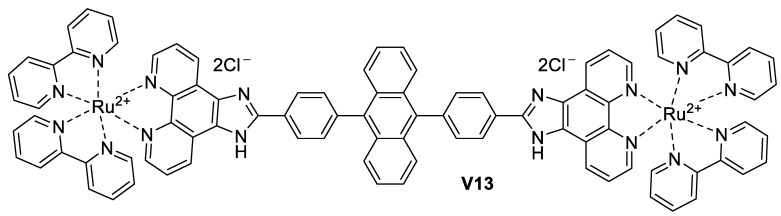



#### 5.3.2. Imidazole-Based Supermolecules as Probes to Detect Fluoride Ions

Fluoride ions play an important role in a lot of physiological activities related to biological and medical systems, such as water fluoridation, caries treatment, and bone disease treatment [492]. Fluorine-ion fluorescent probes with high selectivity, high sensitivity, and a fast response have broad application prospects for the detection of fluoride ions.

Fluorescein dyes are extensively used as bio-labels and sensors because of their tremendous photo-physical properties, such as visible region absorption/emission, high fluorescence quantum yield, and enhanced photostability. Fluorescein dye compound **V14**, containing imidazole, could be used as an effective colorimetric agent and the fluorescent chemical sensor for fluoride ions. By forming a hydrogen bond with the nitrogen atom in the imidazole ring, the fluorine ion triggered the opening of compound **V14**’s helical lactam ring, resulting in the color of the fluorine ions changing from colorless to orange, emitting a green fluorescence. Probe **V14** was further utilized to image fluoride in live cells by fluorescence imaging [493].

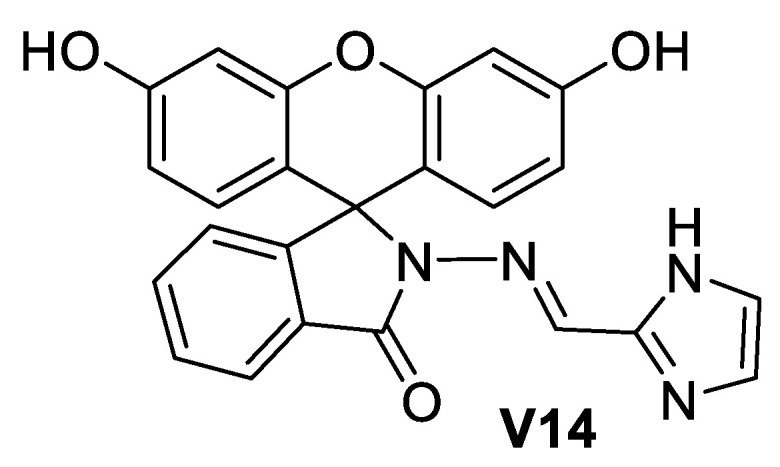



#### 5.3.3. Imidazole-Based Supermolecules as Probes to Detect Hydrogen Sulfide (H_2_S)

H_2_S is well-known as a colorless toxic gas that can be found as HS^−^ in rivers and waste waters, especially in the occupational susceptible environment. Studies have found that H_2_S is also an endogenous signaling molecule with important biological functions, but its physiological and pathological mechanisms remain unclear [494]. Therefore, enthusiasm for the detection of H_2_S concentration in vivo and the study of pathogenic mechanisms still exists.

Bipyridyl imidazole derivative **V15** had an imidazole ring and two pyridine rings, which could form coordination bonds with Cu (II) ions, resulting in fluorescence quenching. Compound **V15** produced fluorescence at 410 nm after excitation at 280 nm. It was found that HS^−^ ions selectively liberated compound **V15** from the complex via the formation of CuS. Probe **V15** had the advantages of a simple synthesis, better selectivity to hydrogen sulfide than other ions and sulfur or mercaptan compounds, and renewability. This reaction was used for the development of a fluorescence microplate assay for the determination of HS^−^ ions in environmental samples [495].

A series of fluorescent zinc complexes **V16a–d** could interact with HS^−^ ions. Through spectroscopic technology, it was proven that HS^−^ ions coordinated zinc in the target compound’s center, and the research result highlighted the potential of the designed system as a HS^−^/H_2_S fluorescence sensor through the coordination method. Further imaging experiments showed that these compounds had the potential to be used as H_2_S-detection probes in living cells. The target compounds could be successfully used as H_2_S sensors using a “coordination-based” approach [496].

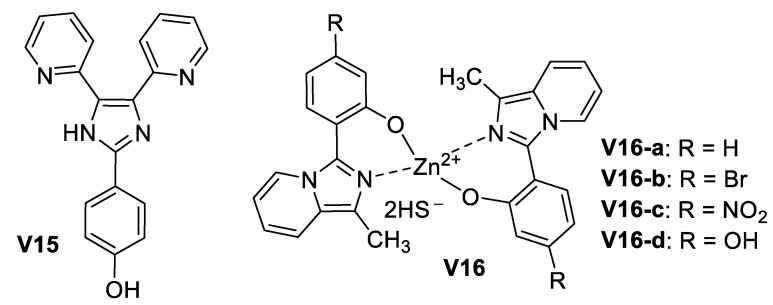



#### 5.3.4. Imidazole-Based Supermolecules as Probes to Detect Pyrophosphate Ions

Pyrophosphate anions play a key role in biological and chemical processes [497]. The development of rapid and convenient methods to detect pyrophosphate concentration is a research hotspot.

Methylene-bridged benzimidazole-substituted complex **V17** had a high selectivity for pyrophosphate ions (H_2_P_2_O_7_^2−^) in the photoluminescence channel, which was superior to other competitive phosphates such as H_2_PO_4_^−^ ions and ATP, ADP, and AMP plasma. Probe **V17** had low cytotoxicity, and the cell viability of the HeLa cells remained above 80% at 100 μM. This was the first report that Ir (III) complexes with 1,2,3-triazole ligands could conduct highly selective luminescence detection of H_2_P_2_O_7_^2−^ ions through multiple hydrogen-bond interactions [498].

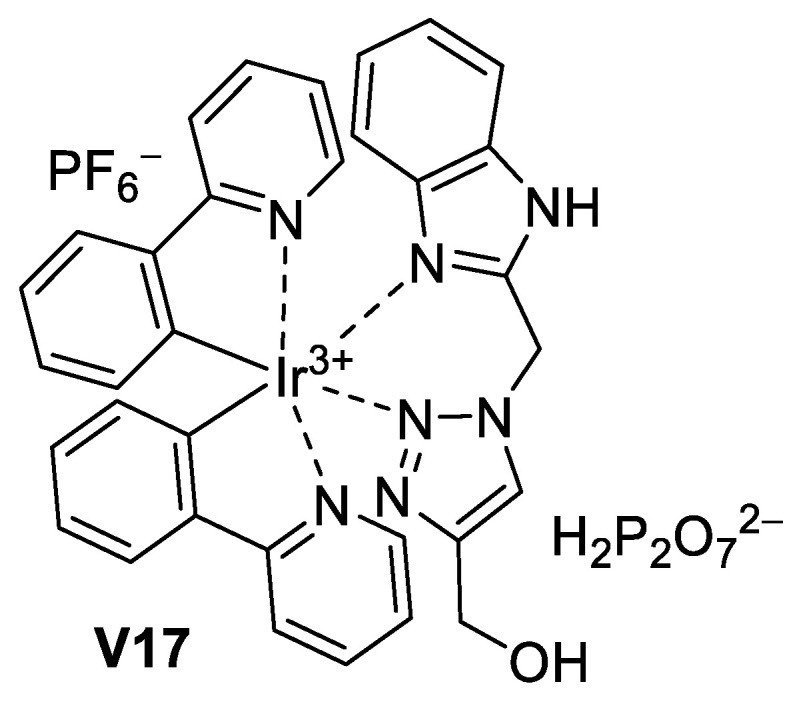



#### 5.3.5. Imidazole-Based Supermolecules as Probes to Detect Silver Ions

The silver ion is one of the most toxic forms of heavy metals [499]. Due to low selectivity and low quantifying factors, the development of innovative fluorescent probes with a low detection limit and high selectivity has become crucial [500]. It is necessary to find a fast and efficient method to detect the concentration of silver ions. 

Benzimidazole derivative **V18**, as a fluorescent active compound, had excellent selectivity toward Ag (I) ions in aqueous media. Probe **V18** provided ultrafast detection (<30 s), even for a very low concentration of Ag (I) ions, with good linear correlation, making it a practical sensor for the detection of silver ions [501]. 

Imidazole-substituted phenanthroline-derivative **V19** capable of recognizing Ag (I) ions under a physiological pH exhibited selectivity toward Ag (I) ions in DMSO-H_2_O (1/9, *v*/*v*) in 50 mM HEPES (4-(2-hydroxyethyl)-1-piperazineethanesulfonic acid) at a pH of 7.4. Moreover, the detection of probe **V19** toward Ag (I) ions could be realized with the “naked eye” by a color conversion from greenish yellow to orange. The limit of detection and the association constant (Ka) of the **V19**-Ag (I) complex were 1.8 nM and 8.24 × 10^5^ M^−1^, respectively. Furthermore, the **V19**-Ag (I) complex subsequently acted as a secondary fluorescent “turn-on” probe for F^−^ ions recognition among the other anions. The results showed that the molecular recognition system had potential applications in physiological and environmental systems for the detection of Ag (I) and F^−^ ions [500].

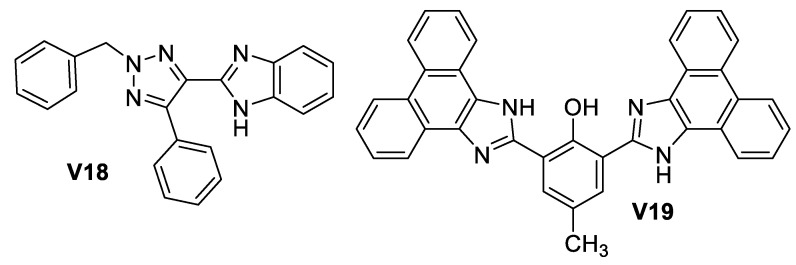



#### 5.3.6. Imidazole-Based Supermolecules as Probes to Detect Mercury Ions

Mercury is a highly toxic pollutant with biological accumulation in the environment. Hg (II) ion contamination can accumulate along the food chain and pose a serious threat to public health. Therefore, a great deal of research work has been devoted to developing fast, sensitive, and selective fluorescent probes for the detection of Hg (II) ions [502].

Reversible naphthalimide-based probe **V20** showed high sensitivity and was a selective “naked-eye” chemosensor for Hg (II) ions in a phosphate buffer, which had almost no response to other transition metal ions. The fluorescence of probe **V20** could be quenched up to 90% by the addition of Hg (II) ions. Reversible probes can detect Hg (II) ions over a wide pH range (7.0–10.0). These selective and sensitive results may lead to potential applications in managing environmental pollution and the detection of Hg (II) ions in biomedical samples [503].

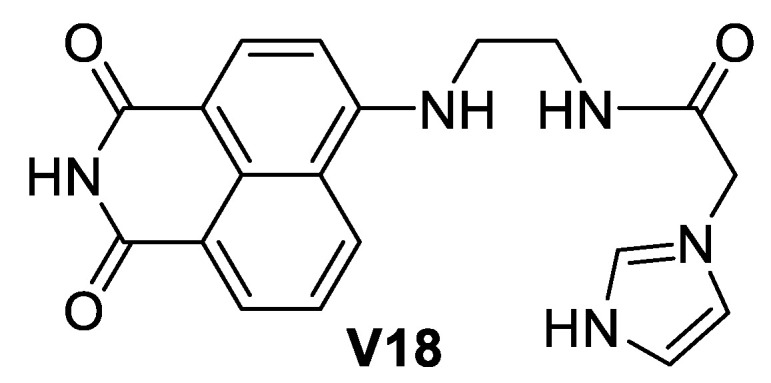



#### 5.3.7. Imidazole-Based Supermolecules as Probes to Detect Zinc Ions

Zinc is important for humans and other biological systems. High or low concentrations of Zn (II) ions in humans can lead to various diseases, and the improper treatment of Zn (II) ions can lead to environmental pollution. Given the importance of this metal, a number of convenient, low-cost, and easy-to-operate fluorescent probes for the detection of Zn (II) ions have been developed [504].

Imidazolyl Schiff base compound **V21** showed a selective sensing ability toward Zn (II) ions as a pyrogenic sensor. In the presence of Zn (II) ions, measurable fluorescence signals were generated at 612 nm, accompanied with bath color enhancement. The sensitivity of the fluorescence method for the determination of Zn (II) ions (6.78 × 10^−9^ M) was far lower than the World Health Organization’s drinking water guidelines (7.6 × 10^−5^ M), so it could become a practical system for monitoring the concentration of Zn (II) ions in water samples [505].

Owing to its 3d^10^4s^0^ valence shell electronic configuration, the zinc (II) ion is spectroscopically and magnetically inactive. Therefore, switch-on fluorescent probes for the sensing and monitoring of Zn (II) ions at nanomolar levels in real water samples with low background interference are in high demand. Imidazole-appended anthracene-based probe **V22** detected Zn (II) ions selectively in an 80% aqueous solution of dimethyl sulfoxide (DMSO) through a switch-on response at a pH of 7.5 without any interference from other metal ions. The switch-on response was driven by the selective complexation of **V22** with Zn (II) ions, mediated by the combined effects of PET and chelation-induced enhanced fluorescence, resulting in a more structurally rigid complex that detected H_2_PO_4_^−^ and CN^−^ ions sequentially through switch-off responses. The lowest detection limits for Zn (II), H_2_PO_4_^−^, and CN^−^ ions in 80% aqueous DMSO were determined to be 1.0 × 10^−9^, 1.0 × 10^−9^, and 8.0 × 10^−9^ M, respectively [506].

A tridentate dibenzimidazole-pyridine ligand with two pentyl side chains and its metal complex **V23** with Zn (II) ions were synthesized. This ligand showed a selective sensing ability toward Zn (II) ions, and the detection limit was calculated as 3.09 × 10^−7^ M [507].

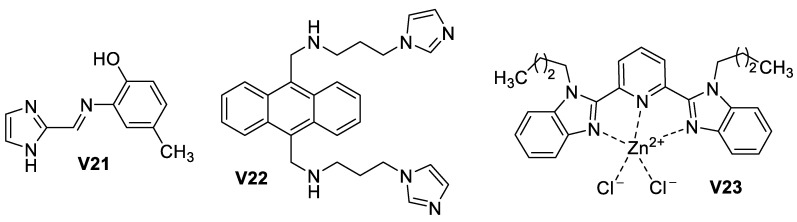



#### 5.3.8. Imidazole-Based Supermolecules as Probes to Detect Copper Ions

As is well-known, excess copper causes Wilson’s, prion, and Menkes diseases. The permitted concentration of Cu (II) ions in drinking water, according to the Environmental Protection Agency (EPA), is 20 μM. Therefore, it is important to develop an ultrasensitive and selective method for the determination of Cu (II) ions in environmental water samples [508]. 

Allyl-substituted imidazole derivative **V24** exhibited high selectivity and sensitivity toward the detection of Cu (II) ions through the formation of a 2:1 complex with Cu (II) ions. The detection limit for Cu (II) ions was 1.01 nM. Chemosensor **V24** was used as an efficient colorimetric and fluorometric nanoprobe for the quantitation of Cu (II) ions in real environmental water samples such as drinking water and mineral water, with good recoveries percentages and acceptable RSD percentages [508].

Dibenzimidazole derivative **V25** could act as a fluorescent chemical sensor and show a faster coordination effect with metal ions and a lower detection limitation than the known Cu (II) ions’ fluorescent molecule. The limit detection of the probe was low, down to 0.094 μM, with a complex constant of 6.57 × 10^−8^ M^−1^, which showed a high sensibility toward copper ions. In addition, this probe was used to detect copper ions in tap water, river water, and seawater, which demonstrated its potential application in seawater. More importantly, the response time of the probe to copper ions was within 1 s [509].

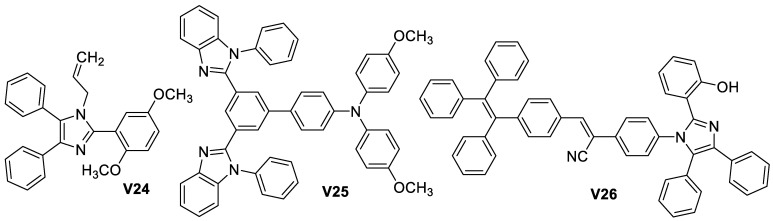



Tetraphenyl ethylene-functionalized aryl imidazole-derivative **V26** showed excellent aggregation-induced emission properties. The hydroxyl group and the N atom of imidazole were good binding sites for metal ions, making compound **V26** an excellent bidentate ligand. The unique molecular structure design enabled the special sensing of Cu (II) ions. The chelation of compound **V26** with Cu (II) ions could induce the formation of a stable Cu(**V26**)_2_ complex to quench fluorescence. Therefore, compound **V26** was also used to detect Cu (II) ions in an aqueous solution medium, with an LOD of 34.8 nM [510].

#### 5.3.9. Imidazole-Based Supermolecules as Probes to Detect Iron Ions

Iron is one of the most important elements in the metabolic process and is indispensable to all life systems, so it is widely distributed in the environment and biomaterials. However, concentrations in humans below or above the normally permissible limits can cause serious disease. Therefore, there is a need to develop fluorescent probes that can easily and quickly detect Fe (III) ions through fluorescence enhancement to understand the physiological and pathological contributions of Fe (III) ions to living systems [511].

Fluorescence “on-off” aniline-derived imidazole probe **V27** was synthesized via a soft and high-efficiency one-pot microwave-assisted method under solvent-free conditions. It was found that probe **V27** exhibited high selectivity and sensitivity toward Fe (III) ions. Probe **V27** could bind with Fe (III) ions in a ratio of 1:1 to form a supramolecular complex. The addition of Fe (III) ions to probe **V27** in DMF/H_2_O (2:3) displayed significant fluorescence quenching at 388 nm. Moreover, the detection limit for Fe (III) ions was calculated to be 0.72 μmol/L, along with a relatively fast response time (30 min) [512].

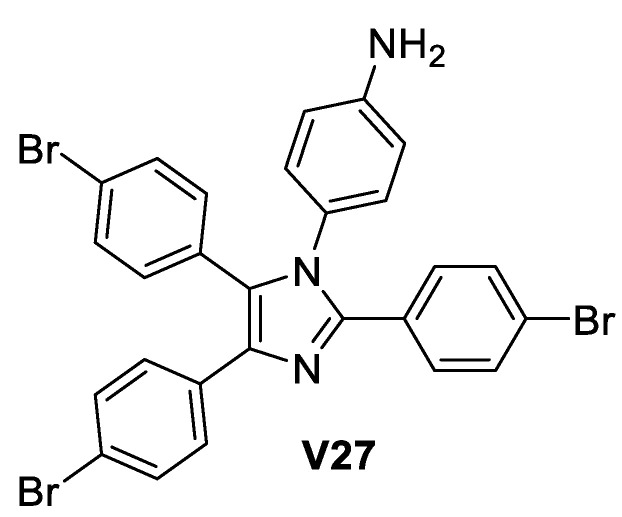



## 6. Conclusions and Outlook

The above systematic summary and overview revealed that imidazole-based supramolecular complexes have been widely studied and showed great medicinal potential as medicinal agents, ion receptors, imaging agents, pathologic probes, etc. In particular, research on medicinal agents is increasingly expanding toward imidazole-based supramolecular complexes; it has covered many medicinal aspects including anticancer, antibacterial, antifungal, antiparasitic, antidiabetic, antihypertensive, anti-inflammatory, and antitubercular potential. Some imidazole supramolecular complexes have shown good biological activity and a large medicinal possibility with good solubility, low toxicity, low drug-resistance, and high efficacy characteristics. Importantly, unique structural imidazoles with special functional groups can produce a variety of multipotential supermolecules by noncovalent bonds such as coordination bonds, hydrogen bonds, ionic bonds, van der Waals forces, etc. The formation of imidazole supermolecules with biomolecules such as enzymes, receptors, DNA, RNA, etc., can interfere with the function of biomolecules in biological systems to exert broad-spectrum bioactivities, thus regulating life function and further protecting human health. Imidazole supermolecules derived from metal ions including noble metals, transitional metals, and other metals as well as non-metals have shown a variety of potential applications not only as supramolecular medicinal agents for various diseases but also as cation and anion receptors for recognition and separation, as imaging agents to generate high-resolution images of cells and tissues with real-time data acquisition, and as pathological probes that are used as attractive tools for the detection of various chemical and biological components. All these preliminary investigations suggested a large potential of imidazole-based supramolecular complexes in a wide range of areas, which greatly promote more and more scientific concerns toward imidazole-based supramolecular complexes for all possible applications.

The enticing various medicinal potentials of imidazole supramolecular complexes will surely spur increasingly vigorous efforts toward the design, construction, structure, properties, and applicable medicinal potential of imidazole-derived supramolecular complexes. The related work will become more and more active, and some hot topics of the new trend of the foreseeable research in the near future toward imidazole-based medicinal supramolecular complexes might appear with the following aspects.

(1). The design of imidazole-derived supramolecular complexes with large medicinal druggability will become increasingly active.

The unique imidazole ring gives the strong ability of imidazole derivatives to demonstrate supramolecular behaviors. The multiple binding sites of the imidazole ring can easily form imidazole supramolecular complexes with various inorganic or organic ions and biomacromolecules such as enzymes, receptors, DNA, etc., through noncovalent interactions, showing not only the biological activity of the imidazole drugs themselves but also the many advantages of supramolecular drugs; naturally, these may exert dual or multiple mechanisms to overcome the serious, globally increasing drug resistance. 

In particular, the design of drug molecules based on supramolecular interaction will attract more and more attention. A design that can effectively interact with biological macromolecules to produce strong biological effects, to obtain highly active drug molecules, will be a challenging and great scientific topic.

(2). The efficient construction of medicinal imidazole supramolecular complexes will be an important research direction.

The economic preparation through the use of the imidazole ring, which can access the unique structural frameworks of medicinal imidazole supramolecular complexes, will be pursued aggressively for possible practical application. Imidazole derivatives with novel structures may exhibit better biological activity. The introduction of functional groups with a strong coordination ability and low toxicity and the search for highly active imidazole ligands are important components in the development of medicinal imidazole supermolecules. 

(3). The supramolecular structures of medicinal imidazole supermolecules will attract much interest.

The assembly and generation of unique supramolecular structures through multiple coordination modes, especially multitargeting ligands with unique mechanisms of action, will attract more attention because they are beneficial for affording the high potential of medicinal imidazole supermolecules.

(4). More and more efforts will be directed toward the relative ADMET property of medicinal imidazole supramolecular complexes.

ADME assessment is an important work of medicinal imidazole supramolecular complexes, so their investigation will help to discover the highly active medicinal imidazole molecules with satisfactory ADME properties including good solubility and bioavailability, low toxicity and side effects, and no drug resistance.

(5). Increasing contributions will target all possible medicinal potentials of imidazole supramolecular complexes.

The large potential for the clinical treatment of imidazole supramolecular complexes as medicinal agents will promote more and more efforts to investigate all possible medical potentials, extending beyond the current anticancer, antibacterial, antifungal, antiparasitic, antidiabetic, antihypertensive, anti-inflammatory, and antitubercular aspects to other medicinal areas.

The imidazole ring has multiple binding sites and can interact with various anions, cations, and biomolecules in the human body. Imidazole-based supermolecules, as artificial receptors and fluorescent molecules, will expand to extensively explore their possible applications in the biological systems of diagnostic and pathological probes.

(6). The study of the structure-activity relationship of imidazole-based supramolecular complexes will be increasingly important.

The study of the relationship between the structure of imidazole-based supermolecules and their water solubility, liposolubility, and biological activity is conducive to the development of new medicinal imidazole supermolecules.

(7). The discovery of novel and unique supramolecular drug mechanisms and the identification of supramolecular drug targets will become increasingly prominent.

It is very important to reveal new action mechanisms and identify the targets for new imidazole drug discovery.

There is no doubt that the further expansion of research toward imidazole-based supramolecular complexes will promote the development of more and more imidazole-based drugs with better activity, lower toxicity, and better pharmacokinetic properties as well as more effective diagnostic agents and pathological probes for further clinical medicinal use in contributions to the sustainable and healthy development of humankind.

## Data Availability

Not applicable.
